# Holistic Assessment of Factors Associated with Exhaustion, the Main Symptom of Burnout: A Meta-Analysis of Longitudinal Studies

**DOI:** 10.3390/ijerph192013037

**Published:** 2022-10-11

**Authors:** Yara Shoman, Valentin Rousson, Renzo Bianchi, Irina Guseva Canu

**Affiliations:** 1 Center of Primary Care and Public Health (Unisanté), University of Lausanne, 1066 Epalinges-Lausanne, Switzerland; 2 Department of Psychology, Norwegian University of Science and Technology (NTNU), 7491 Trondheim, Norway

**Keywords:** exposure-response relationship, latency period, outcome measurement, predictor, exposure assessment, stress

## Abstract

Background: This meta-analysis summarized longitudinal findings pertaining to exhaustion’s predictors. In so doing, our aim was ultimately to identify target factors for the prevention of burnout. Methods: We searched for studies that (a) examined predictors of exhaustion longitudinally and (b) reported correlation coefficients as an effect estimate. We conducted our literature search in three databases: MEDLINE, PsycINFO, and Embase. We focused on studies published between January 1990 and November 2020. Predictors were grouped into families, subfamilies, and subgroups. A meta-analysis of z-transformed correlation coefficients (rho) was performed. The results were scrutinized in relation to studies’ follow-up length. Results: We included 65 studies assessing 242 predictors of different types captured across different occupations. Our findings highlighted mostly weak associations (rho < 0.30). For six predictors—Job control, Job resources, Interactions at work, Communication and leadership, Job attitudes, and Work-family interface—longer length of follow-up involved weaker associations with exhaustion. The quality of the evidence available was generally low. Conclusions: The evidence available does not point to clear target factors for preventing burnout. The decrease in associations as the follow-up length increases may suggest a relatively short latency period, followed by recovery. Higher-quality cohorts should be conducted to better understand the etiology and course of burnout.

## 1. Introduction

Occupational burnout is not recognized as a medical condition in either the 11th edition of the International Classification of Diseases (ICD-11; [1]) or the 5th edition of the Diagnostic and Statistical Manual of Mental Disorders [2]. Though burnout remains nosologically and diagnostically uncharacterized, a recent harmonized definition of the entity identifies it with a state of “physical and psychological exhaustion due to prolonged exposure to work-related problems” ([3], p. 95). Exhaustion has long been considered the core symptom of burnout (e.g., [4]) and constitutes the only consensual characteristic of burnout [5,6].

Exhaustion is the best-measured aspect of burnout by the currently available Patient-Reported Outcome Measures (PROMs; see [7]). Some PROMs, such as the Copenhagen Burnout Inventory (CBI; [6]) and Shirom Melamed Burnout Measure (SMBM; [5]), equate burnout with exhaustion. Other PROMs, such as the Maslach Burnout Inventory (MBI; [8]) and Oldenburg Burnout Inventory (OLBI; [9]), consider burnout to involve additional symptoms (e.g., disengagement). These additional symptoms, however, are generally considered secondary. Conceptually, it is believed that exhaustion is the first symptom of burnout to develop in a worker in response to work stressors [10]. Furthermore, exhaustion has been conceived of as a symptom that is both psychological and physical. From a prevention perspective, it is more effective to focus on the early symptoms rather than the more advanced ones [11]. Thus, by preventing exhaustion, we may prevent the worker from developing other symptoms such as depersonalization or disengagement. Additionally, focusing on exhaustion can help increase inclusiveness when performing a meta-analysis by harmonizing the definition of the outcome (i.e., exhaustion).

A large body of literature addresses the determinants of burnout. However, no systematic review with meta-analysis has summarized the evidence derived from these studies in a comprehensive manner. Heterogeneity is a crucial issue in both qualitative and quantitative systematic reviews. This might explain why the meta-analyses performed until now focused on only certain types of determinants or occupations [12,13,14,15,16,17]. A recent meta-analysis of 48 longitudinal studies examining the relationships between job stressors and burnout concluded that unobserved heterogeneity was an important limitation [18]. The meta-analysis in question [18] focused only on job stressors. Its authors concluded that job stressors such as workload have only small effects on burnout, suggesting the existence of many other determinants of burnout. Although it is useful to thoroughly examine certain types of determinants or occupations, we believe it is indispensable to consider the determinants of burnout globally and to pay attention to their respective importance. The work-related predictors are mainly studied in relation to burnout with many theoretical models [19]. On the other hand, non-work-related factors are less studied. Although based on the person-job misfit theory [4], individual dispositions (e.g., personality traits) are expected to play a role in burnout’s etiology (e.g., by contributing to shaping job experiences, including job stress experiences). Moreover, only two systematic reviews focused on the exhaustion symptom of burnout [20,21] and these two studies did not perform a meta-analysis.

Another gap in the literature on burnout’s etiology concerns latency. In occupational epidemiology, latency is defined as the time between the first exposure and the development of the symptoms of interest [22]. However, there are different definitions of latency in the epidemiological literature [23]. For instance, Rothman [24] defines latency as “the period between disease initiation and disease detection”. This review will target studies that assessed the associations between exhaustion and its putative predictors longitudinally. These studies will thus not be able to address latency. However, we will assess the effect of follow-up length as an approximation of latency.

It is noteworthy that estimating the incidence or prevalence of burnout remains challenging given the nosological and diagnostic blur surrounding this entity [25,26]. In such a context, focusing on studies that measure burnout as a continuous (rather than a dichotomous) variable is warranted.

Considering the abovementioned gaps in our knowledge of burnout’s etiology, we decided to re-examine the literature using a holistic approach. Understanding the etiology of a phenomenon is crucial before planning preventive strategies [27]. In other words, to build successful preventive strategies for burnout, we should have knowledge of its determinants and focus on the most impactful ones. Preventing burnout is highly important because it may reduce the quality of life of the affected worker [28]. Indeed, research suggests that burnout can be a predictor of both self-reported and medically certified sickness leaves [29,30]. Moreover, burnout may threaten job performance, with potentially serious consequences (e.g., medical errors among health professionals). All in all, preventing burnout may be beneficial for reducing costs in healthcare and working life.

### Aims of the Study

We aimed to address the current gaps in our knowledge of burnout’s etiology in at least five ways. First, by focusing on the only consensual symptom of burnout, exhaustion. Second, by approaching exhaustion as a continuous outcome given the absence of diagnostic standards. Third, by comparing the effect sizes of work-related and work-unrelated predictors. Fourth, by including different occupational activities to generate more generalizable results. Fifth, by investigating the time-dependency of the associations between exhaustion and its predictors.

## 2. Materials and Methods

### 2.1. Protocol and Registration

The protocol of this study is available on the international database PROSPERO with the registration number CRD42021293031 from: https://www.crd.york.ac.uk/prospero/display_record.php?ID=CRD42021293031 (accessed on 24 December 2021). We conducted this study following the Conducting Systematic Reviews and Meta-Analyses of Observational Studies of Etiology (COSMOS-E) [31] and reported the results following the Preferred Reporting Items for Systematic Reviews and Meta-Analyses (PRISMA) statement [32].

### 2.2. Literature Search

**Figure 1:**
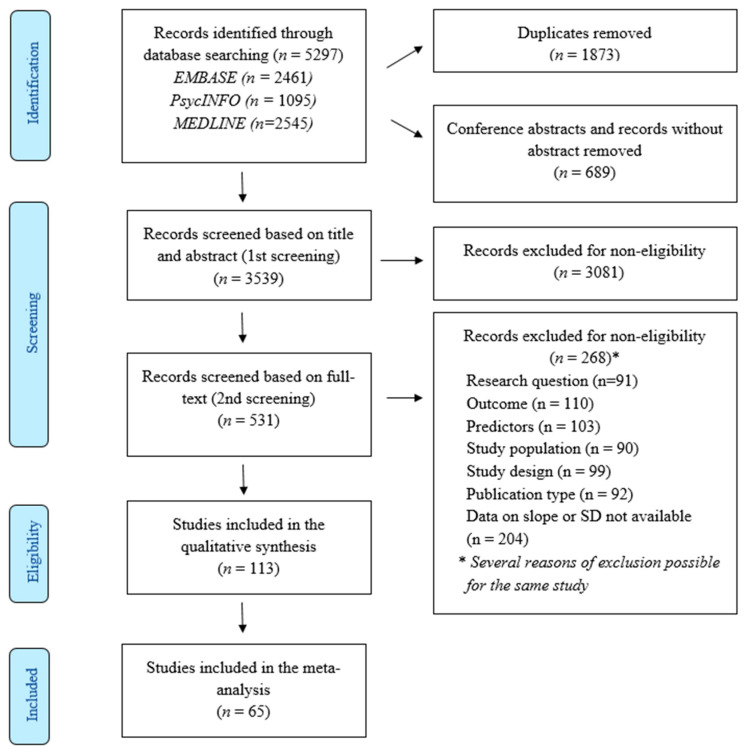
Flow-chart of the included studies.

The literature search was conducted in three databases: MEDLINE, PsycINFO, and Embase (Figure 1). We included studies published between January 1990 and November 2020. Only longitudinal studies were considered. We searched for studies reporting correlation coefficients (rho) between a predictor at the first measurement point (baseline) and the outcome at the second measurement point (end of follow-up). We included studies written in English, German, French, Polish, Russian, or Spanish, displaying at least 50 workers per group. We excluded studies when their outcome was not exhaustion, and when the exposures were not relevant (e.g., measured at the same time as the outcome and not before). We also excluded studies based on study design (i.e., not longitudinal), publication type (i.e., conferences papers, letter to editors), and study population (e.g., non-workers). At least two independent reviewers performed the screening for the inclusion of studies in our review independently, and when needed, a third reviewer was consulted. The screening was performed using Rayyan software [33].

### 2.3. Data Extraction

We extracted the following data from the selected studies:

The Pearson correlations between the predictor at the first measurement point and the outcome (i.e., exhaustion) at the second measurement point, the sample mean age, the gender distribution in the sample, the follow-up length, the number of follow-up measurement points, the type of the occupational activity of the participants included in the sample, and the country where the studies were conducted. Data extraction was conducted by one reviewer and 20% was cross-checked by another.

### 2.4. Meta-Analysis

**Figure 2:**
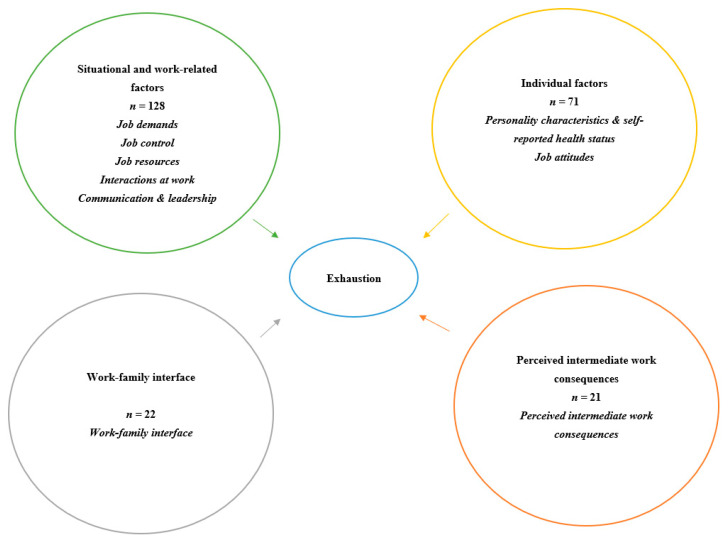
Description of the grouping of predictors in four families and nine subfamilies, shown in italics.

First, we grouped the predictors included in the different studies into four main families (Figure 2). Then, we grouped predictors in each family into subfamilies (Figure 2); we had five subfamilies for the first family, two for the second, one for the third and one for the fourth family, yielding nine subfamilies in total. The grouping of the predictors into families and subfamilies was carried out following a recent systematic review [20]. Eventually, we grouped predictors into families first, then subfamilies, then subgroups. In this sense, for some families there were not enough predictors to group into subfamilies, so we created only one subfamily and gave it the same name of the family in order to harmonize it with other subfamilies. We used the theoretical models that were used in the publication [7] to group the predictors. For example, we defined job control in this review following Karasek’s definition: “a ‘composite of two empirical but theoretically distinct constructs-the worker’s authority to make decisions (decision authority) and the breadth of skills used by the worker on the job (skills discretion)” [34]. In this context, the subgroup job control includes decision authority and skills discretion. Another example is the definition of the reward subgroup following Siegrist’s definition, implying esteem, promotion and security [35], and thus the subgroup includes these three elements. The considered subfamilies were: Job demands, Job control, Job resources, Interactions at work, Communication and leadership, Personality characteristics & self-reported health status, Job attitudes, Work-family interface, and Perceived intermediate work consequences. These subfamilies of predictors were then further subdivided into 66 subgroups of predictors to increase homogeneity (Figures S1–S9). The coefficients rho are known to be non-normally distributed, a Fisher’s z-transformation has been thus applied [36]. The meta-analysis was then conducted on such a transformed scale, where the standard error of a transformed estimate from a study is given by se = sqrt(1/(*n* − 3)), *n* being the sample size of the study [37], before being back-transformed on the original scale. We modeled the study-group specific z-transformed coefficients using a restricted maximization likelihood model with the study ID as a random effect. To avoid possible dependency issues [38], only one analysis was included from a study that examined the association of exhaustion and different predictor variables (from the same subgroup of predictor variables and involving the same individuals). If a study included more than one predictor variable that was to be grouped in the same subfamily, we included only one predictor variable which was the most comparable to other predictors in the subfamily. Consequently, we chose the most studied predictor variable in the literature. In the case of one study which included repeated measurement points, we chose to include the association representing the longest follow-up length in the analysis. We started to perform a meta-analysis for each subfamily of predictor variables. If the heterogeneity was too high (I^2^ larger than 60%), we performed a meta-analysis for each subgroup of predictor variables in that subfamily [38]. The strength of association was measured via the summary estimates (rho) and rated following the labels used in the field of psychology (i.e., 0: zero; ±0.1, ±0.3: weak; ±0.4, ±0.6: moderate; ±0.7, ±0.9 strong; and −1, 1 perfect) [39,40]. We assessed the publication bias using funnel plots and the Egger regression test [41,42]. This test is commonly used for testing the asymmetry in the funnel plot [43]. We performed the sensitivity analysis by excluding each study at a time and evaluating the impact by comparing the summary estimates (rho) and the heterogeneity (I^2^) before and after the exclusion of each study [44]. Moreover, we examined the effect of different follow-up lengths on the associations between predictors and exhaustion. We also performed an additional sensitivity analysis by meta-analyzing the subgroups only for studies conducted in Europe to observe if different countries would have an effect on the association between predictors and exhaustion. An additional sensitivity analysis by meta-analyzing the subgroups only for studies conducted on workers in the medical and health field was also performed. We chose to perform the sensitivity analysis for studies conducted in Europe and workers in the medical and health field because these two categories had the highest percentage among the 65 included studies, and we intended to ensure enough numbers for subgroup analysis. We also calculated prediction intervals for subgroups with at least ten studies as another major of heterogeneity. All statistical analyses were performed using Stata statistical software version 16 [45].

### 2.5. Risk of Bias and Overall Quality of Evidence

First, we assessed the risk of bias of the included studies individually using the Methodological Evaluation of Observational Research Checklist (MEVORECH) [46]. Secondly, we used the results of the risk of bias in the Grading of Recommendations Assessment, Development and Evaluation (GRADE) approach, in which the risk of bias is the first domain considered [47]. The GRADE was used for the overall quality of evidence of subfamilies, and it has five domains: risk of bias, inconsistency, indirectness, imprecision, and publication bias.

## 3. Results

### 3.1. Included Studies

Considering that occupational burnout is the result of prolonged exposure to workplace stress [1,3], we decided to exclude three studies that used a follow-up length of fewer than three months [48,49,50]. We therefore included 65 studies which met our inclusion criteria (Figure 1). We analyzed 66 samples included in these studies because one study included two different samples. Out of these 65 studies, 47 were conducted in Europe, 12 in North America, 4 in Asia, and 2 in Australia. These studies were conducted between 2001 and 2020, using different follow-up lengths and different numbers of follow-up measurement points (Supplementary Materials Table S1). The shortest length of follow-up was four months whilst the longest was ten years. The number of follow-up measurement points was two, three, or a maximum of four. Although all of the included studies were longitudinal using a multiple-follow-up measurements panel design, their design did not separate exposed from non-exposed participants and did not follow the participants to capture the onset of burnout. Therefore, they did not allow for capturing the onset of burnout, nor estimating the incidence of burnout or its symptoms and their latency. The panel or repeated cross-sectional study designs rather follow participants to observe the increase or decrease in burnout. Among included studies, 15% focused on nurses, another 15% focused on teachers, 3% on physicians, 17% on health and social service employees, and 18% on participants from different occupational sectors (mixed samples). The remaining studies included workers from other occupations. The mean age of the population included in the meta-analysis was 31 years and 75% of the participants were women. There were 34% of studies with a low risk of bias, 45% with a moderate risk of bias, and 21% with a high risk of bias. The included studies assessed the predictors and exhaustion using self-reported measurement tools only (Supplementary Table S1).

### 3.2. Meta-Analysis

We started performing the meta-analysis for each of the nine subfamilies of predictor variables (Figure 2), but we found a high heterogeneity for every subfamily (I^2^ was consistently much than 60% (Table 1 and Supplementary Materials Figures S1–S9)). Therefore, the summary estimates cannot be considered meaningful. Subgroup analysis allowed us to reduce the heterogeneity below 40%, and we found a moderate quality of evidence on the effect of six subgroups of predictors: Physical demands, Workload, Work agreements, Lack of support from coworkers, Stressful interactions with patients/students, and Lack of control (Table 1). However, the associations between each of these subgroups and exhaustion were weak (rho = 0.25, 0.38, −0.25, 0.27, 0.22, and 0.17, respectively).

For three subgroups of predictors (Job demands (overall), Quality of social interactions at work, and Psychological/physical toll), the evidence of association with exhaustion was low. The associations between Job demands (overall), Quality of social interactions and exhaustion were weak (rho = 0.33, −0.27). On the other hand, the association between Psychological/physical toll and burnout was moderate (rho = 0.44). Finally, among five other subgroups (Performance-based self-esteem, Avoidance-motives, Family work facilitation, Value congruency, and Job insecurity) with moderate heterogeneity (I^2^ values ranged from 40 to 60%), we found a moderate quality of evidence for a weak association for Job insecurity (rho = 0.16) and low to very low evidence for weak associations for the other four (Table 1). For the Job demands subfamily, the strongest association with exhaustion was found for Workload (rho = 0.38), followed by Time pressure and Job demands (rho = 0.35). Five subgroups (Job control, Skill discretion, Autonomy, Decision authority, and Flow experiences) of the Job control subfamily had protective effects against exhaustion whereas one subgroup showed a harmful effect (i.e., Lack of control) on exhaustion. However, the evidence for the associations of these five subgroups with exhaustion was low to very low while the evidence for the association of the Lack of control subgroup was moderate. The association with Job resources; and with Lack of job resources is the same in absolute value but has opposite directions (rho = 0.12). The associations between Reward and exhaustion; and Lack of reward/inequity and exhaustion were of very similar strength but in opposite directions (−0.32 for Reward-exhaustion and 0.35 for Lack of reward-exhaustion). For the Interactions at work subfamily, we found the strongest association between Fairness/justice and exhaustion (rho = −0.35). Indeed, as Fairness/justice increased, exhaustion decreased (protective effect). On the other hand, Conflict and interpersonal problems had the strongest harmful effect on exhaustion (rho = 0.30). When Conflict and interpersonal problems increased, so did exhaustion. For the Communication and leadership subfamily, the strongest association with exhaustion was observed for the Quality of social interactions at work (rho = −0.27). Thus, when the Quality of social interactions at work increased, exhaustion decreased. For the Personality characteristics and self-reported health status subfamily, only four subgroups showed protective effects against exhaustion but the evidence of the effects of three of them is very low despite a moderate risk of bias. For the Job attitudes subfamily, Positive and negative job attitudes have almost the same effect on exhaustion in absolute value (rho = −0.24 for positive job attitudes examined in seven studies, 0.25 for negative job attitudes examined in six studies). It is worth mentioning that despite the overall risk of bias being moderate and despite the number of studies being adequate, the evidence regarding the effects of these factors is very low. For the Work-family interface subfamily, the strongest associations were observed between exhaustion and Work-family conflict (rho = 0.36); and between exhaustion and Value congruency (rho = −0.27). Nevertheless, the evidence of these associations is low and very low, respectively. For the Perceived intermediate work consequences subfamily, Work stressors and satisfaction have the same effect on exhaustion in absolute values (rho = 0.24 for Work stressors and rho = −0.29 for satisfaction).

### 3.3. Effect of the Follow-Up Length on the Observed Associations

Table 2 and Supplementary Materials Figures S10–S18 show how the associations between subfamilies of predictor variables and exhaustion vary over time when using a longer follow-up length for each of the nine subfamilies. To better understand the Supplementary Figures S10–S18, let’s take an example the Job resources subfamily; Supplementary Figure S12 shows that the effect of Job resources decreased by almost four-fold after 12 months and six-fold after 36 months of follow-up length. In Supplementary Figure S12 the first column on the left is the study details (authors, year, predictor), then the point in the middle of the line represents the effect size (rho) while the line is the 95% confidence interval, the second column is the numeric effect size with 95% confidence interval followed by *p*-value and the follow-up length (in months). For the Job demands subfamily, the average effect size did not differ over different lengths of follow-up: 3 months (rho = 0.33), 6 months (rho = 0.38), nine months (rho = 0.39), 12 months (rho = 0.36), 18 months (rho = 0.35), 24 months (rho = 0.35), 36 months (rho = 0.33), and 48 months (rho = 0.33). Additionally, for the other two subfamilies (i.e., Personality characteristics & self-reported health status and Perceived intermediate work consequences), we observed no changes in the association between the subfamily of predictor variable and exhaustion when the follow-up length increased. However, for Job control, the average effect was almost two-fold lower after 12 months of follow-up. The effect of Job resources decreased by almost four folds after 12 months and six folds after 36 months of follow-up length. The average effect of the Interactions at work subfamily on exhaustion decreased by almost four folds when the follow-up length increased from six to 12 months. The effect of the Interactions at work subfamily on exhaustion decreased additionally by four folds after 24 months of follow-up length. For the Communication and leadership subfamily, the average effect on exhaustion decreased two-fold after 18 months of follow-up length. The average effect of Job attitudes on exhaustion was reduced by two-fold when the follow-up length increased from three to 12 months. For the Work-family interface, the average effect on exhaustion decreased by three-fold after 12 months of follow-up length.

### 3.4. Sensitivity Analysis and Additional Analyses

The sensitivity analysis allowed us to reduce heterogeneity for 25 subgroups (Supplementary Materials Table S2). However, the summary estimates after the sensitivity analysis showed weak associations between the subgroup of predictor variables and exhaustion with the exception of Reward, which had a moderate association with occupational burnout (rho = −0.42). We observed that the Self-esteem association (statistically significant) with exhaustion decreased from −0.33 to −0.23 when deleting one study [51]. The association between the Reward and exhaustion (statistically not significant) increased from −0.32 to −0.42 when deleting one study [52]. For the Work-family conflict subgroup, deleting one of the following four studies [53,54,55,56] reduced the heterogeneity from 49 to 0% with no change in the association strength.

The results of the additional sensitivity analysis by performing the meta-analysis of subgroups for studies either conducted in Europe or in workers in medical and health field did not show any significant changes in the results (Supplementary Materials Tables S3 and S4). Additionally, we calculated prediction intervals for three subgroups of predictors where the subgroup included at least 10 studies. These prediction intervals were: (−0.54, 0.19) for social support, (−0.39, 0.02) for self-efficacy and (0.27, 0.45) for work-family conflict. These wide prediction intervals indicate high heterogeneity. We note that there are no correlations higher than 0.60 in absolute value for these three subfamilies since the prediction intervals do not include this value.

### 3.5. Publication Bias

The funnel plots for all subfamilies showed that the studies were distributed on both sides of the plots (Supplementary Materials Table S5). All the *p*-values for the Regression-based Egger test for small-study effects per subfamily were not significant (Supplementary Materials Table S5). Consequently, we did not find any evidence for publication bias for these nine subfamilies of predictor variables.

## 4. Discussion

### 4.1. Main Findings

This meta-analysis aimed to identify exhaustion’s predictors. The meta-analysis included 65 studies and assessed 242 predictors. Our findings mainly highlighted high heterogeneity, relatively weak associations, and quite a low quality of evidence. Following the holistic approach, it is unsurprising that heterogeneity increased, but this approach helped include virtually all studies and compare different types of predictors, countries, occupational activities, and follow-up lengths. Unfortunately, even subgroup analyses did not yield better results. In some subgroups, the number of included studies was too small and thus the heterogeneity index could not be interpreted with confidence. Nevertheless, the weak associations observed in this meta-analysis are due to the effect size of the associations in the included studies where these associations were predominantly weak. The additional analysis of the effect of follow-up lengths on the associations between predictors and exhaustion suggests a variation in these associations’ strength between six sub-families of predictor variables and exhaustion with the increasing length of follow-up. In this regard, our study suggests that as the follow-up length increases, the effect of six out of nine subfamilies of predictor variables decreases (Job control, Job resources, Interactions at work, Communication and leadership, Job attitudes, and Work-family interface), which is an original and important finding to the best of our knowledge. These results may suggest a short latency period followed by a recovery.

### 4.2. Defining and Measuring Exhaustion

This meta-analysis identified a diversity of measurement tools for occupational burnout (Supplementary Materials Table S1). In an attempt to reduce this source of heterogeneity, we limited the outcome definition to exhaustion, in line with a recent harmonized definition of occupational burnout [3]. Nonetheless, we encountered a large variability in the measures of exhaustion (Supplementary Materials Table S1). Many researchers mentioned the heterogeneity in the definition and measurement of burnout when trying to combine available studies in a meta-analysis [26,57,58,59]. In addition to the aforementioned heterogeneity, there is the issue of whether burnout should be considered a diagnosis or not [60,61,62]. The diagnosis is difficult because the burnout phenomenon is still “medically unexplained” [63], and there are still no well-established biomarkers of burnout [63,64]. Although researchers started investigating the epigenetic correlates of burnout [63,65,66], these studies define occupational burnout using PROMs. Unfortunately, none of the available PROMs address how exhaustion develops over time in response to stress. Therefore, their usage in further studies would not answer the question of occupational burnout latency. New and more objective outcome measurement methods are necessary.

### 4.3. Defining and Measuring Predictors of Occupational Burnout

We included four main families of predictors in our study and each family was further divided into nine subfamilies, and the subfamilies into subgroups. We faced a methodological issue, which was the heterogeneity in defining and measuring the predictors. One example refers to Job demands and Job resources, which constitute the Job Demands Resource (JD-R) model that is amongst the most used models in research and practice [67,68]. Concerning the JD-R model, Schaufeli and Taris explained that there is a variation in representing the constructs of Job demands and Job resources because the model is not restricted to well-defined demands or resources but rather follows an open and heuristic approach [69]. Another example is Work-family conflict which can be assessed using many measurement tools developed based on different concepts and dimensions [70]. Self-efficacy was measured in ten studies (Supplementary Materials Table S1 and Figure S6), using ten different measurement tools (or their translated versions). These three examples illustrate the heterogeneity in the definition and measurement of almost all predictor variables of occupational burnout but also other mental health outcomes. Therefore, future research should use the best defined and harmonized definition of each predictor variable and the most valid methods of their measurement to enable the systematic assessment of their effects.

### 4.4. The Latency of Occupational Burnout

Occupational burnout is often considered a process that develops gradually [71] over years or even decades [72]. However, the time needed for burnout to develop (i.e., its “latency”) is not easy to capture and there is no consensus on the best follow-up length for burnout. Consequently, researchers conducted studies using different follow-up lengths (Supplementary Table S1). Although most of these studies included the same participants at all measurement points (i.e., using the panel study design), capturing the onset of burnout remains difficult because they did not identify the date when exposure started. Moreover, a panel study usually has a discontinuous follow-up, which makes it challenging to identify the length of the exposure until the onset of burnout.

We note that there are differences between subfamilies regarding the results of the effects of the different follow-up lengths on the associations between the predictors and exhaustion. Six subfamilies of predictors showed a change in the association as the follow-up length increased, whereas three subfamilies did not show any change. We could not interpret these results reliably but we suggest three hypotheses. One suggested hypothesis can be the heuristic model [69], which implies that similar interactions do not necessarily exist between all predictors and all outcomes, distinct from the strict cause-and-effect-models.

The “healthy worker survivor effect” could be one of the possible explanations as well. The healthy worker survivor effect describes an ongoing selection of workers such as those who stay employed tend to be healthier than those who leave their job [73]. Consequently, the workers who suffer from work-related stress and early burnout may leave their company and thus cannot be part of a study. Another possible explanation is that workers in the early stages of burnout who were correctly identified and treated develop either resilience or affective coping over time. The literature suggests that resilience protects workers from work-related stress or burnout [74,75,76,77]. One systematic review found that certain coping strategies can reduce stress in health professionals and caregivers [78]. A systematic review of predictor variables associated with return to work in burnout found one study that showed that covert coping has a negative association with return to work [79].

Ahola et al., concluded that the phenomenon of burnout is unstable and diminishes over time and that the majority of burned-out workers keep working [80]. On the other hand, burnout has been linked to a decline in specific cognitive functioning [81], even with long-lasting cognitive impairments in the non-clinical burnout stage (i.e., early stage of burnout) [82]. It is worth noting that the MBI was the most widely used measure of burnout in the studies included in our meta-analysis and the meta-analysis of Ahola et al. [80]. However, the MBI does not assess cognitive functioning. Thus, the long-lasting effects of burnout may not be detected.

To solve the question of burnout latency in a reasonable timeline, the question of the diagnosis of burnout should be solved. After reaching a consensus on how to diagnose burnout, comprehensive studies of burnout cases may be helpful, particularly those using comprehensive retrospective assessment of exposures, ideally using objective data on occupational, personal and organizational factors with their respective duration before burnout onset. A better understanding of burnout development in terms of symptoms and impairments associated with each of its severity degrees would also help in determining which stage corresponds to a clinical form and based on which criteria the latter could be defined or diagnosed. Once such criteria are established, a prospective cohort design would be the best option to identify the new burnout cases and to measure burnout incidence, instead of measuring prevalence in the currently available studies. With consistent data on burnout incidence, it should finally be possible to estimate the latency of burnout development in different exposure conditions and develop effective preventive interventions focusing on the most relevant exposure window.

### 4.5. Implications of the Findings

This meta-analysis showed that there is a change in the association between six subfamilies (out of nine subfamilies studied) and exhaustion as the follow-up lengths varied. We could observe a decline in the association for the subfamilies (Job control, Job resources, Interactions at work, Communication and leadership, Job attitudes, and Work-family interface) when the follow-up length increased. Consequently, this study highlights the need to capture and quantify the latency of occupational burnout onset which only can be carried out after finding a consensus on the diagnosis of burnout. This enables future studies to use the prospective cohort study design and calculate the incidence of occupational burnout, ultimately estimating the time between exposure to predictor variables and occupational burnout onset. Another implication of this study is showing the importance of targeting the six subgroups of exhaustion predictors (i.e., Physical demands, Workload, Work agreements, Lack of support from coworkers, Stressful interactions with patients/students, and Lack of control) when planning preventive strategies for occupational burnout. Exhaustion is a predictor of stress-related health outcomes, and it is the most studied predictor of burnout compared to its other dimensions [10]. Preventive strategies targeting exhaustion may thus be of great benefit to prevent occupational burnout.

This study confirms that a standardization of the measurement of occupational burnout is urgently needed. In addition, we recommend that potential predictors of burnout be investigated using more objective indicators. Higher-quality cohorts and qualitative studies of burned-out workers should be conducted to better understand the etiology and course of burnout, and improve the overall evidence regarding the new and the less studied predictors.

### 4.6. Conclusive Remarks

The holistic approach used in this study enabled us to systematically assess which predictors can be moderately associated with exhaustion but for which the evidence is still very low or low. For example, the associations with exhaustion for unvalued trait/characteristics and maladaptive coping were (rho = 0.32, 0.33, respectively) and the quality of evidence was very low. Therefore, this review highlights the gap of knowledge which is the predominance of work-related predictors over work-unrelated predictors. This meta-analysis examined 242 predictor variables; of which 62% were work-related, 9% were from the Work-individual interface and 29% were Individual predictor variables. These results are unsurprising considering that burnout is considered an occupational phenomenon and is assumed to result from work-related stress [1]. Burnout was considered as one of the occupational health outcomes and was mainly studied in the frame of job strain theoretical models such as Karasek’s, Siegrist’s or Demerouti’s models [35,83,84,85]. Therefore, the majority of occupational predictors studied are often limited to those ascribed to these models. In our meta-analysis, 33% of the predictors were from the Job demands, control, and resources subfamilies. However, it remains necessary to thoroughly examine the associations between non-work-related predictors and burnout in future longitudinal studies.

We observed high heterogeneity in defining and measuring exhaustion and the predictors in the included studies. We also observed the use of different follow-up lengths between measurement points. These factors can contribute as a source of heterogeneity in this meta-analysis. However, an additional source of heterogeneity can be the different populations used across the studies that were often implicitly selected. Nevertheless, this meta-analysis summarized the associations between nine subfamilies of predictors and exhaustion in 65 studies. A strength of this meta-analysis is its focus on longitudinal studies. This ensures the measurement of exposure at the beginning of the follow-up before the measurement of the outcome. The inclusion of studies over almost 30 years and the inclusion of both work-related and non-work-related predictors are two additional strengths of this work. Finally, we can mention the rigorous methodology applied and the novel approach centered on exhaustion among the strengths of this study.

## 5. Conclusions

This meta-analysis summarized longitudinal findings pertaining to exhaustion’s predictors. Our findings revealed mostly weak associations with limited overall evidence, reflecting the limitations of the included studies and the available literature. Unsurprisingly, following a holistic approach increased the heterogeneity but this approach remained beneficial in comparing the associations between different types of predictors and exhaustion. Unfortunately, the evidence available does not point to clear target factors for preventing burnout. The decrease in associations as the follow-up length increases may suggest a short latency period followed by recovery. Future research should benefit from using a prospective cohort study design to answer burnout’s etiological questions along with qualitative studies of burned-out workers.

## Figures and Tables

**Table 1:** Meta-analysis results per subgroups of studied predictor-variables.

Studied Predictor-Variables Grouped Per (Sub)Family	Number of Studies	Heterogeneity “I^2^ Estimate”	Summary Estimate of the Association with Exhaustion	95% Confidence Interval	Overall Risk of Bias Results	Inconsistency	Indirectness	Imprecision	Publication Bias	Overall Quality of Evidence ^1^
Job demands	27	89.25%	0.33	0.28–0.38						
Work and time demands	8	91.40%	0.33	0.22–0.43	Low	No	Yes	Yes	No	Low
Cognitive demands	3	89.74%	0.13	−0.05, 0.31	Moderate	Yes	No	Yes	No	Very low
Physical demands	2	0.00%	0.25	0.17, 0.34	Low	No	No	Yes	No	Moderate
Workload	6	18.69%	0.38	0.34–0.43	Moderate	No	No	No	No	Moderate
Time pressure	5	92.34%	0.35	0.17–0.53	Low	No	No	Yes	No	Moderate
Job demands (overall)	2	13.55%	0.35	0.23–0.48	Moderate	No	No	Yes	No	Low
Emotional demands	8	31.69%	0.34	0.30–0.39	Moderate	No	Yes	Yes	No	Very Low
Job control	20	94.14%	−0.15	−0.21, −0.09						
Job control	8	76.78%	−0.23	−0.30, −0.16	Low	No	Yes	Yes	No	Low
Skill discretion	3	0.00%	−0.05	−0.08, −0.02	High	No	Yes	Yes	No	Very low
Autonomy	6	77.82%	−0.21	−0.21, −0.11	Moderate	Yes	Yes	Yes	No	Very low
Decision authority	5	81.59%	−0.06	−0.19, 0.06	High	Yes	Yes	Yes	No	Very low
Flow experiences	1	NA	−0.40	−0.51, −0.29	High	NA	NA	NA	No	Very low
Lack of control	2	38.44%	0.17	0.07, 0.28	Low	No	No	Yes	No	Moderate
Job resources	11	97.22%	−0.07	−0.23, 0.08						
Job resources	6	97.75%	−0.12	−0.47, 0.22	Low	No	No	Yes	No	Moderate
Lack of job resources	4	73.40%	0.12	0.02, 0.23	Moderate	No	No	Yes	No	Low
Reward	3	83.64%	−0.32	−0.51, −0.12	Low	No	No	No	No	High
Lack of reward/inequity	2	96.27%	0.35	−0.12, 0.82	Low	No	No	Yes	No	Moderate
Material resources	3	72.77%	−0.27	−0.42, −0.13	Low	No	No	Yes	No	Moderate
Interactions at work	23	96.57%	−0.02	−0.10, 0.07						
Social support	12	89.24%	−0.18	−0.27, −0.08	Moderate	No	No	Yes	No	Low
Poor social climate	5	79.37%	0.24	0.12, 0.35	Low	No	No	Yes	No	Moderate
Support from supervisor	3	91.71%	−0.16	−0.29, −0.03	Low	No	Yes	Yes	No	Very low
Support from colleagues	3	0.01%	−0.16	−0.21, −0,12	Low	No	Yes	Yes	No	Very low
Fairness/justice	2	0.00%	−0.35	−0.45, −0.25	High	No	No	Yes	No	Very low
Lack of support from supervisor	2	85.25%	0.27	0.01, 0.52	Low	No	No	Yes	No	Moderate
Lack of support from coworkers	2	0.01%	0.27	0.20, 0.35	Low	No	No	Yes	No	Moderate
Conflict & interpersonal problems	3	92.19%	0.30	0.05, 0.55	Moderate	No	No	No	No	Moderate
Communication & leadership	12	93.09%	−0.13	−0.24, −0.03						
Work agreements	2	0.00%	−0.25	−0.33, −0.16	Low	No	No	Yes	No	Moderate
Communication/information flow	4	94.05%	−0.09	−0.30, 0.12	Moderate	No	Yes	Yes	No	Low
Quality of social interactions at work	3	20.20%	−0.27	−0.34, −0.19	Moderate	No	No	No	No	Low
Leadership	3	90.31%	−0.07	−0.31, 0.17	Low	Yes	Yes	Yes	No	Very low
Role conflict	1	NA	0.19	0.09, 0.29	Moderate	NA	NA	Yes	No	Low
Personality characteristics & self-reported health status	26	96.60%	−0.02	−0.11, 0.07						
Unvalued trait/ characteristics	3	90.29%	0.32	0.07, 0.57	High	No	Yes	Yes	No	Very low
Valued trait/ characteristics	5	88.15%	−0.24	−0.39, −0.09	Moderate	Yes	No	Yes	No	Very low
Extraversion	1	NA	0.13	−0.05, 0.31	Moderate	NA	NA	Yes	NA	Low
Conscientiousness	1	NA	−0.01	−0.19, 0.17	Moderate	NA	NA	Yes	NA	Low
Openness	1	NA	0.03	−0.15, 0.21	Moderate	NA	NA	Yes	NA	Low
Self-efficacy	10	70.20%	−0.19	−0.25, −0.12	Moderate	No	Yes	Yes	No	Very low
Maladaptive coping	3	0.00%	0.33	0.24, 0.42	Moderate	No	Yes	Yes	No	Very low
Adaptive coping	4	73.55%	−0.02	−0.16, 0.11	Moderate	Yes	Yes	Yes	No	Very low
Emotion-focused coping	2	87.42%	−0.02	−0.18, 0.14	Moderate	Yes	No	Yes	No	Very low
Self-esteem	2	83.22%	−0.33	−0.53, −0.13	Low	No	No	Yes	No	Moderate
Performance-based self-esteem	3	45.52%	0.24	0.20, 0.28	High	No	No	Yes	No	Low
Self-reported health status (harmful)	2	92.14%	0.34	0.13, 0.55	Moderate	No	No	Yes	No	Very low
Self-reported health status (protective)	1	NA	−0.33	−0.46, −0.20	High	NA	NA	Yes	NA	Very low
Job attitudes	18	95.73%	0.05	−0.04, 0.13						
Positive job attitudes	7	79.71%	−0.24	−0.33, −0.15	Moderate	Yes	Yes	Yes	No	Very low
Negative job attitudes	6	79.93%	0.25	0.17, 0.33	Moderate	Yes	Yes	Yes	No	Very low
Intrinsically motivated behavior	8	86.28%	−0.07	−0.17, 0.03	High	Yes	Yes	Yes	No	Very low
Extrinsically motivated behavior	4	83.30%	0.28	0.05, 0.51	Moderate	No	No	Yes	No	Low
Avoidance motives	2	54.33%	0.20	0.03, 0.37	High	No	Yes	Yes	No	Very low
Acquiescent silence	1	NA	0.22	0.14, 0.30	High	NA	NA	Yes	NA	Very low
Quiescent silence	1	NA	0.26	0.18, 0.34	High	NA	NA	Yes	NA	Very low
Prosocial silence	1	NA	0.01	−0.07, 0.09	High	NA	NA	Yes	NA	Very low
Opportunistic silence	1	NA	0.13	0.05, 0.21	High	NA	NA	Yes	NA	Very low
Work-family interface	11	98.35%	0.13	0.02, 0.23						
Work-family conflict	10	49.36%	0.36	0.33, 0.39	Moderate	No	Yes	No	No	Low
Family-work conflict	3	0.00%	0.20	0.17, 0.24	High	No	Yes	Yes	No	Very low
Work-family facilitation	3	71.24%	−0.11	−0.19, −0.02	High	No	No	Yes	No	Low
Family-work facilitation	3	57.95%	−0.05	−0.11, 0.02	High	Yes	No	Yes	No	Low
Value congruency	3	54.12%	−0.27	−0.34,−0.20	Moderate	Yes	No	Yes	No	Very low
Perceived intermediate work consequences	16	95.04%	0.19	0.09, 0.29						
Work stressors	4	80.55%	0.24	0.13, 0.35	Low	No	No	Yes	No	Moderate
Stressful interactions with patients/students	2	0.00%	0.22	0.16, 0.28	Low	No	No	Yes	No	Moderate
Job insecurity	2	56.18%	0.16	0.03, 0.30	Low	No	No	Yes	No	Moderate
Impact of change	2	90.29%	0.26	0.08, 0.44	Moderate	No	No	Yes	No	Low
Psychological/physical toll	2	33.39%	0.44	0.31, 0.56	Moderate	No	Yes	No	No	Low
Stress from work	3	93.06%	0.26	0.06, 0.46	Moderate	No	No	Yes	No	Low
Satisfaction	3	75.43%	−0.29	−0.47, −0.11	High	No	No	Yes	No	Very low
Colleagues/team exhaustion	2	88.04%	0.27	−0.10, 0.64	Moderate	No	No	Yes	No	Low

Note: ^1^ Based on the GRADE, which takes into account the risk of bias, inconsistency, indirectness, imprecision, and publication bias of all studies for a given predictor.

**Table 2:** Summary of the effect of different follow-up lengths on the association between subfamilies of predictors and exhaustion.

Subfamily	Change in the Associations between Predictors and Exhaustion	Change in Follow-Up Length (Months)
Job demands	No change	3–48
Job control	2-fold decrease	6–12
Job resources	2-fold decrease 6-fold decrease	6–12 12–36
Interactions at work and occupational burnout	4-fold decrease 4-fold decrease	6–12 12–24
Communication and leadership	2-fold decrease	3–18
Personality characteristics and self-reported health status	No change	3–48
Job attitudes	2-fold decrease	3–12
Work-life interface	3-fold decrease	6–36
Perceived intermediate work consequences	No change	6–120

## Data Availability

Data are available online as Supplementary Materials of the present article.

## Supplementary Materials

The following supporting information can be downloaded at: https://www.mdpi.com/article/10.3390/ijerph192013037/s1, Table S1: Description of the reviewed studies; Figure S1: Job demands forest plot; Figure S2: Job control forest plot; Figure S3: Job resources forest plot; Figure S4: Interactions at work forest plot; Figure S5: Communication and leadership forest plot; Figure S6: Personality characteristics and self-reported health status forest plot; Figure S7: Job attitudes forest plot; Figure S8: Work-family interface forest plot; Figure S9: Perceived intermediate work consequences forest plot; Figure S10: Cumulative meta-analysis of the association between Job demands and occupational burnout by increasing follow-up lengths; Figure S11: Cumulative meta-analysis of the association between Job control and occupational burnout by increasing follow-up lengths; Figure S12: Cumulative meta-analysis of the association between Job resources and occupational burnout by increasing follow-up lengths; Figure S13: Cumulative meta-analysis of the association between Interactions at work and occupational burnout by increasing follow-up lengths; Figure S14: Cumulative meta-analysis of the association between Communication and leadership and occupational burnout by increasing follow-up lengths; Figure S15: Cumulative meta-analysis of the association between Personality characteristics and self-reported health status and occupational burnout by increasing follow-up lengths; Figure S16: Cumulative meta-analysis of the association between Job attitudes and occupational burnout by increasing follow-up lengths; Figure S17: Cumulative meta-analysis of the association between Work-life interface and occupational burnout by increasing follow-up lengths; Figure S18: Cumulative meta-analysis of the association between Perceived intermediate work consequences and occupational burnout by increasing follow-up lengths; Table S2: Results of the sensitivity analysis if deleting one study changes the summary estimate of associations between subgroups of predictor-variables and exhaustion, and the heterogeneity of these summary estimates; Table S3: Comparison of Meta-analysis results per subgroups of studied predictor-variables between overall studies and studies conducted in Europe; Table S4: Comparison of Meta-analysis results per subgroups of studied predictor-variables between overall studies and studies conducted for medical and health workers Table S5: Results of the Regression-based Egger test for small-study effects per subfamily; Figure S19: Job demands funnel plot; Figure S20: Job control funnel plot; Figure S21: Job resources funnel plot; Figure S22: Interactions at work funnel plot; Figure S23: Communication and leadership funnel plot; Figure S24: Personality characteristics and self-reported health status funnel plot; Figure S25: Job attitudes funnel plot; Figure S26: Work-family interface funnel plot; Figure S27: Perceived intermediate work consequences funnel plot. References [51,52,54,55,56,86,87,88,89,90,91,92,93,94,95,96,97,98,99,100,101,102,103,104,105,106,107,108,109,110,111,112,113,114,115,116,117,118,119,120,121,122,123,124,125,126,127,128,129,130,131,132,133,134,135,136,137,138,139,140,141,142,143,144,145] are cited in the supplementary materials.
