# Supplementary material for: Holistic Assessment of Factors Associated with Exhaustion, the Main Symptom of Burnout: A Meta-Analysis of Longitudinal Studies
Source: Int J Environ Res Public Health. 2022 Oct 11;19(20):13037. doi: 10.3390/ijerph192013037 (PMC9602979; doi:10.3390/ijerph192013037)
Supplement: Supplementary file 1 [file ijerph-19-13037-s001.zip › ijerph-1815075-supplementary.pdf]

Supplementary Table S1. Description of the reviewed studies

First author, year, country, population, sample size	Exhaustion assessment (measurement tool, cut-off value)	Predictor-variable definition	Predictor-variable assessment (measurement tool)	Number of measurement points	Follow-up length	Correlation coefficient	Risk of bias
(Adriaenssens et al., 2015),Belgium, Emergency room nurses, n= 170	MBI (Dutch version), NR	Job demands (9 items)<br>Job control (8 items)<br>Social support (8 items)<br>Material recourses (3 items)<br>Social harassment (4 items)<br>Reward (8 items)<br>Work agreements (4 items)<br>Personal resources (4 items)	LQWQ-N: Leiden Quality of Work Questionnaire for Nurses	2	18 months	-0.30*<br>-0.27*<br>-0.14<br>-0.25*<br>-0.04<br>-0.37*<br>-0.31*<br>-0.25*	Low
(Angelo & Chambel, 2015), Portugal, firefighters, n=1610	MBI, NR	Conflicts and interpersonal problems<br>Supervisor's social support	Organizational demands scale constructed by the authors. Supervisory social support was measured with four items (e.g. 'My supervisor is concerned about the welfare of those under him') from the Job Content Questionnaire (Karasek, 1985).	2	12 months	0.21*<br>-0.03	High
(Basinska & Gruszczynska, 2019), Poland, civil servants, n=236	OLBI, NR	Positivity	Job-related Affective Well-being Scale (JAWS)	2	4 months	-0.52*	Moderate
(Birkeland et al., 2018), Norway, technical workers, n= 1263	MBI-GS (Norwegian version), NR	Harmonious passion<br>Obsessive passion<br>Support from supervisor<br>Support from colleagues	Passion Scale (Vallerand et al., 2003), 12 items, six measured harmonious passion and six obsessive passion.<br>Support: four questions from the Perceived Organizational Support Questionnaire	3	10 months	-0.33*<br>0.23*<br>-0.27*<br>-0.21*	Low

(Boamah et al., 2017), Canada, new graduate nurses, n=405	MBI-GS, NR	Authentic leadership<br>Structural empowerment<br>Work–life interference<br>Lack of personnel resources	The Authentic Leadership Questionnaire (ALQ) (Walumbwa et al. 2008), a 16-item questionnaire comprised four subscales: Structural empowerment: the Conditions of Work Effectiveness-II (CWEQ-II) (Laschinger et al. 2001). The Work Interference with Personal Life (WIPL) 7 items. (Fisher-McAuley et al. 2003) Lack of personnel resources: A single-item that assesses how often working short-staffed, affects nurses' ability to provide quality patient care	2	16 months	-0.09*<br>-0.18*<br>0.42*<br>0.26*	Low
(Childs & Stoeber, 2012), UK, healthcare service provision employees, n=69	MBI-GS, NR	Self-oriented perfectionism<br>Socially prescribed perfectionism	Self-oriented perfectionism: the 15-item scale.<br>Socially prescribed perfectionism: 15-item scale from the Multidimensional Perfectionism Scale (MPS; Hewitt & Flett, 1991, 2004).	2	6 months	0.08<br>0.52*	Moderate
(Childs & Stoeber, 2012), UK, school teachers, n=195	MBI-GS, NR	Self-oriented perfectionism<br>Socially prescribed perfectionism	Self-oriented perfectionism: the 15-item scale.<br>Socially prescribed perfectionism: 15-item scale from the Multidimensional Perfectionism Scale (MPS; Hewitt & Flett, 1991, 2004).	2	3 months	0.22*<br>0.43*	Moderate
(Chrisopoulos et al., 2010), Australia, police officers, n=179	MBI-GS, NR	Cognitive demands<br>Emotional demands<br>Physical demands<br>Cognitive resources<br>Physical resources<br>Emotional support	Demand-Induced Strain Questionnaire (DISQ): each of the tasks rated on a five-point scale (1=very rarely/never, 5= very often/always).	2	12 months	0.32*<br>0.36*<br>0.28*<br>-0.47*<br>-0.42*<br>-0.45*	Moderate
(Dubois et al., 2014), Canada, health and social service employees, n=96	MBI, NR	Autonomy<br>Skill discretion<br>Decision authority<br>Support from supervisor<br>Support from colleagues	Autonomy was assessed using three items from the Job Descriptive Scale. Decision latitude and skill discretion were measured with the corresponding subscales of the Conditions of Work Effectiveness	2	12 months	-0.23*<br>-0.09<br>0.15<br>-0.22*<br>-0.06	Moderate

			Questionnaire-II. Support from colleagues was measured with the three-item scale from Podsakoff and MacKenzie, and supervisor support with the six items used by Stinglhamber and Vandenberghe.				
(Fernet et al., 2010), Canada, college employees, n=276	MBI (French-Canadian version), NR	Quality of social interactions at work<br>Self-determined work motivation	The short version of the Blais Work Motivation Inventory.<br>4-item Quality of Interpersonal Relationships Scale.	2	24 months	-0.34*<br>-0.13*	Moderate
(Fernet et al., 2014), Canada, teachers, n=175	MBI (French-Canadian version), NR	Harmonious passion<br>Obsessive passion<br>Autonomy	Passion for teaching: the short form (Lafrenière et al., 2013) of the Passion Scale (Vallerand et al., 2003). Two scales were used to assess autonomy: the three-item subscale developed by Cammann, Fichman, Jenkins, and Klesh (1979), and the control subscale of the Areas of Worklife Scale (Leiter & Maslach, 2000).	2	12 months	-0.31*<br>0.43*<br>-0.32*	Moderate
(Feuerhahn et al., 2013), Germany, teachers, n=56	MBI-ES (German version), NR	Conflicts and interpersonal problems<br>Emotional dissonance<br>Emotional support<br>Classroom disruption<br>Time pressure<br>Self-efficacy	The teacher-specific measure Fragebogen zur Arbeitssituation an Schulen (Kaempf & Krause, 2004). Frankfurt Emotion Work Scales (Zapf et al., 1999). Four items of the Instrument zur stressbezogenen Tätigkeitsanalyse (ISTA) developed by Semmer, Zapf, and Dunckel (1999). Seven items from the teacher-specific measure FASS developed by Krause and colleagues (Kaempf & Krause, 2004). A self-efficacy scale developed specifically for teachers by Schmitz and Schwarzer (2000). The social support questionnaire Fragebogen zur Sozialen Unterstützung (F-SozU; Fydrich, Sommer, & Brähler, 2002).	2	21 months	0.61*<br>0.58*<br>-0.40*<br>0.64*<br>0.33*<br>-0.53*	Low

(Fida et al., 2018), Canada, nurses, n=596	MBI-GS, NR	Work incivility from supervisor<br>Work incivility from coworker<br>Work incivility from physician<br>Relational self-efficacy	Incivility: the Straightforward Incivility Scale (Leiter & Day, 2013). Self-efficacy: the relational subscale of the Occupational Coping Self-Efficacy Questionnaire for Nurses (Pisanti et al., 2008)	2	12 months	0.15*<br>0.27*<br>0.27*<br>-0.20*	Low
(Figueiredo-Ferraz et al., 2012), Spain, nursing professionals, n=316	MBI (Spanish version), NR	Work satisfaction	"Satisfaction Questionnaire S20/23" (Melia and Peiró, 1989): 11 items.	2	12 months	-0.45*	High
(Firoozabadi et al., 2018), Iran, health and social welfare employees, n=123	OLBI, NR	Affective rumination<br>Problem solving<br>Work pressure<br>Work control	Work-Related Rumination Questionnaire (Cropley, Michalianou, Pravettoni, & Millward, 2012). Work pressure: the 13-item Work Pressure subscale of the Tilburg Work Pressure Questionnaire (Roe & Zijlstra, 2000). Work control: the Job Control Questionnaire developed by Greenberger, Strasser, Cummings, and Dunham (1989).	3	18 months	0.31*<br>0.03<br>0.29*<br>-0.23*	Moderate
(Gelsema et al., 2006), the Netherlands, nurses, n=381	MBI (Dutch version), NR	Work and time demands<br>Physical demands<br>Nurse-doctor collaboration<br>Material resources<br>Personal resources communication / information flow<br>Work agreements<br>Reward	LQWLQ-N: from 1 (totally disagree) to 4 (totally agree)	2	36 months	0.32*<br>0.24*<br>-0.14*<br>-0.17*<br>-0.2*<br>-0.2*<br>-0.22*<br>-0.14*	Low
(Gil-Monte & Garcia-Juesas, 2008), Spain, nurses, n=316	MBI, NR	Work and time demands<br>Self-efficacy	Work and time demands: the Spanish adaption of the Karasek scale (7 items), the sclae ranging from 1 "Very often" and 5 "Very rarely."<br>Self-efficacy: the Spanish adaptation of the Baessler scale and Schwarzer (9 items). The scale was completed through a 5-degree frequency format in which 1 "Strongly disagree" and 5 "Strongly agree".	2	12 months	0.42*<br>-0.21*	Low

(Goddard et al., 2006), Australia, teachers, n=316	MBI, NR	Workplace innovation<br>Time pressure<br>Neuroticism	Work Environment Scale (WES; Moos, 1994) modified for teacher respondents according to Fisher and Fraser (1983, 1991). The 12-item short version of the neuroticism subscale of the revised Eysenck Personality Questionnaire (EPQ-R/s; Eysenck & Eysenck, 1991).	4	21 months	0.68*<br>0.73*<br>0.56*	High
(Gonzalez-Morales et al., 2010), Spain, teachers, n=555	MBI-GS (Spanish version), NR	Social support coping<br>Direct action coping<br>Teaching stressors	For the coping strategies, two different dimensions were built from the original Occupational Stress Indicator (OSI) [Cooper, Sloan, & Williams, 1988]. Dimensions. Teaching stressors were measured by means of an experimental scale built from a previous qualitative study in which various focus groups were undertaken to identify the sources of stress experienced by teachers (Peiro´, Rodrı´guez, & Bravo, 2003). This scale was composed of 15 items.	2	6-9 months	0.06<br>-0.18*<br>0.36*	Low
(Gonzalez-Morales et al., 2012), Spain, teachers, n=555	MBI-GS (Spanish version), NR	Colleagues/team exhaustion<br>Workload stressors	The measure for team exhaustion was built following the referent-shift consensus model (Chan, 1998). The items of this measure are the result of the transformation of the MBI-GS burnout measure from an individual referent to a group-level. According to Lyne, Barrett, Williams, and Coaley's (2000) psychometric evaluation of the Occupational Stress Indicator (OSI; Cooper, Sloan, & Williams, 1988) and the revised score key they propose, the 61 items of the ''Sources of Pressure'' scale can be	2	6 months	0.44*<br>0.19*	Low

			scored in three dimensions: managerial pressures, employee pressures, and workload.				
(Gregersen et al., 2014), Germany, healthcare employees, n=339	MBI (German version), NR	Transformational leadership<br>Occupational self-efficacy<br>Perceived strain	Occupational self-efficacy was assessed by a short version of the scale by Rigotti et al. (2008). Transformational leadership: a version of the MLQ5X-Short by Bass and Avolio (1995), as translated and modified by Felfe (2006). Psychological strain was measured using the irritation scale defi ned by Mohr (Mohr, Müller, & Rigotti, 2005 ;Mohr, Müller, Rigotti, Zeynep, & Tschan, 2006 ; Mohr, Rigotti, & Müller, 2005).	2	12 months	-0.29*<br>-0.26<br>0.48	Moderate
(Hochwalder, 2008), Sweden, health care employees, n = 838	MBI (Swedish version), NR	Psychological empowerment	Psychometrically evaluated Swedish translation of Spreitzer's empowerment scale.	2	12 months	-0.32*	Low
(Hornung et al., 2013), Germany, physicians, n=95	MBI (German version), NR	Workload<br>Stressful interactions with patients<br>Work-family conflict	Work overload and patient demands were assessed with two 3-item scales by Bssing and Glaser. Work-family conflict was measured with Netemeyer et al.'s (1996) 5-item scale. All three scales used a 5-point response format (1 = Not at all to 5 = To a very great extent).	2	12 months	0.26*<br>0.27*<br>0.46*	Moderate
(Huang et al., 2012), Taiwan, customs office employees, n=299	MBI-GS, NR	Work and time demands<br>Job control	The measures of job demands and job control that we used were developed and validated by Van Veldhoven (1996) in his dissertation research. A four-point response scale (1 : 'never', 2 : 'sometimes', 3: 'often', 4 : 'always')	2	6 months	0.37*<br>-0.27*	Low

(Hudek-Knezevic et al., 2011), Croatia, female hospital nurses, n= 118	MBI (Croatian version), NR	Extraversion<br>Agreeableness<br>Conscientiousness<br>Neuroticism<br>Openness to experience<br>Organizational commitment<br>Job demands (role conflict and workload)<br>Continuance commitment	Personality measure. Big Five Inventory (BFI). Perceived Organizational Stress Inventory was developed by combining items of several well-known questionnaires measuring work overload, role conflict, and role ambiguity as predictors of stress at work. It consist of 15 items on a five-point scale.. The organizational commitment questionnaire was translated and adapted to Croatian language. It consists of 18 items measuring different aspects of commitment to work organization. Answers are scored on a 5-point scale (1 – definitely disagree; 5 – strongly agree).	2	48 months	0.13<br>-0.15<br>-0.01<br>0.13<br>-0.23*<br>0.43*<br>0.00	Moderate
(Idris et al., 2014), Malaya, private sector employees, n=117	MBI (Malay version), NR	Emotional demands<br>Managment commitment & communication	The Copenhagen Psychosocial Questionnaire (COPSOQ; Kristensen, Hannerz, Hogh, & Borg, 2005). The 12-item PSC scale (PSC-12) (Hall, Dollard, & Coward, 2010).	2	3 months	0.25*<br>-0.21*	Moderate
(Innstrand et al., 2008), Norway, church ministers, n=308	OLBI (Norwegian version), NR	Work-home conflict<br>Work-home facilitation<br>Family-work conflict<br>Family-work facilitation	Work family interaction (WFI) was assessed using a four-dimensional scale from a study by Wayne, Musisca, and Fleeson (2004). The instrument was translated into Norwegian by one of the authors.	2	24 months	0.38*<br>-0.16*<br>0.18*<br>-0.07*	High
(Innstrand et al., 2011), Norway, various working populations, n=2235	OLBI (Norwegian version), NR	Performance-based self-esteem<br>Autonomy<br>goal orientation<br>Workload<br>Value congruency<br>Work-family conflict<br>Work-family facilitation<br>Family-work conflict<br>Family-work facilitation	Job performance–based self-esteem (3 items), goal-orientation (4 items), and value congruency (4 items) were developped for this study and the items were scored on a five-point scale ranging from totally disagree (1) to totally agree (5).<br>Workload (3 items) and autonomy (4 items) scale has been used in a previous studies conducted by the Research Institute of the Norwegian Medical Association (i.e.	2	24 months	0.21*<br>-0.28*<br>-0.13*<br>-0.28*<br>0.36*<br>0.37*<br>-0.04<br>0.21*<br>0.00	High

			Aasland, Olff, Falkum, Schweder, & Ursin, 1997). Work–home interaction was measured by a four-dimensional scale on work–home interaction based on the study by Wayne et al. (2004) and adapted to Norwegian conditions (Innstrand, Langballe, Falkum, Espnes, & Aasland, 2009).				
(Jensen & Knudsen, 2016), Norway, oil and gas company employees, n=1702	MBI-GS (Norwegian version), NR	Psychological health complaints<br>Work-family conflict	The scale measuring psychological health complaints comprised seven questions from the Subjective Health Complaint Inventory. Five items developed by Netemeyer, Boles, and McMurrian (1996) measured Wrok-home conflict.	2	24 months	0.44*<br>0.35*	Moderate
(Jimenez & Dunkl, 2017), Austria, various working populations, n=141	MBI-GS (German version), NR	Value congruency<br>Workload<br>Quality of social interactions at work<br>Job control<br>Job resources<br>Fairness<br>Reward	AWS (6 key dimensions)<br>RESTQ-Work (27 items)	2	6 months	-0.38*<br>0.58*<br>-0.24*<br>-0.37*<br>-0.47*<br>-0.38*<br>-0.47*	Moderate
(Knoll et al., 2019), Germany, various working populations, n= 629	MBI (German version), NR	Acquiescent Silence<br>Quiescent Silence<br>Prosocial Silence<br>Opportunistic Silence	Employee silence associated with four different motivations was measured with Knoll and van Dick's (2013) 12-item scale, which was originally developed in German.	4	4.5 months	0.22*<br>0.26*<br>0.01<br>0.13	High
(Koch & Adler, 2018), Germany, various working populations, n=320	MBI, NR	Task variety<br>Support from supervisor<br>Qualitative overload<br>Unreasonable tasks<br>Individual innovation	Task variety: the Work Design Questionnaire (WDQ). Support from supervisor and qualitative overload<br>were measured using scales from the Salutogenetic Subjective Work Analysis (SALSA).<br>Unreasonable tasks: the Bern Illegitimate Tasks Scale (BITS).<br>Innovation was measured using a nine item	2	12 months	-0.05<br>-0.31*<br>0.42*<br>0.47*<br>0.02	Low

			scale that consists of three facets: idea generation, idea promotion, and idea realization.				
(Konze et al., 2017), Germany, energy supplying employees, n=139	MBI (German version), NR	Work and time demands<br>Emotional dissonance<br>Job control	Work and time demands (3 itwms based on the Short Questionnaire for Job Analysis by Prümber and colleagues). Emotional dissonance (5 items from the Frankfurt Emotion Work Scale). Job control was measured by combining 3 items from the timing control and method control (4 items) subscales developed by Jackson and colleagues.	2	6 months	0.32*<br>0.49*<br>-0.25*	Moderate
(Korunka et al., 2015), Austria, eldercare workers, n=587	MBI (German version), NR	Work and time demands<br>Cognitive demands	Each type of the demands was measured with three items adapted from Ulferts et al. The respondents had to indicate if the respective demand had decreased (1: decreased strongly) or increased (5: increased strongly) in recent years.	2	15 months	0.23*<br>0.09*	Moderate
(Kubicek et al., 2014), Austria, employees in nursing homes for elderly people, n=591	MBI (German version), NR	Working hours<br>Time pressure<br>Job control	Job control: the German self-report instrument for work analysis in hospitals (TAA; Bussing and Glaser, 2002). Time pressure: the respective subscale from the German self-report instrument for work analysis in hospitals (TAA; Bussing and Glaser, 2002).	2	16 months	0.08<br>0.31*<br>-0.14*	Moderate
(Kubicek & Korunka, 2015), Austria, employees in nursing homes	MBI (German version), NR	Cognitive demands<br>Emotional dissonance	Cognitive demands: 4-item subscale of Büssing and Glaser's Emotional dissonance: 5-item subscale of the Frankfurt Emotion Work Scales	2	16 months	0.0<br>0.30*	Moderate

or outpatient care organizations, n=559							
(Langballe et al., 2011), Norway, physicians, n=523	OLBI (Norwegian version), NR	Work-home conflict<br>Work-home facilitation<br>Family-work conflict<br>Family-work facilitation<br>Performance-based self-esteem<br>Autonomy<br>goal orientation<br>Workload<br>Value congruency	JPBSE, GO and VC items were developed for the present study based on Hallsten's burnout theory. The workload and autonomy scales used in this study were based on the scales used in previous studies conducted by Aasland, Olff, Falkum, Schweder, and Ursin (1997) and Cooper, Rout, and Faragher (1989). Work–home interaction was measured by 12 items based on Wayne et al.'s study (2004).	2	24 months	0.45*<br>-0.15*<br>0.19*<br>-0.1*<br>0.25*<br>-0.23*<br>-0.1*<br>0.38*<br>-0.2*	Moderate
(Lapointe et al., 2013), Canada, various working populations, n=224	MBI-GS, NR	Psychological contract breach<br>Organizational commitment<br>Commitment to the supervisor	Robinson and Morrison's (2000) 5-item scale was used to measure psychological contract breach. Organizational commitment was measured using Bentein, Vandenberg, Vandenberghe, and Stinglhamber's (2005) version of Meyer, Allen, and Smith's (1993) 6-item scale. Stinglhamber, Bentein, and Vandenberghe's (2002) 6-item scale was used to measure affective commitment to the supervisor.	3	6 months	0.19*<br>-0.25*<br>-0.23*	Low
(Lavigne et al., 2012), Canada, professionals for the Quebec government, n=325	MBI (French version), NR	Harmonious passion<br>Obsessive passion<br>Flow experiences	Vallerand and colleagues' (2003) Passion Scale and the flow experience at work scale (Forest et al., 2005).	2	6 months	-0.36*<br>0.20*<br>-0.40*	High
(Leiter et al., 2013), Finland, forestry workers, n=4396	MBI-GS (Finnish version), NR	Communication / information flow<br>Predictability<br>Decision authority<br>Skill discretion	Predictability, decision authority, skill discretion: Occupational Stress Questionnaire (Elo, Leppänen, Lindström, &Roponen, 1992). Information flow was operationalized by five items (Väänänen, Pahkin, Kalimo, & Buunk, 2004).	2	48 months	-0.18*<br>-0.17*<br>-0.09*<br>-0.05*	High

(Lizano & Barak, 2012), USA, public child welfare workers, n=335	MBI, NR	Work-family conflict<br>Support from supervisor<br>Organizational support<br>Stress	Beatty's (1996) three-item work–family conflict scale. Organizational and supervisory support variables were measured using eight items developed by Eisenberger, Huntington, Hutchison, and Sowa (1986). Stress: Rizzo, House, and Lirtzman's (1970).	3	12 months	0.27*<br>-0.004<br>-0.12<br>0.23*	Moderate
(Lu et al., 2013) , Taiwan, different organizations of diverse industries, n=245	MBI (Chinese version), NR	Presenteeism<br>Avoidance motives<br>Approach motives<br>Self-efficacy<br>Better physical health<br>Better mental health	Two items were developed to access the behavioral frequency of presenteeism, similar to the construct of “sickness presenteeism” (Aronsson et al., 2000; Demerouti et al., 2009). Mental and physical health: Two subscales from Occupational Stress Indicator (OSI-2; Cooper, Sloan, & Williams, 1998; Lu, Tseng, & Cooper, 1999, for the Chinese version) were used to measure employees’ subjective health symptoms. Self-efficacy: the General Self-Efficacy Scale (5 items; Schwarzer, Babler, Kwiatek, Schroder, & Zhang, 1997; Lu, Chang, & Lai, 2011). Neuroticism: the Neuroticism subscale of Big-Five Mini-Marker Set (8 items; Saucier, 1994; Wu, Lu, Ku, & Chang, 2010). Five items of avoidance motives and 4 items of approach motives were finalized and used.	2	2 months	0.07<br>0.13*<br>-0.02<br>--0.16*<br>0.25*<br>-0.27*<br>-0.33*	High
(Makikangas & Kinnunen, 2003), Finland, various working populations, n=457	MBI, NR	Time pressures<br>Lack of control<br>Poor social climate<br>Job insecurity<br>Self-esteem<br>Optimism	Quantitative Workload Inventory: the subjects responded on a five-point scale (1=not at all, 5=very often). Lack of control was assessed through two scales derived by Jackson, Wall, Martin, and Davids. Job insecurity (4 items) (Caplan, Cobb, French, van Harrison, & Pinneau, 1980).	2	12 months	Men: 0.14, Women: 0.41*<br>Men: 0.08,Women: 0.22<br>Men: 0.24*, Women 0.20<br>Men: 0.05, Women 0.19	Low

			Poor organizational climate was measured by a 10-item scale; the scale was a modification of items based on the study 'Quality of Working Life in Finland 1977–1997' (Lehto & Sutela, 1998; see Kinnunen & Natti, 1994; Litwin & Stringer, 1968). Self-esteem was measured by Rosenberg's (1965) 10-item scale and items were rated on a five-point Likert scale (1=totally agree, 5=totally disagree). Optimism was assessed through an abbreviated version of ''The Revised Life Orientation Test'' developed by Scheier, Carver, and Bridges (1994).			Men: -0.27*, Women -0.19<br>Men: -0.34*, Women -0.16	
(Martinez-Inigo & Totterdell, 2016), Spain., primary healthcare professionals, n=233	MBI (Spanish version), NR	Surface acting<br>Deep acting<br>Autonomy<br>Distributive justice<br>Display rule monitoring	The surface acting measure was a five-item scale. Three items were drawn from Brotheridge and Lee's (2003) Emotional Labor Scale (ELS). Deep acting was originally measured with five items, three items from Brotheridge and Lee's (2003) ELS. The Distributive Justice Index developed by Price and Mueller (1986; cited in Moorman, 1991) and two items from Colquitt's (2001) Organizational Justice Scale. Autonomy the Frankfurt Emotion Work's emotion control subscale (Zapf et al., 1999). Organizational monitoring of display rule compliance was measured with one item ("The organization monitors and penalizes non-fulfilment of display rules")	2	6 months	0.31*<br>0.00<br>0.01<br>-0.33*<br>0.08	High
(McManus et al., 2002), UK, doctors, n=365	An abbreviated nine-item version of the MBI, NR	Stress	The 12-item version of the general health questionnaire GHQ (GHQ-12).	2	36 months	0.45*	Moderate

(Petrou et al., 2015), the Netherlands, police officers, n=580	OLBI, NR	Impact of change<br>Willingness to change<br>Self-initiated resources seeking<br>Self-initiated challenges seeking<br>Self-initiated reducing demands	Impact of changes was measured with a single item by Wanberg and Banas (2000) adapted to refer to the situation of the police officers. Job crafting was measured with items by Petrou et al. (2012).<br>Willingness to change was measured with a 4-item scale developed by Metselaar (1997).	2	12 months	0.17*<br>-0.16*<br>-0.23*<br>-0.18*<br>0.25*	Moderate
(Philipp & Schupbach, 2010), Germany, teachers, n=102	MBI (German version), NR	Surface acting<br>Deep acting	The three surface acting items and two deep acting items were taken from the Emotional Labor Scale (ELS, Brotheridge & Lee, 2003) and translated into German.	2	12 months	0.41*<br>0.13	Moderate
(Pisanti et al., 2016), Italy, nurses, n=217	MBI-HSS (Italian version), NR	Work and time demands<br>Social support<br>Decision latitude	LQWLQ-N: from 1 (totally disagree) to 4 (totally agree)	2	14 months	0.08<br>0.02<br>-0.18*	Moderate
(Pomaki et al., 2009), the Netherlands, nurses, n=172	MBI (Dutch version), the cutoff point is 18	Goal self-efficacy<br>Perception of goal attainability<br>Job satisfaction	The Work Goal Processes inventory and new items (described in the original article) had to be developed based on scales from existing questionnaires. Job satisfaction: the Leiden Quality of Work Questionnaire (van der Doef & Maes, 1999)	2	12 months	-0.09<br>0.01<br>-0.23*	High
(Raimo et al., 2018), USA, residents, n=81	The 9-item abbreviated MBI	Emotional distress<br>Workload satisfaction<br>Learning environment satisfaction	The Seelig et al., stress survey is a 28-item questionnaire, which was found by factor analysis to load into 3 domains: emotional distress (11 items), workload satisfaction (8 items), and learning environment satisfaction (9 items).	2	120 months	0.30*<br>-0.15<br>-0.07	Moderate
(Ramarajan et al., 2008), USA, certified nursing assistants, n=108	MBI, NR	Organizational respect	The 5-item scale that was generated directly from a cross-section of organizational members (through the interviews, focus groups, and town hall meetings).	2	16 months	-0.31*	High

(Richter et al., 2015), Sweden, various working populations, n=3378	MBI-GS, NR	Performance-based self-esteem<br>Work-family conflict	A four-item scale by Hallsten et al. (2005) measured performance-based selfesteem. Work-family conflict was measured with a single item measure (‘Do the demands placed on you at work interfere with your home and family life?’).	2	24 months	0.37*<br>0.26*	Low
(Rubio et al., 2015), Spain, Spanish army subjects, n=242	MBI-GS (Spamish version), NR	Work-family conflict<br>Self-efficacy	Work-family conflict: A sub-scale of the Occupational Stress Indicator (OSI) by Cooper, Sloan, and Williams (1988), translated into Spanish. Professional self-efficacy was measured by the Professional Effi-cacy sub-scale of the Spanish version (Gil-Monte, 2002).	2	12 months	0.25*<br>-0.07	Low
(Salanova et al., 2009), Spain, teachers, n=274	MBI-GS, NR	Decision authority<br>Skill discretion<br>Social support<br>Technical obstacles<br>Conflicts and interpersonal problems	A scale was developed for this study	2	6 months	0.05<br>-0.05<br>-0.05<br>0.09<br>0.15*	High
(Spence Laschinger & Finegan, 2008), Canada, nurse managers, n=134	MBI-GS, NR	Effort-reward imbalance<br>Core self-evaluation	The extrinsic effort and the rewards subscale of the Effort Reward Imbalance (ERI) Questionnaire designed to measure components of Siegrist’s model. Judge et al.’s (2003) 12-item Core Self-evaluation Scale (CSES).	2	12 months	0.60*<br>-0.54*	Low
(Taris et al., 2001), the Netherlands, teachers, n=998	MBI (Dutch version), NR	Stressful interaction with students<br>Stressful interaction with colleagues<br>Organisation stressors<br>Perceived inequity from students<br>Perceived inequity from colleagues<br>Perceived inequity from organisation	Stressors: subscales were taken from Kamphuis and Van Poppel (1994) School Health Questionnaire. Inequity: for each of the three exchange relationships, a general question was posed that measured the imbalance of investments and rewards (a variation on the Hatfield single-item equity measure; Hat-field et al., 1985).	2	12 months	0.22*<br>0.11*<br>0.15*<br>0.17*<br>0.05*<br>0.12*	Low

(Taris et al., 2010), the Netherlands, police officers, n=828	MBI-GS, NR	Work and time demands<br>Professional efficacy<br>Job control	Job demands were measured with four items from a Dutch adaptation of the psychological demands scale of the job content questionnaire.<br>Professional efficacy was measured using the six-item professional efficacy scale of the MBI-GS.	2	12 months	0.25*<br>-0.17*<br>-0.08*	Low
(Thompson et al., 2020), USA, various working populations, n=350	MBI, NR	Workplace ostracism<br>Positive mood<br>Psychological distress<br>Family emotional Exhaustion	Workplace ostracism: Ferris, Brown, Berry, and Lian (2008). Positive mood: Thompson, 2007; Watson, Clark, and Tellegen, 1988. Psychological distress: Lambert et al. (1998).<br>Exhaustion: Maslach et al. (1986)	3	4.5 months	0.19*<br>-0.28*<br>0.23*<br>0.24*	Moderate
(Tonjes & Dickhauser, 2009), Germany, teachers, n=96	MBI (German version), NR	Performance-avoidance goal orientation<br>Learning goals orientation	Adopted from Dickhäuser et al., 2007 using five-level rating scale from is not true at all (1) to is true exactly (5).	2	6 months	0.31*<br>-0.15<br>0.19	High
(Travis et al., 2015), USA, urban public child welfare employees, n=362	MBI, NR	Role ambiguity<br>Role conflict<br>Work-family conflict	Role conflict and role ambiguity were measured using Rizzo,House and Lirtzman's (1970) scale items. Beatty's (1996) three-item work–family conflict scale was used to measure conflict between work and family demands.	2	12 months	0.15<br>0.19*<br>0.25*	Moderate
(Turgut et al., 2016), Germany, employees in a company in the automotive industry, n=709	MBI (German version), NR	Change impact<br>Resistence to change	Change impact: five items from a scale by Caldwell, Herold, and Fedor (2004). Organizational support Resistance to change: two subscales of the resistance to change scale (Oreg, 2003).	2	14 months	0.35*<br>0.32*	Moderate

(Van de Ven et al., 2013), Belgium, technology employees, n=711	MBI-GS, NR	Emotional demands<br>Emotional support<br>Emotional support seeking	Emotional job demands and emotional job resources were measured using the DISC Questionnaire.<br>Emotional support seeking was measured with five items derived from the Emotional Support-Seeking subscale in the Proactive Coping Inventory.	2	12 months	0.29*<br>-0.16*<br>-0.10*	Moderate
(van der Ploeg & Kleber, 2003), the Netherlands, paramedics and drivers (ambulance services), n=123	MBI (Dutch version), NR	Physical strain<br>Lack of social support from colleagues<br>Insufficient financial reward<br>Lack of social support from supervisor<br>Emotional demands<br>Lack of job autonomy<br>Communication / information flow	The Questionnaire on the Experience and Assessment of Work (QEAW) was used (van Veldhoven, 1997).	2	12 months	0.35*<br>0.29*<br>0.09<br>0.41*<br>0.27*<br>0.25*<br>0.26*	Moderate
(Vegchel et al., 2004), Sweden, social insurance organisation employees, n=2255	MBI (Swedish version), NR	Work and time demands<br>Emotional demands<br>Job control<br>Social support	Work and time demands: the questions are partly derived from standard measures used in applications of the DC model and the response scale ranged from 1 (always) to 5 (never).<br>Emotional demands. This scale, developed by M. Soderfeldt. The response scale ranged from 1 (not particularly) to 5 (very much).<br>Job control was measured by an eight-item questionnaire with a 5-point response scale ranging from 1 (never) to 5 (very often), based on the work of Harenstam.<br>Social support. Social support was measured with the 7-item questionnaire (5-point response scale ranging from 1 [always] to 5 [never]) from Harenstam.	2	12 months	0.58*<br>0.37*<br>-0.31*<br>-0.4*	Low
(Welp et al., 2016), Switzerland, nurses and physicians, n=493	MBI-HSS (German, French, and Italian versions), NR	Quality of social interactions at work<br>Cognitive-behavioral teamwork	The three-item nurse-physician relationship scale from the nursing work index revised (NWI-R). Cognitive-behavioral teamwork the validated German, French, and Italian translations of	3	6 months	-0.23*<br>-0.16*	Moderate

			the nine-item safety organizing scale.				
(Wirtz et al., 2017), Germany, leaders of different organizations, n=67	MBI, NR	Colleagues/team exhaustion<br>Workload<br>Autonomy<br>Emotional self–efficacy	The three highest loading items of the MBI (cf. Kinnunen, Mäkikangas, Mauno, De Cuyper, & De Witte, 2014). Autonomy: with a four-item scale (Guest, Isaksson, & de Witte, 2010). Workload: a validated five-item scale (Spector & Jex, 1998). Leader emotional self-efficacy the Occupational Emotional Self-efficacy scale (Loeb, Stempel, & Isaksson, 2016).	2	8 months	0.06<br>0.29*<br>-0.17<br>0.10	High
(Zwingmann et al., 2016), Germany, services company, n=76	MBI-GS, NR	Transformational leadership<br>Laissez-faire leadership<br>Decision latitude<br>Work pressure<br>Job insecurity<br>Social support	Transformational and Laissez-faire leadership: selected items of the German adapted version of the Multifactor Leadership Questionnaire (MLQ 5x; Bass & Avolio, 1995; Felfe & Goihl, 2002). Work pressure and decision latitude: Karasek's job demand-control model (Karasek, 1979). Job insecurity: a scale developed by Siegrist et al. (2004) based on the model of effort-reward imbalance (Siegrist, 1996). Social support: the questionnaire of subjective work analysis (SALSA; Rimann & Udris, 1997) based on Antonovski's concept of salutogenesis (Antonovski, 1987).	2	24 months	0.21*<br>0.19*<br>-0.26*<br>0.14<br>0.31*<br>-0.07	Low

**: ststaically significant, NR: not reported; LQWQ-N: Leiden Quality of Work Questionnaire for Nurses, DEWSS: Dentists' Experienced Work Stressors Scales, DEJRS: Dentists' Experienced Job Resources Scale, BFQ: Big Five Questionnaire,* DCS: Demand-Control-Support, *MBI-GS: Maslach Burnout Inventory general survey, LQWLQ-N: The Leiden Quality of Work Life Questionnaire for nurses, MBI-HSS: Maslach Burnout Inventory Human Service Survey, DC model: Job Demand Control model, OLBI: Oldenburg Burnout Inventory, CBI: Copenhagen Burnout Inventory, AWS: Areas of Work life Scale, RESTQ-Work: The Recovery-Stress-Questionnaire for Work, PBSE: Performance-based self-esteem, GO: goal orientation, VC: Value congruency, OBSE: Organizational based self-esteem, BFI: Big Five Inventory, MBI-ES: Maslach Burnout Inventory Educators Survey,* COPSOQ*: The Copenhagen Psychosocial Questionnaire, PSC: Psychosocial safety climate, Practice Environment Scale of the Nursing Work Index PES-NWI, NWI-R: Nursing work index revised, MLQ: Multifactor Leadership Questionnaire, WRCP: Work-related cell phone, ERI: Effort Reward Imbalance, CSES: Core Self-evaluation Scale, NR: not reported, NA: not applicable..*

References

Adriaenssens, J., De Gucht, V., & Maes, S. (2015). Causes and consequences of occupational stress in emergency nurses, a longitudinal study. *J Nurs Manag*, *23*(3), 346-358. https://doi.org/10.1111/jonm.12138

Angelo, R. P., & Chambel, M. J. (2015). The Reciprocal Relationship Between Work Characteristics and Employee Burnout and Engagement: A Longitudinal Study of Firefighters. *Stress and Health*, *31*(2), 106-114. https://doi.org/10.1002/smi.2532

Basinska, B. A., & Gruszczynska, E. (2019). Job-Related Emotions and Job Burnout among Civil Servants: Examining the Shape of the Relationship in Cross-Sectional and Longitudinal Models. *Medycyna Pracy*, *70*(2), 201-211. https://doi.org/10.13075/mp.5893.00750

Birkeland, I. K., Richardsen, A. M., & Dysvik, A. (2018). The Role of Passion and Support Perceptions in Changing Burnout: A Johnson-Neyman Approach. *International Journal of Stress Management*, *25*(2), 163-180. https://doi.org/10.1037/str0000057

Boamah, S. A., Read, E. A., & Laschinger, H. K. S. (2017). Factors influencing new graduate nurse burnout development, job satisfaction and patient care quality: a time-lagged study. *Journal of Advanced Nursing*, *73*(5), 1182-1195. https://doi.org/10.1111/jan.13215

Childs, J. H., & Stoeber, J. (2012). Do you want me to be perfect? Two longitudinal studies on socially prescribed perfectionism, stress and burnout in the workplace. *Work and Stress*, *26*(4), 347-364. https://doi.org/10.1080/02678373.2012.737547

Chrisopoulos, S., Dollard, M. F., Winefield, A. H., & Dormann, C. (2010). Increasing the probability of finding an interaction in work stress research: A two-wave longitudinal test of the triple-match principle. *Journal of Occupational and Organizational Psychology*, *83*(1), 17-37. https://doi.org/10.1348/096317909x474173

Dubois, C. A., Bentein, K., Ben Mansour, J., Gilbert, F., & Bedard, J. L. (2014). Why Some Employees Adopt or Resist Reorganization of Work Practices in Health Care: Associations between Perceived Loss of Resources, Burnout, and Attitudes to Change. *International Journal of Environmental Research and Public Health*, *11*(1), 187-201. https://doi.org/10.3390/ijerph110100187

Fernet, C., Gagne, M., & Austin, S. (2010). When does quality of relationships with coworkers predict burnout over time? The moderating role of work motivation. *Journal of Organizational Behavior*, *31*(8), 1163-1180. https://doi.org/10.1002/job.673

Fernet, C., Lavigne, G. L., Vallerand, R. J., & Austin, S. (2014). Fired up with passion: Investigating how job autonomy and passion predict burnout at career start in teachers. *Work and Stress*, *28*(3), 270-288. https://doi.org/10.1080/02678373.2014.935524

Feuerhahn, N., Bellingrath, S., & Kudielka, B. M. (2013). The Interplay of Matching and Non-Matching Job Demands and Resources on Emotional Exhaustion among Teachers. *Applied Psychology-Health and Well Being*, *5*(2), 171-192. https://doi.org/10.1111/aphw.12002

Fida, R., Laschinger, H. K. S., & Leiter, M. P. (2018). The protective role of self-efficacy against workplace incivility and burnout in nursing: A time-lagged study. *Health Care Manage Rev*, *43*(1), 21-29. https://doi.org/10.1097/HMR.0000000000000126

Figueiredo-Ferraz, H., Grau-Alberola, E., Gil-Monte, P. R., & Garcia-Juesas, J. A. (2012). [Burnout and job satisfaction among nursing professionals]. *Psicothema*, *24*(2), 271-276. https://www.ncbi.nlm.nih.gov/pubmed/22420356 (Sindrome de quemarse por el trabajo y satisfaccion laboral en profesionales de enfermeria.)

Firoozabadi, A., Uitdewilligen, S., & Zijlstra, F. R. H. (2018). Should You Switch Off or Stay Engaged? The Consequences of Thinking About Work on the Trajectory of Psychological Well-Being Over Time. *Journal of Occupational Health Psychology*, *23*(2), 278-288. https://doi.org/10.1037/ocp0000068

Gelsema, T. I., van der Doef, M., Maes, S., Janssen, M., Akerboom, S., & Verhoeven, C. (2006). A longitudinal study of job stress in the nursing profession: causes and consequences. *J Nurs Manag*, *14*(4), 289-299. https://doi.org/10.1111/j.1365-2934.2006.00635.x

Gil-Monte, P. R., & Garcia-Juesas, J. A. (2008). Effects of Work Overloading and Self-Efficacy over the Burnout Syndrome: A Longitudinal Survey About Nursing. *Revista Mexicana De Psicologia*, *25*(2), 329-337. <Go to ISI>://WOS:000261236500012

Goddard, R., O'Brien, P., & Goddard, M. (2006). Work environment predictors of beginning teacher burnout. *British Educational Research Journal*, *32*(6), 857-874. https://doi.org/10.1080/01411920600989511

Gonzalez-Morales, M. G., Peiro, J. M., Rodriguez, I., & Bliese, P. D. (2012). Perceived collective burnout: a multilevel explanation of burnout. *Anxiety Stress and Coping*, *25*(1), 43-61. https://doi.org/10.1080/10615806.2010.542808

Gonzalez-Morales, M. G., Rodriguez, I., & Peiro, J. M. (2010). A Longitudinal Study of Coping and Gender in a Female-Dominated Occupation: Predicting Teachers' Burnout. *Journal of Occupational Health Psychology*, *15*(1), 29-44. https://doi.org/10.1037/a0018232

Gregersen, S., Vincent-Höper, S., & Nienhaus, A. (2014). The Relation Between Leadership and Perceived Well-Being: What Role Does Occupational Self-Efficacy Play? *Journal of Leadership Studies*, *8*. https://doi.org/10.1002/jls.21318

Hochwalder, J. (2008). A longitudinal study of the relationship between empowerment and burnout among registered and assistant nurses. *Work-a Journal of Prevention Assessment & Rehabilitation*, *30*(4), 343-352. <Go to ISI>://WOS:000258695100002

Hornung, S., Weigl, M., Glaser, J., & Angerer, P. (2013). Is It So Bad or Am I So Tired? Cross-Lagged Relationships Between Job Stressors and Emotional Exhaustion of Hospital Physicians. *Journal of Personnel Psychology*, *12*(3), 124-131. https://doi.org/10.1027/1866-5888/a000088

Huang, Y. H., Chen, C. H., Du, P. L., & Huang, I. C. (2012). The causal relationships between job characteristics, burnout, and psychological health: a two-wave panel study. *International Journal of Human Resource Management*, *23*(10), 2108-2125. https://doi.org/10.1080/09585192.2011.610934

Hudek-Knezevic, J., Maglica, B. K., & Krapic, N. (2011). Personality, organizational stress, and attitudes toward work as prospective predictors of professional burnout in hospital nurses. *Croatian Medical Journal*, *52*(4), 538-549. https://doi.org/10.3325/cmj.2011.52.538

Idris, M. A., Dollard, M. F., & Yulita. (2014). Psychosocial safety climate, emotional demands, burnout, and depression: a longitudinal multilevel study in the Malaysian private sector. *J Occup Health Psychol*, *19*(3), 291-302. https://doi.org/10.1037/a0036599

Innstrand, S. T., Langballe, E., Espnes, G., Falkum, & Aasland. (2008). Positive and negative work-family interaction and burnout: A longitudinal study of reciprocal relations. *Work and Stress*, 1-15. https://doi.org/10.1080/02678370801975842

Innstrand, S. T., Langballe, E., & Falkum, E. (2011). The longitudinal effects of individual vulnerability, organisational factors, and work–home interaction on burnout among male church ministers in Norway. *Mental Health, Religion & Culture*, 241-257. https://doi.org/10.1080/13674670903470621

Jensen, M., & Knudsen, K. (2016). A two-wave cross-lagged study of business travel, work–family conflict, emotional exhaustion, and psychological health complaints. *European Journal of Work and Organizational Psychology*, *26*, 1-12. https://doi.org/10.1080/1359432X.2016.1197206

Jimenez, P., & Dunkl, A. (2017). The Buffering Effect of Workplace Resources on the Relationship between the Areas of Worklife and Burnout. *Frontiers in Psychology*, *8*. https://doi.org/ARTN 1210.3389/fpsyg.2017.00012

Knoll, M., Hall, R. J., & Weigelt, O. (2019). A Longitudinal Study of the Relationships Between Four Differentially Motivated Forms of Employee Silence and Burnout. *Journal of Occupational Health Psychology*, *24*(5), 572-589. https://doi.org/10.1037/ocp0000143

Koch, A. K., & Adler, M. (2018). Emotional exhaustion and innovation in the workplace-a longitudinal study. *Ind Health*, *56*(6), 524-538. https://doi.org/10.2486/indhealth.2017-0095

Konze, A.-K., Rivkin, W., & Schmidt, K.-H. (2017). Is Job Control a Double-Edged Sword? A Cross-Lagged Panel Study on the Interplay of QuantitativeWorkload, Emotional Dissonance, and Job Control on Emotional Exhaustion. *International Journal of Environmental Research and Public Health*, *14*. https://doi.org/10.3390/ijerph14121608

Korunka, C., Kubicek, B., Paskvan, M., & Ulferts, H. (2015). Changes in work intensification and intensified learning: challenge or hindrance demands? *Journal of Managerial Psychology*, *30*(7), 786-800. https://doi.org/10.1108/Jmp-02-2013-0065

Kubicek, B., & Korunka, C. (2015). Does job complexity mitigate the negative effect of emotion-rule dissonance on employee burnout? *Work and Stress*, *29*(4), 379-400. https://doi.org/10.1080/02678373.2015.1074954

Kubicek, B., Korunka, C., & Tement, S. (2014). Too much job control? Two studies on curvilinear relations between job control and eldercare workers' well-being. *International journal of nursing studies*, *51*. https://doi.org/10.1016/j.ijnurstu.2014.05.005

Langballe, E., Innstrand, S. T., Aasland, O., & Falkum, E. (2011). The Predictive Value of Individual Factors, Work-Related Factors, and Work-Home Interaction on Burnout in Female and Male Physicians: A Longitudinal Study. *Stress and Health*, *27*, 73-87. https://doi.org/10.1002/smi.1321

Lapointe, É., Vandenberghe, C., & Boudrias, J.-S. (2013). Psychological contract breach, affective commitment to organization and supervisor, and newcomer adjustment: A three-wave moderated mediation model. *Journal of Vocational Behavior*, *83*, 528–538. https://doi.org/10.1016/j.jvb.2013.07.008

Lavigne, G. L., Forest, J., & Crevier-Braud, L. (2012). Passion at work and burnout: A two-study test of the mediating role of flow experiences. *European Journal of Work and Organizational Psychology*, *21*(4), 518-546. https://doi.org/10.1080/1359432x.2011.578390

Leiter, M. P., Hakanen, J. J., Ahola, K., Toppinen-Tanner, S., Koskinen, A., & Vaananen, A. (2013). Organizational predictors and health consequences of changes in burnout: A 12-year cohort study. *Journal of Organizational Behavior*, *34*(7), 959-973. https://doi.org/10.1002/job.1830

Lizano, E. L., & Barak, M. E. M. (2012). Workplace demands and resources as antecedents of job burnout among public child welfare workers: A longitudinal study. *Children and Youth Services Review*, *34*(9), 1769-1776. https://doi.org/10.1016/j.childyouth.2012.02.006

Lu, L., Lin, H. Y., & Cooper, C. L. (2013). Unhealthy and present: motives and consequences of the act of presenteeism among Taiwanese employees. *J Occup Health Psychol*, *18*(4), 406-416. https://doi.org/10.1037/a0034331

Makikangas, A., & Kinnunen, U. (2003). Psychosocial work stressors and well-being: self-esteem and optimism as moderators in a one-year longitudinal sample. *Personality and Individual Differences*, *35*(3), 537-557. Doi 10.1016/S0191-8869(02)00217-9

Martinez-Inigo, D., & Totterdell, P. (2016). The mediating role of distributive justice perceptions in the relationship between emotion regulation and emotional exhaustion in healthcare workers. *Work and Stress*, *30*(1), 26-45. https://doi.org/10.1080/02678373.2015.1126768

McManus, I. C., Winder, B. C., & Gordon, D. (2002). The causal links between stress and burnout in a longitudinal study of UK doctors. *Lancet*, *359*(9323), 2089-2090. https://doi.org/10.1016/s0140-6736(02)08915-8

Petrou, P., Demerouti, E., & Schaufeli, W. B. (2015). Job crafting in changing organizations: Antecedents and implications for exhaustion and performance. *J Occup Health Psychol*, *20*(4), 470-480. https://doi.org/10.1037/a0039003

Philipp, A., & Schupbach, H. (2010). Longitudinal effects of emotional labour on emotional exhaustion and dedication of teachers. *J Occup Health Psychol*, *15*(4), 494-504. https://doi.org/10.1037/a0021046

Pisanti, R., van der Doef, M., Maes, S., Meier, L. L., Lazzari, D., & Violani, C. (2016). How Changes in Psychosocial Job Characteristics Impact Burnout in Nurses: A Longitudinal Analysis. *Frontiers in Psychology*, *7*. https://doi.org/ARTN 108210.3359/fpsyg.2016.01082

Pomaki, G., Karoly, P., & Maes, S. (2009). Linking Goal Progress to Subjective Well-Being at Work: The Moderating Role of Goal-Related Self-Efficacy and Attainability. *Journal of Occupational Health Psychology*, *14*(2), 206-218. https://doi.org/10.1037/a0014605

Raimo, J., LaVine, S., Spielmann, K., Akerman, M., Friedman, K. A., Katona, K., & Chaudhry, S. (2018). The Correlation of Stress in Residency With Future Stress and Burnout: A 10-Year Prospective Cohort Study. *J Grad Med Educ*, *10*(5), 524-531. https://doi.org/10.4300/JGME-D-18-00273.1

Ramarajan, L., Barsade, S., & Burack, O. (2008). The influence of organizational respect on emotional exhaustion in the human services. *The Journal of Positive Psychology*, *3*, 4-18. https://doi.org/10.1080/17439760701750980

Richter, A., Schraml, K., & Leineweber, C. (2015). Work-family conflict, emotional exhaustion and performance-based self-esteem: reciprocal relationships. *Int Arch Occup Environ Health*, *88*(1), 103-112. https://doi.org/10.1007/s00420-014-0941-x

Rubio, C., Osca, A., Recio, P., Urien, B., & Peiro, J. M. (2015). Work-family conflict, self-efficacy, and emotional exhaustion: A test of longitudinal effects. *Journal of Work and Organizational Psychology-Revista De Psicologia Del Trabajo Y De Las Organizaciones*, *31*(3), 147-154. https://doi.org/10.1016/j.rpto.2015.06.004

Salanova, M., Schaufeli, W., Martínez, I., & Bresó, E. (2009). How obstacles and facilitators predict academic performance: The mediating role of study burnout and engagement. *Anxiety, stress, and coping*, *23*, 53-70. https://doi.org/10.1080/10615800802609965

Spence Laschinger, H. K., & Finegan, J. (2008). Situational and dispositional predictors of nurse manager burnout: a time-lagged analysis. *J Nurs Manag*, *16*(5), 601-607. https://doi.org/10.1111/j.1365-2834.2008.00904.x

Taris, T. W., Kompier, M. A. J., Geurts, S. A. E., Houtman, I. L. D., & van den Heuvel, F. F. M. (2010). Professional efficacy, exhaustion, and work characteristics among police officers: A longitudinal test of the learning-related predictions of the demand-control model. *Journal of Occupational and Organizational Psychology*, *83*(2), 455-474. https://doi.org/10.1348/096317909x424583

Taris, T. W., Peeters, M. C., Le Blanc, P. M., Schreurs, P. J., & Schaufeli, W. B. (2001). From inequity to burnout: the role of job stress. *J Occup Health Psychol*, *6*(4), 303-323. https://www.ncbi.nlm.nih.gov/pubmed/11605825

Thompson, M. J., Carlson, D. S., Kacmar, K. M., & Vogel, R. M. (2020). The Cost of Being Ignored: Emotional Exhaustion in the Work and Family Domains. *Journal of Applied Psychology*, *105*(2), 186-195. https://doi.org/10.1037/apl0000433

Tonjes, B., & Dickhauser, O. (2009). Longitudinal effects of goal orientation on factors of occupational burden in the teacher profession. *Zeitschrift Fur Entwicklungspsychologie Und Padagogische Psychologie*, *41*(2), 79-86. https://doi.org/10.1026/0049-8637.41.2.79

Travis, D., Lizano, E., & Mor Barak, M. (2015). 'I'm So Stressed!': A Longitudinal Model of Stress, Burnout and Engagement among Social Workers in Child Welfare Settings. *British Journal of Social Work*, *46*. https://doi.org/10.1093/bjsw/bct205

Turgut, S., Michel, A., Rothenhofer, L. M., & Sonntag, K. (2016). Dispositional resistance to change and emotional exhaustion: moderating effects at the work-unit level. *European Journal of Work and Organizational Psychology*, *25*(5), 735-750. https://doi.org/10.1080/1359432x.2016.1157583

Van de Ven, B., van den Tooren, M., & Vlerick, P. (2013). Emotional Job Resources and Emotional Support Seeking as Moderators of the Relation Between Emotional Job Demands and Emotional Exhaustion: A Two-Wave Panel Study. *Journal of Occupational Health Psychology*, *18*(1), 1-8. https://doi.org/10.1037/a0030656

van der Ploeg, E., & Kleber, R. J. (2003). Acute and chronic job stressors among ambulance personnel: predictors of health symptoms. *Occupational and Environmental Medicine*, *60*, 40-46. <Go to ISI>://WOS:000183251600008

Vegchel, N. v., Jonge, J. d., Söderfeldt, M., Dormann, C., & Schaufeli, W. (2004). Quantitative Versus Emotional Demands Among Swedish Human Service Employees: Moderating Effects of Job Control and Social Support. *International Journal of Stress Management*, *11*(1), 21-40. https://doi.org/10.1037/1072-5245.11.1.21

Welp, A., Meier, L., & Manser, T. (2016). The interplay between teamwork, clinicians' emotional exhaustion, and clinician-rated patient safety: A longitudinal study. *Critical Care*, *20*. https://doi.org/10.1186/s13054-016-1282-9

Wirtz, N., Rigotti, T., Otto, K., & Loeb, C. (2017). What About the Leader? Crossover of Emotional Exhaustion and Work Engagement From Followers to Leaders. *Journal of Occupational Health Psychology*, *22*(1), 86-97. https://doi.org/10.1037/ocp0000024

Zwingmann, I., Wolf, S., & Richter, P. (2016). Every light has its shadow: a longitudinal study of transformational leadership and leaders' emotional exhaustion. *Journal of Applied Social Psychology*, *46*(1), 19-33. https://doi.org/10.1111/jasp.12352

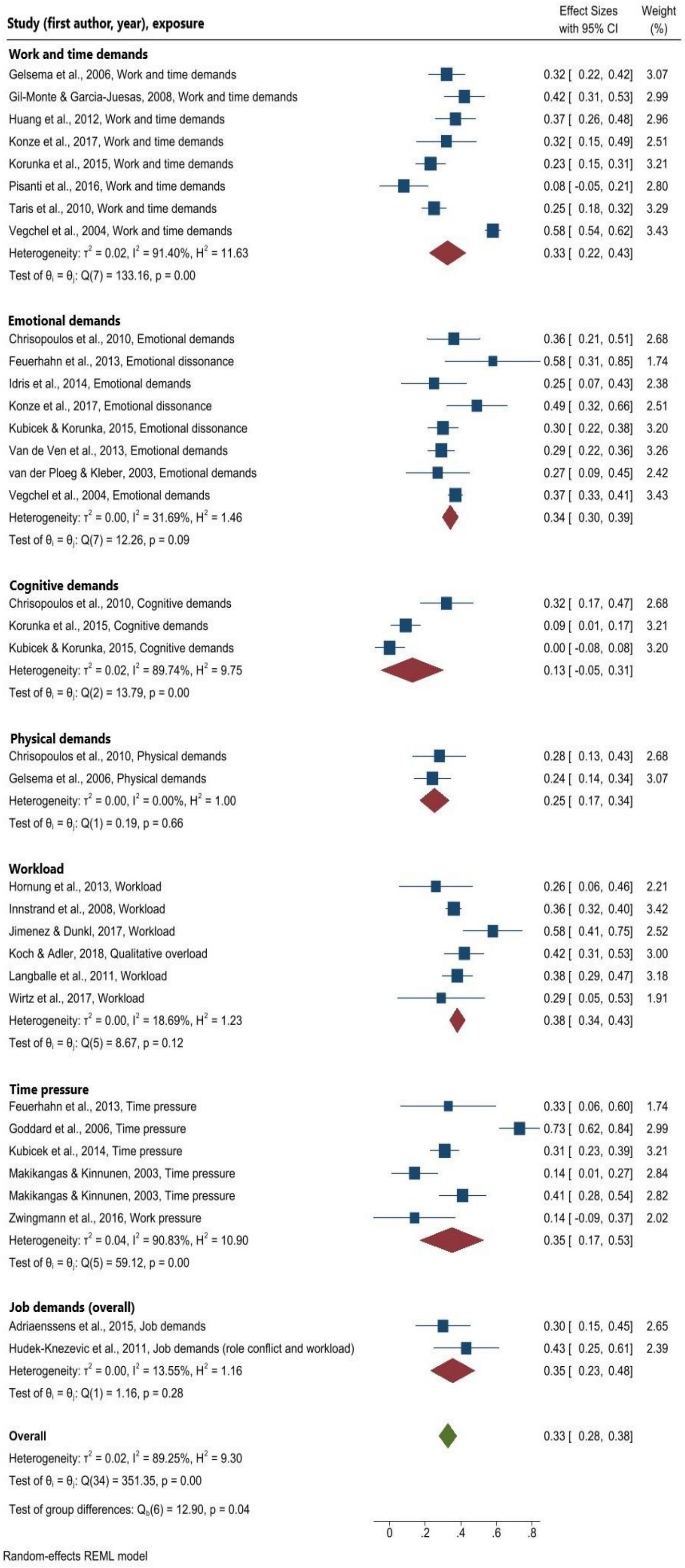

Supplementary Figure S1. Job demands forest plot

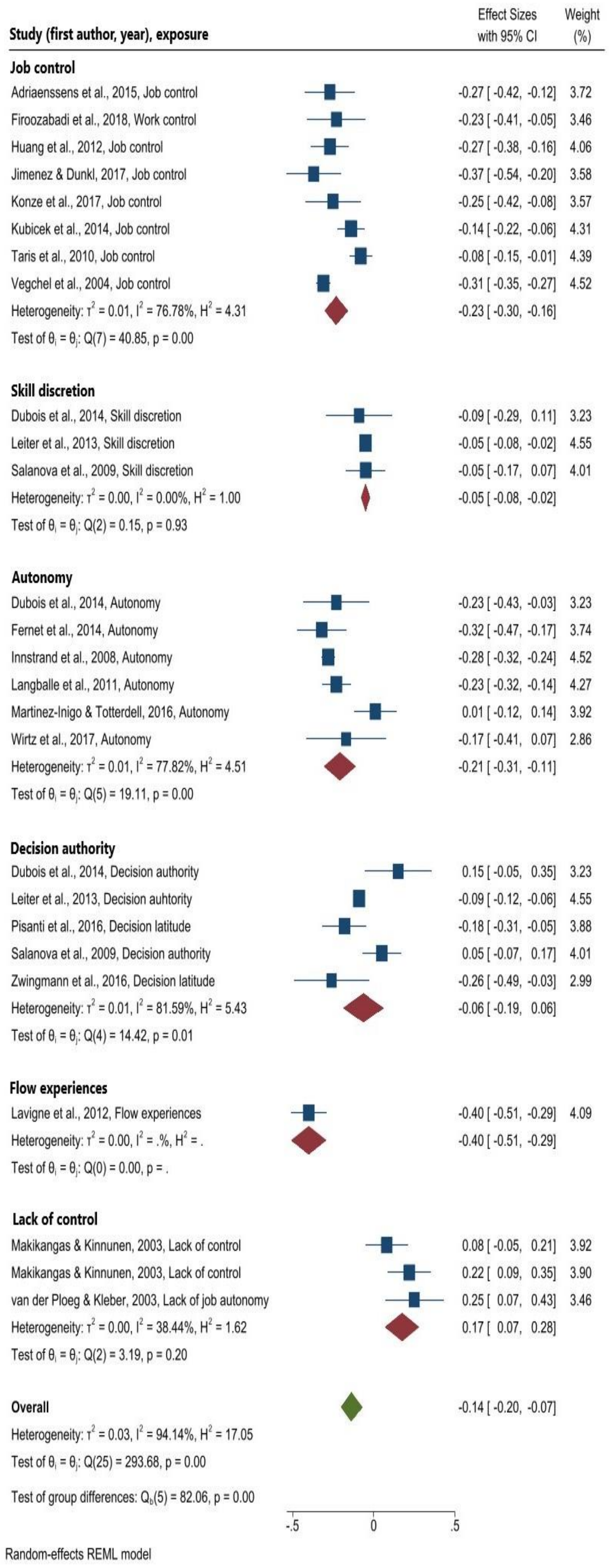

Supplementary Figure S2. Job control forest plot

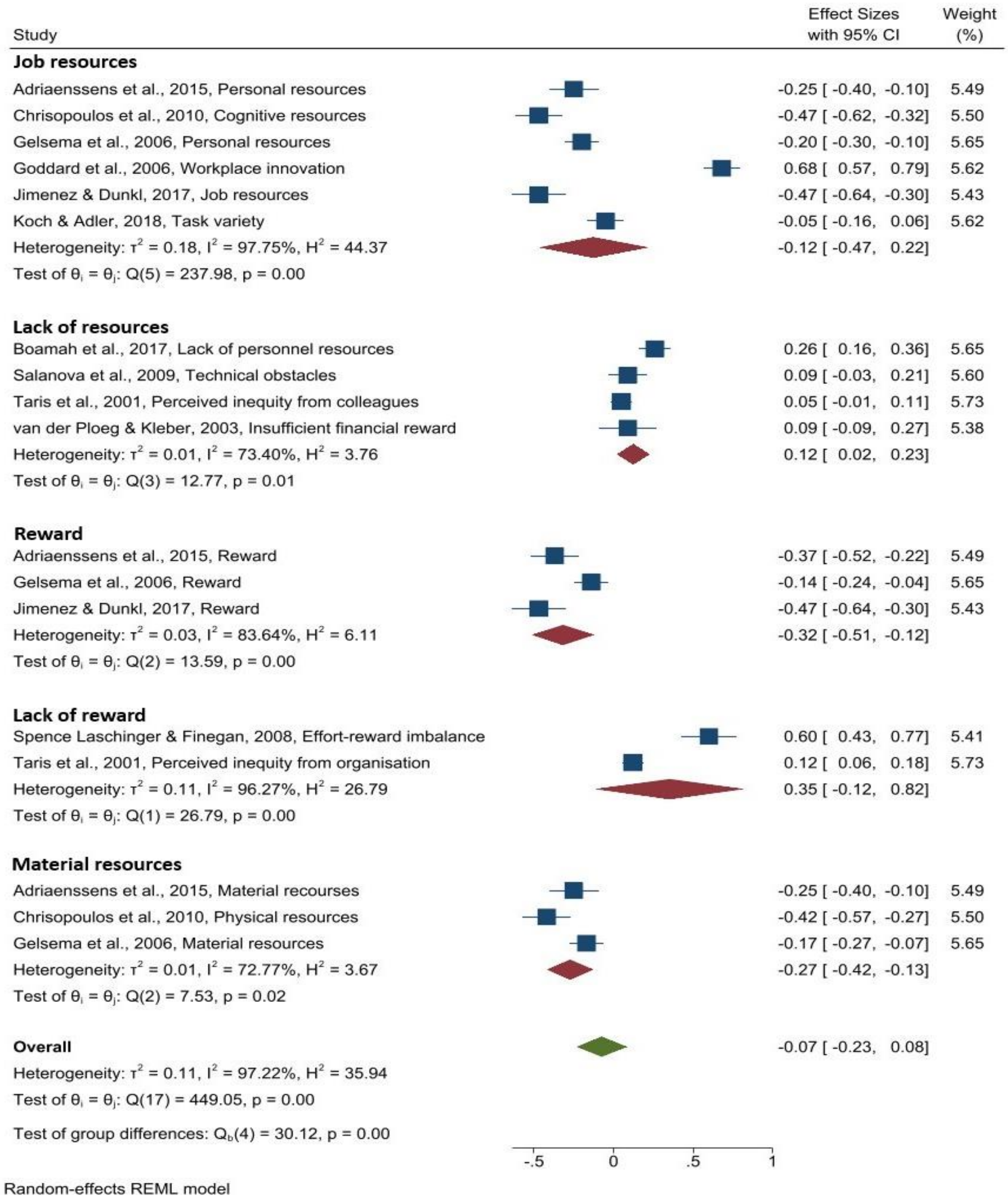

Supplementary Figure S3. Job resources forest plot

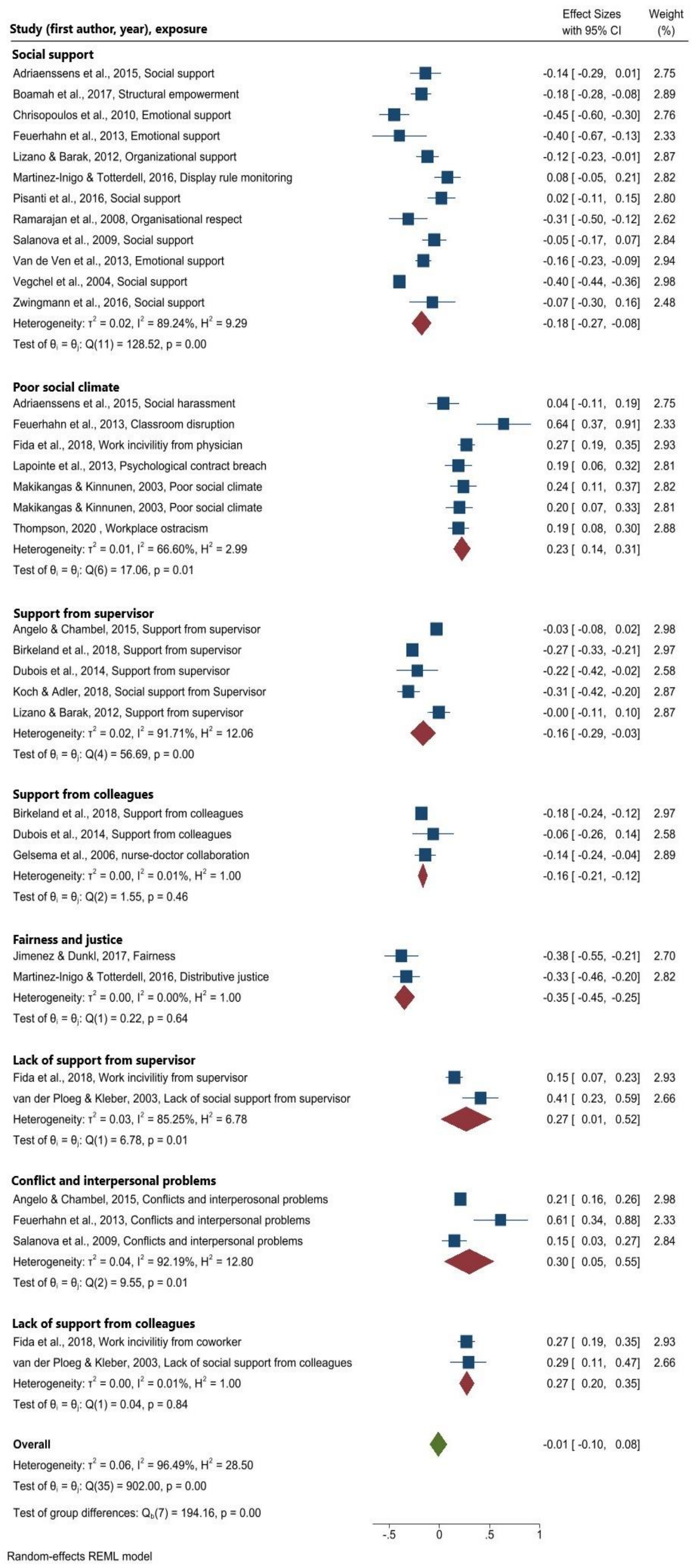

Supplementary Figure S4. Interactions at work forest plot

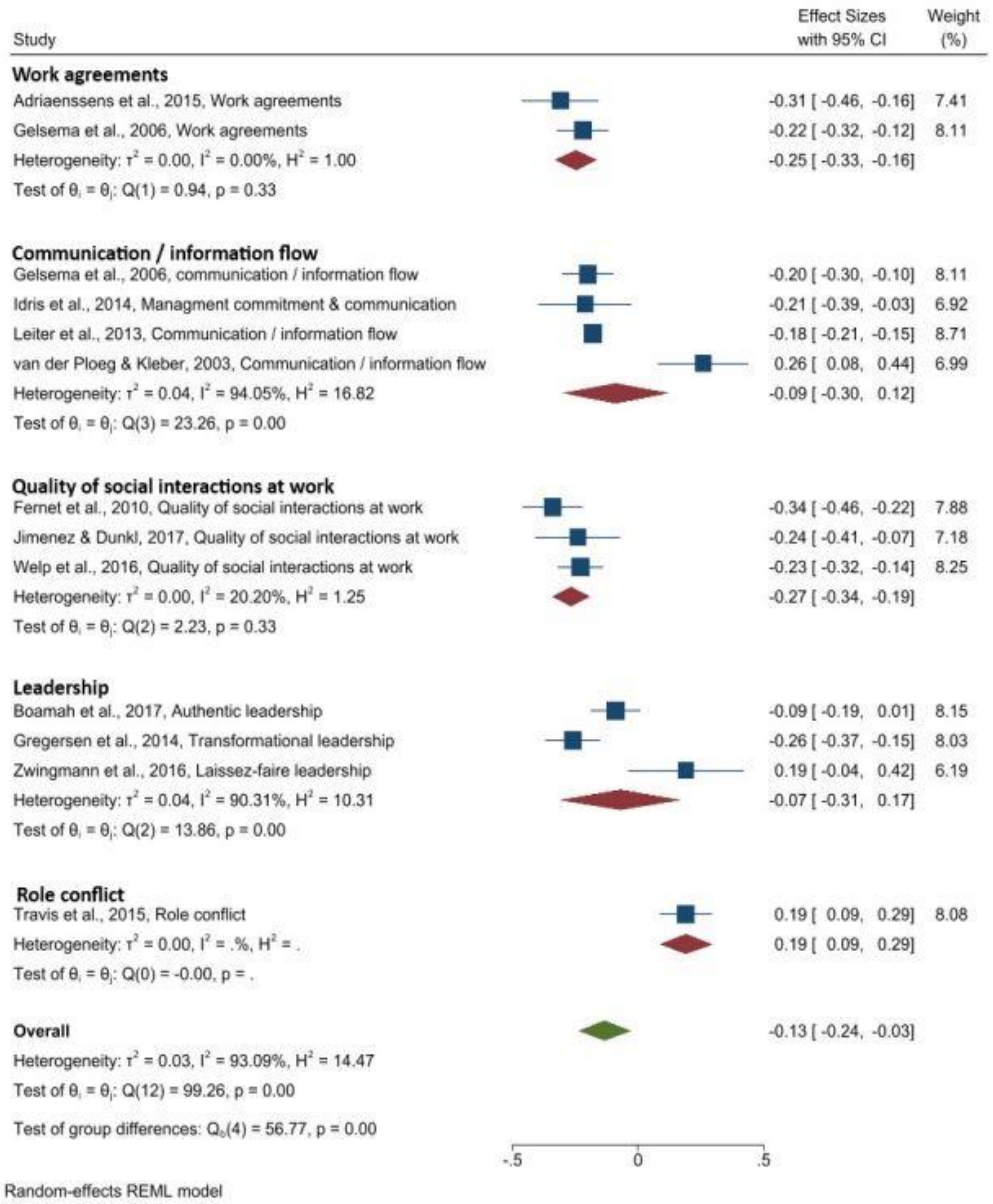

Supplementary Figure S5. Communication and leadership forest plot

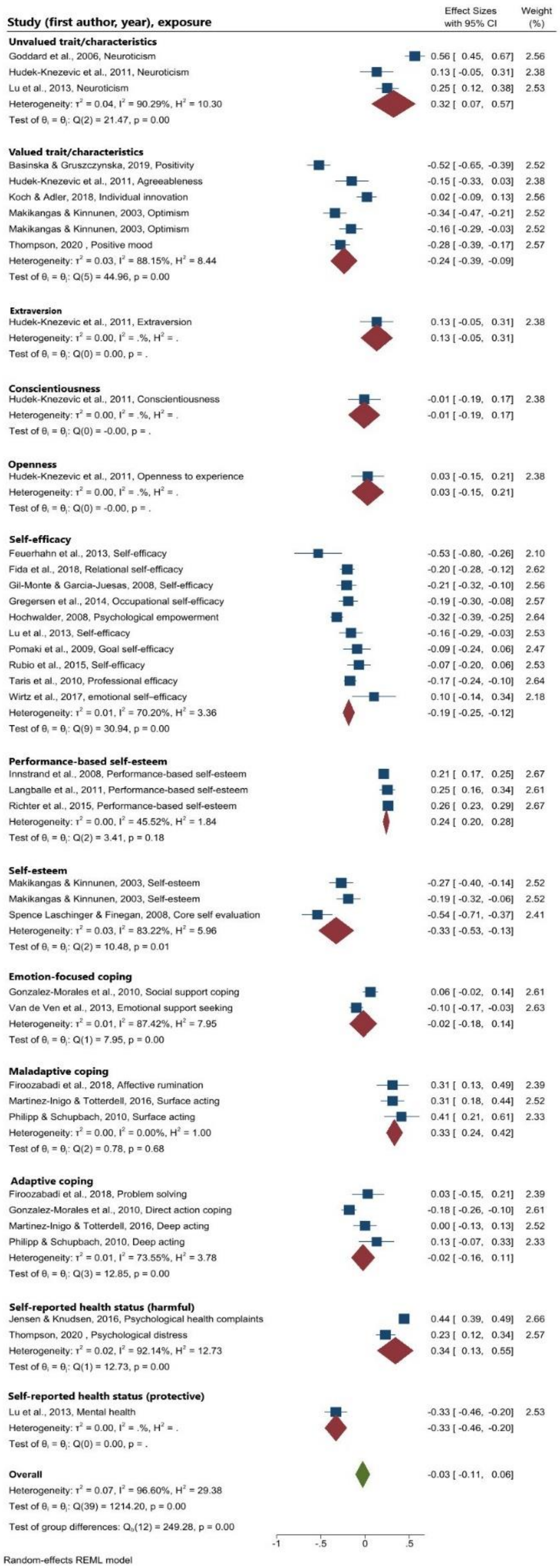

Supplementary Figure S6. Personality characteristics and self-reported health status forest plot

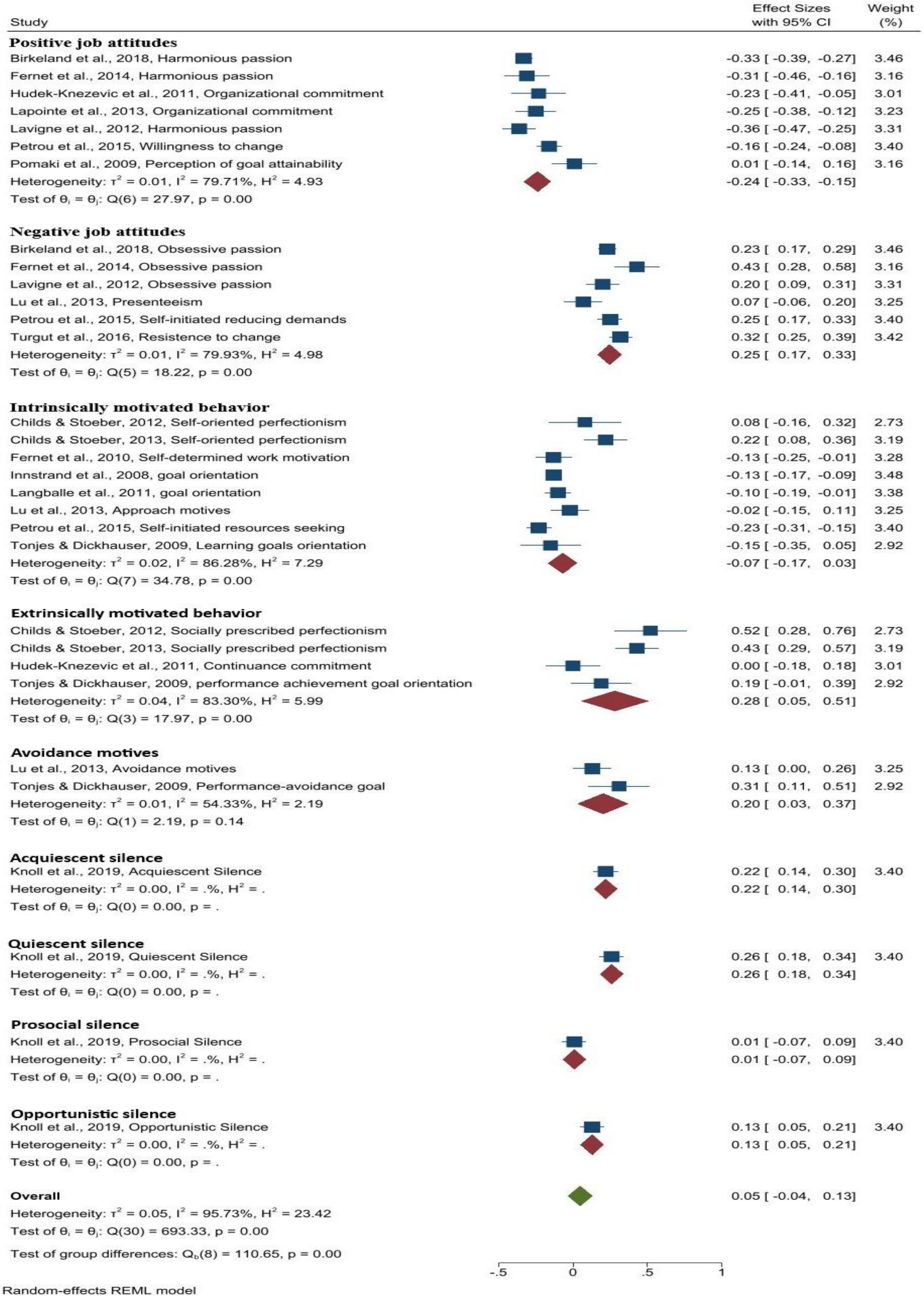

Supplementary Figure S7. Job attitudes forest plot

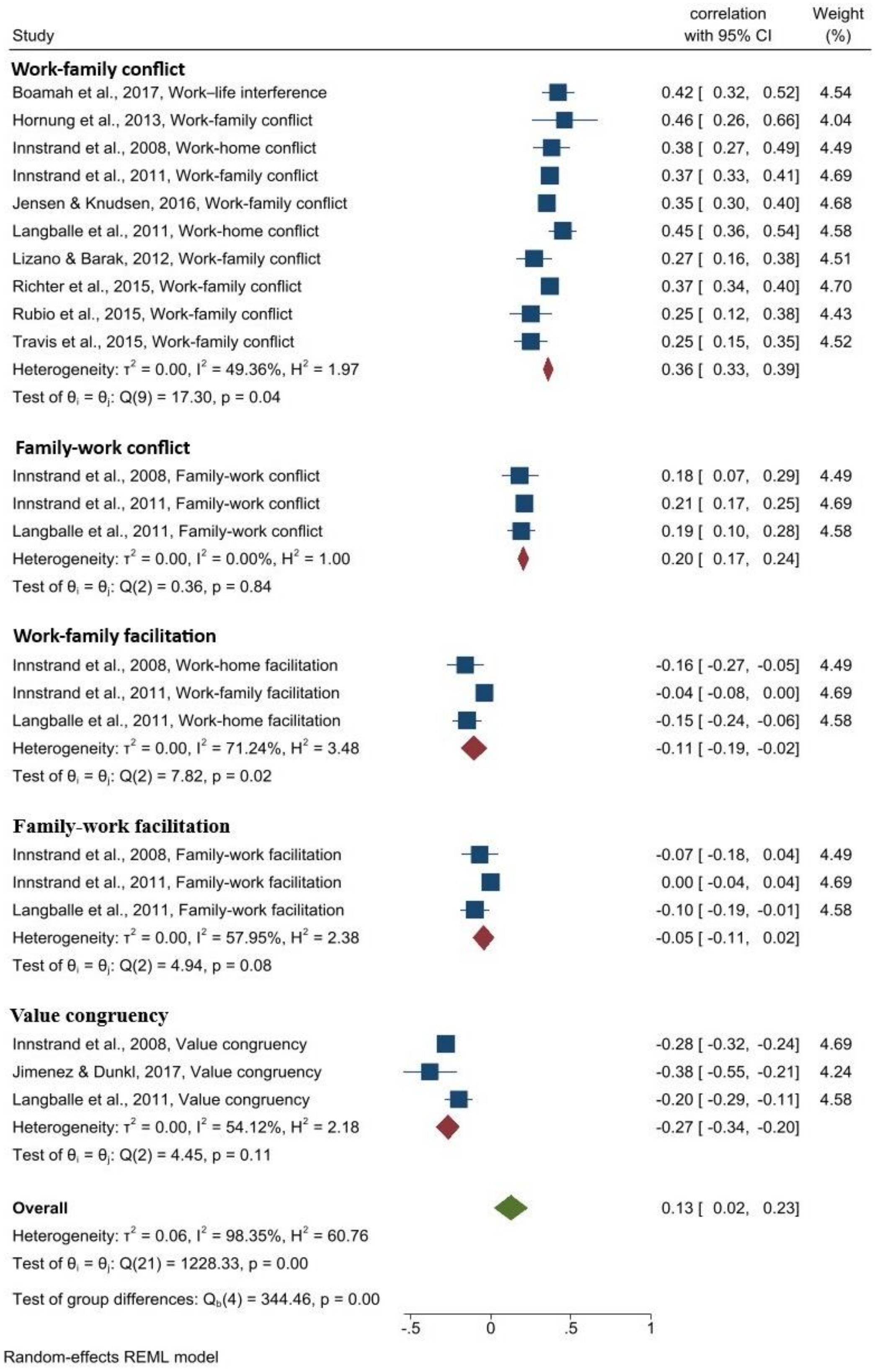

Supplementary Figure S8. Work-family interface forest plot

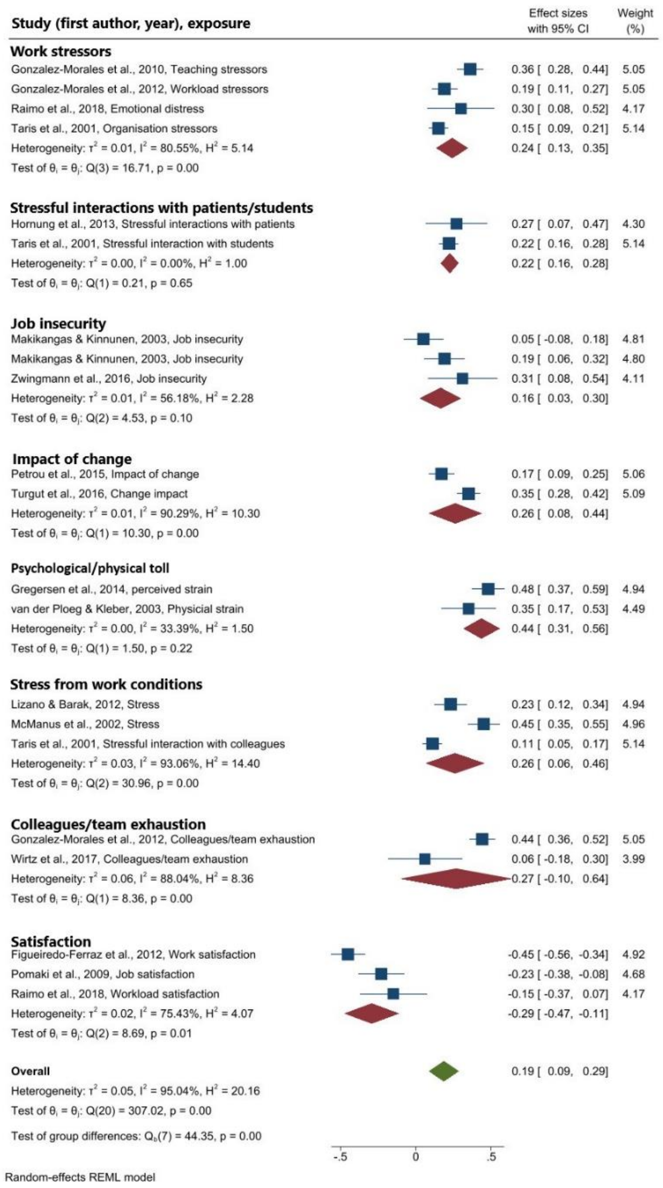

Supplementary Figure S9. Perceived intermediate work consequences forest plot

Study	Effect Sizes with 95% CI	P-value	Follow- up length
Idris et al., 2014, Emotional demands	0.25 [0.07, 0.43]	0.007	3
Huang et al., 2012, Work and time demands	0.33 [0.22, 0.44]	0.000	6
Jimenez & Dunkl, 2017, Workload	0.40 [0.22, 0.58]	0.000	6
Konze et al., 2017, Work and time demands	0.38 [0.25, 0.51]	0.000	6
Konze et al., 2017, Emotional dissonance	0.40 [0.29, 0.51]	0.000	6
Wirtz et al., 2017, Workload	0.39 [0.29, 0.49]	0.000	9
Chrisopoulos et al., 2010, Cognitive demands	0.38 [0.30, 0.46]	0.000	12
Chrisopoulos et al., 2010, Emotional demands	0.38 [0.31, 0.45]	0.000	12
Chrisopoulos et al., 2010, Physical demands	0.37 [0.30, 0.43]	0.000	12
Firoozabadi et al., 2018, Work pressure	0.36 [0.30, 0.42]	0.000	12
Gil-Monte & Garcia-Juesas, 2008, Work and time demands	0.37 [0.31, 0.42]	0.000	12
Hornung et al., 2013, Workload	0.36 [0.31, 0.41]	0.000	12
Koch & Adler, 2018, Qualitative overload	0.37 [0.32, 0.42]	0.000	12
Makikangas & Kinnunen, 2003, Time pressure	0.35 [0.29, 0.41]	0.000	12
Makikangas & Kinnunen, 2003, Time pressure	0.35 [0.30, 0.41]	0.000	12
Taris et al., 2010, Work and time demands	0.34 [0.29, 0.39]	0.000	12
Van de Ven et al., 2013, Emotional demands	0.34 [0.29, 0.39]	0.000	12
van der Ploeg & Kleber, 2003, Emotional demands	0.33 [0.29, 0.38]	0.000	12
Vegchel et al., 2004, Work and time demands	0.35 [0.30, 0.41]	0.000	12
Vegchel et al., 2004, Emotional demands	0.35 [0.30, 0.41]	0.000	12
Adriaenssens et al., 2015, Job demands	0.35 [0.30, 0.40]	0.000	18
Feuerhahn et al., 2013, Emotional dissonance	0.36 [0.31, 0.41]	0.000	24
Feuerhahn et al., 2013, Time pressure	0.36 [0.31, 0.41]	0.000	24
Goddard et al., 2006, Time pressure	0.37 [0.32, 0.43]	0.000	24
Innstrand et al., 2008, Workload	0.37 [0.32, 0.43]	0.000	24
Korunka et al., 2015, Work and time demands	0.37 [0.31, 0.42]	0.000	24
Korunka et al., 2015, Cognitive demands	0.35 [0.30, 0.41]	0.000	24
Kubicek et al., 2014, Time pressure	0.35 [0.30, 0.41]	0.000	24
Kubicek & Korunka, 2015, Cognitive demands	0.34 [0.28, 0.40]	0.000	24
Kubicek & Korunka, 2015, Emotional dissonance	0.34 [0.28, 0.39]	0.000	24
Langballe et al., 2011, Workload	0.34 [0.28, 0.39]	0.000	24
Pisanti et al., 2016, Work and time demands	0.33 [0.28, 0.38]	0.000	24
Zwingmann et al., 2016, Work pressure	0.33 [0.27, 0.38]	0.000	24
Gelsema et al., 2006, Work and time demands	0.33 [0.27, 0.38]	0.000	36
Gelsema et al., 2006, Physical demands	0.32 [0.27, 0.37]	0.000	36
Hudek-Knezevic et al., 2011, Job demands (role conflict and workload)	0.33 [0.28, 0.38]	0.000	48

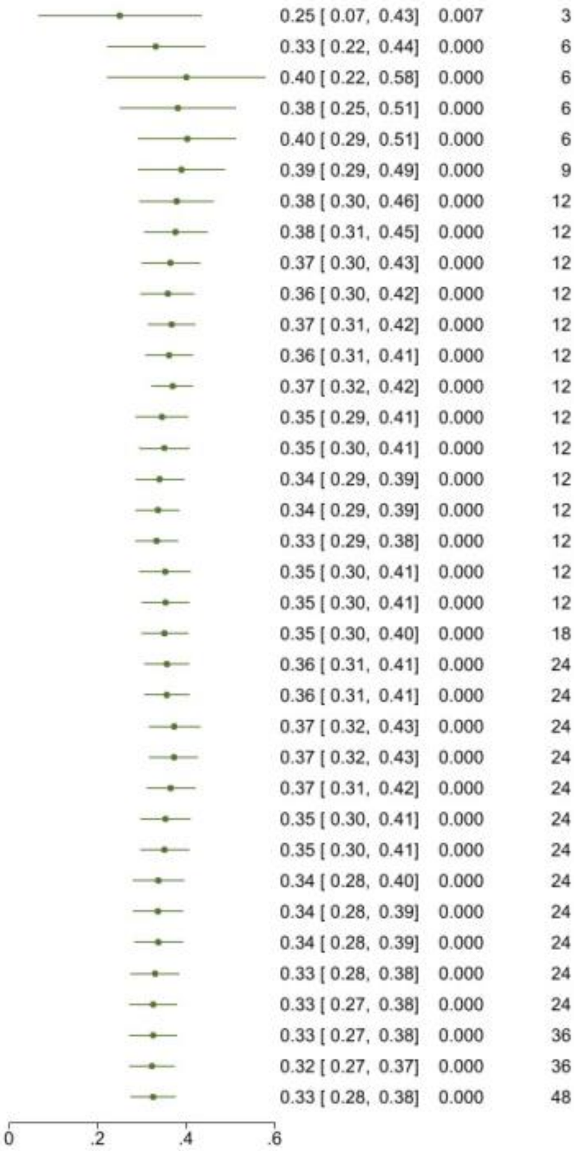

Random-effects REML model

3: the follow-up length less or equal to three months, 6:]3,6], 9:]6,9], 12:]9,12], 24:]12,24], 36:]24,36], 48:]36,48], and 120:]48,120] months

Figure S10. Cumulative meta-analysis of the association between Job demands and occupational burnout by increasing follow-up lengths

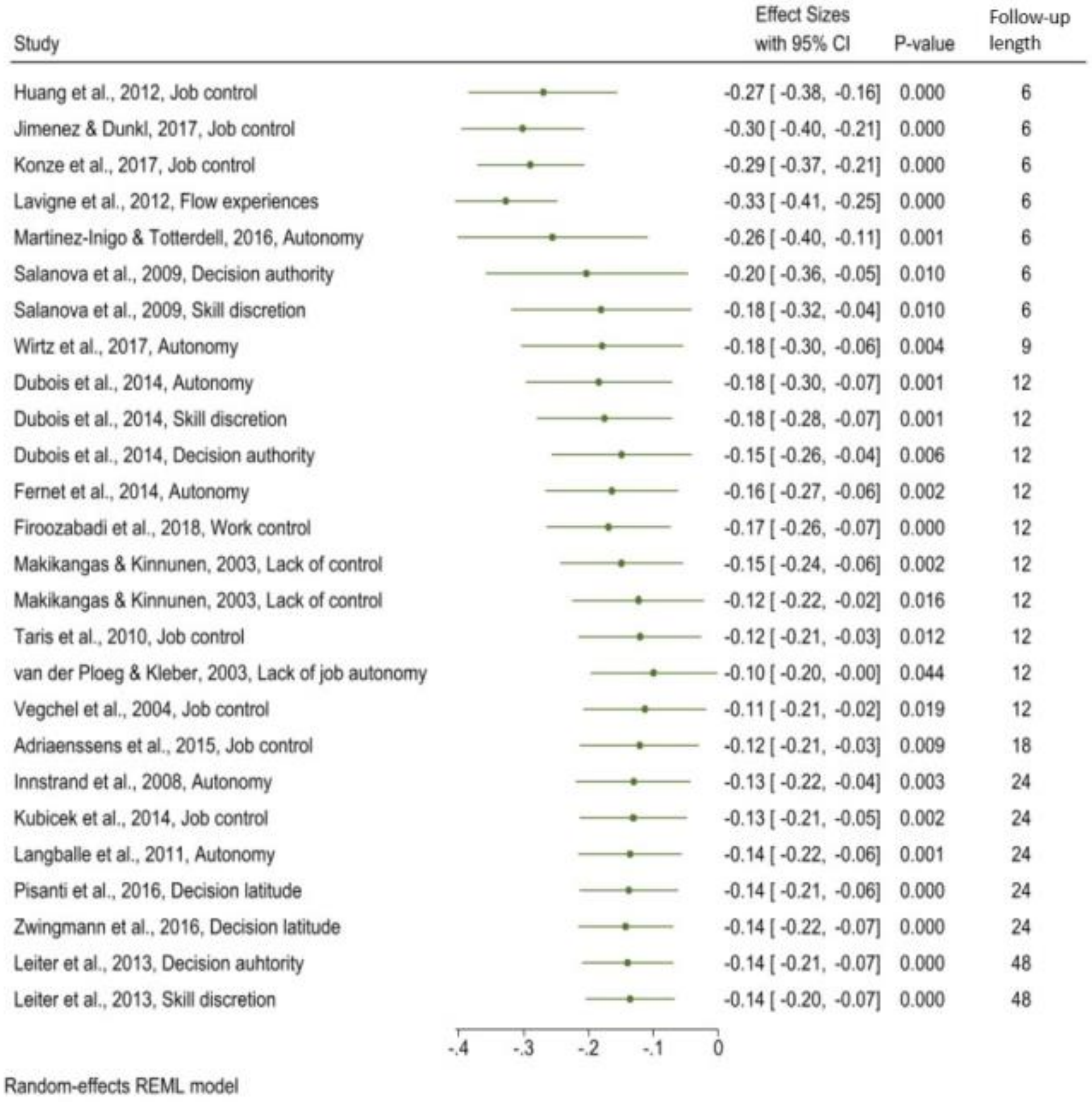

3: the follow-up length less or equal to three months, 6:]3,6], 9:]6,9], 12:]9,12], 24:]12,24], 36:]24,36], 48:]36,48], and 120:]48,120] months

Figure S11. Cumulative meta-analysis of the association between Job control and occupational burnout by increasing follow-up lengths

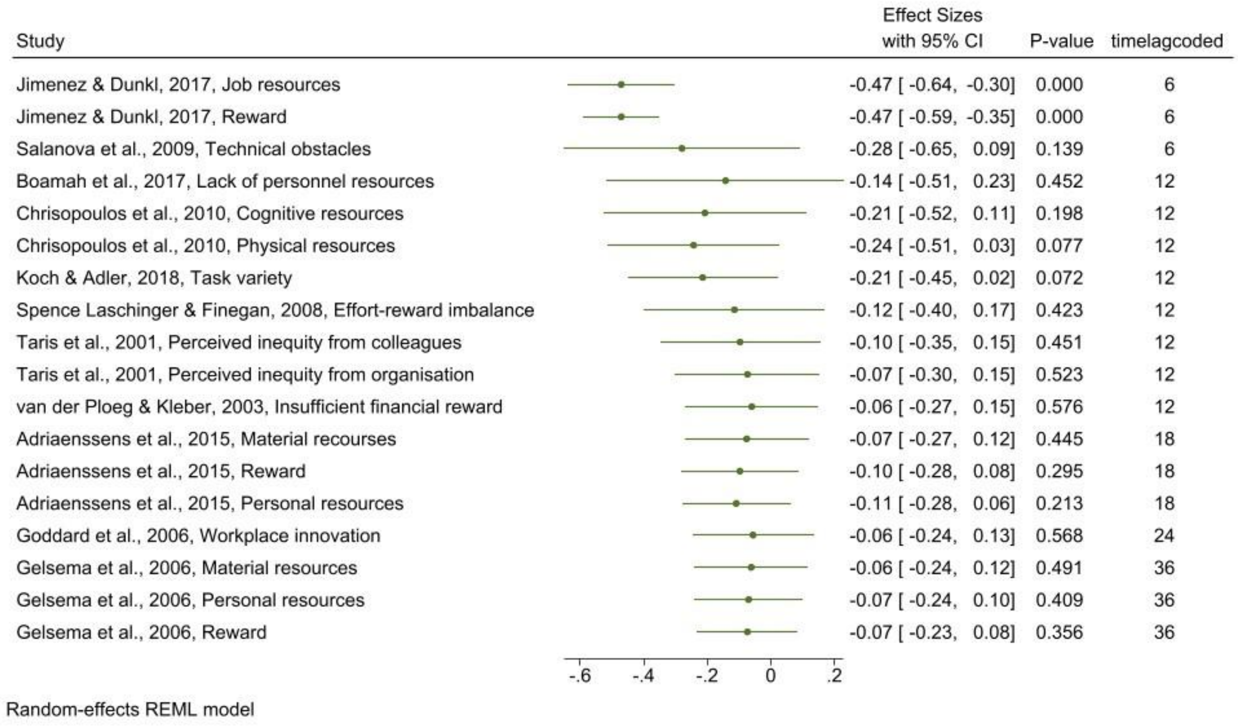

3: the follow-up length less or equal to three months, 6:]3,6], 9:]6,9], 12:]9,12], 24:]12,24], 36:]24,36], 48:]36,48], and 120:]48,120] months

Figure S12. Cumulative meta-analysis of the association between Job resources and occupational burnout by increasing follow0up lengths

Study		Effect Sizes with 95% CI	P-value	Follow-up length
Jimenez & Dunkl, 2017, Fairness		-0.38 [-0.55, -0.21]	0.000	6
Martinez-Inigo & Totterdell, 2016, Distributive justice		-0.35 [-0.45, -0.25]	0.000	6
Martinez-Inigo & Totterdell, 2016, Display rule monitoring		-0.21 [-0.49, 0.08]	0.156	6
Salanova et al., 2009, Social support		-0.17 [-0.38, 0.05]	0.130	6
Salanova et al., 2009, Conflicts and interpersonal problems		-0.10 [-0.31, 0.11]	0.334	6
Thompson, 2020 , Workplace ostracism		-0.05 [-0.25, 0.14]	0.595	6
Lapointe et al., 2013, Psychological contract breach		-0.02 [-0.20, 0.16]	0.842	9
Angelo & Chambel, 2015, Conflicts and interperosonal problems		0.01 [-0.15, 0.18]	0.882	12
Angelo & Chambel, 2015, Support from supervisor		0.01 [-0.13, 0.15]	0.913	12
Birkeland et al., 2018, Support from supervisor		-0.02 [-0.16, 0.12]	0.763	12
Birkeland et al., 2018, Support from colleagues		-0.04 [-0.17, 0.09]	0.580	12
Boamah et al., 2017, Structural empowerment		-0.05 [-0.17, 0.07]	0.429	12
Chrisopoulos et al., 2010, Emotional support		-0.08 [-0.20, 0.05]	0.222	12
Dubois et al., 2014, Support from supervisor		-0.09 [-0.21, 0.03]	0.150	12
Dubois et al., 2014, Support from colleagues		-0.09 [-0.20, 0.03]	0.133	12
Fida et al., 2018, Work incivilitiy from supervisor		-0.07 [-0.18, 0.04]	0.205	12
Fida et al., 2018, Work incivilitiy from coworker		-0.05 [-0.16, 0.06]	0.375	12
Fida et al., 2018, Work incivilitiy from physician		-0.03 [-0.14, 0.08]	0.574	12
Koch & Adler, 2018, Social support from Supervisor		-0.05 [-0.15, 0.06]	0.398	12
Lizano & Barak, 2012, Support from supervisor		-0.04 [-0.14, 0.06]	0.398	12
Lizano & Barak, 2012, Organizational support		-0.05 [-0.14, 0.05]	0.338	12
Makikangas & Kinnunen, 2003, Poor social climate		-0.03 [-0.13, 0.06]	0.480	12
Makikangas & Kinnunen, 2003, Poor social climate		-0.02 [-0.12, 0.07]	0.609	12
Van de Ven et al., 2013, Emotional support		-0.03 [-0.12, 0.06]	0.511	12
van der Ploeg & Kleber, 2003, Lack of social support from colleagues		-0.02 [-0.11, 0.07]	0.686	12
van der Ploeg & Kleber, 2003, Lack of social support from supervisor		-0.00 [-0.09, 0.09]	0.944	12
Vegchel et al., 2004, Social support		-0.02 [-0.11, 0.07]	0.691	12
Adriaenssens et al., 2015, Social support		-0.02 [-0.11, 0.07]	0.616	18
Adriaenssens et al., 2015, Social harassment		-0.02 [-0.11, 0.07]	0.637	18
Feuerhahn et al., 2013, Conflicts and interpersonal problems		-0.00 [-0.09, 0.09]	0.937	24
Feuerhahn et al., 2013, Emotional support		-0.01 [-0.11, 0.08]	0.761	24
Feuerhahn et al., 2013, Classroom disruption		0.00 [-0.09, 0.10]	0.945	24
Pisanti et al., 2016, Social support		0.00 [-0.09, 0.10]	0.937	24
Ramarajan et al., 2008, Organisational respect		-0.00 [-0.10, 0.09]	0.915	24
Zwingmann et al., 2016, Social support		-0.01 [-0.10, 0.08]	0.882	24
Gelsema et al., 2006, nurse-doctor collaboration		-0.01 [-0.10, 0.08]	0.809	36

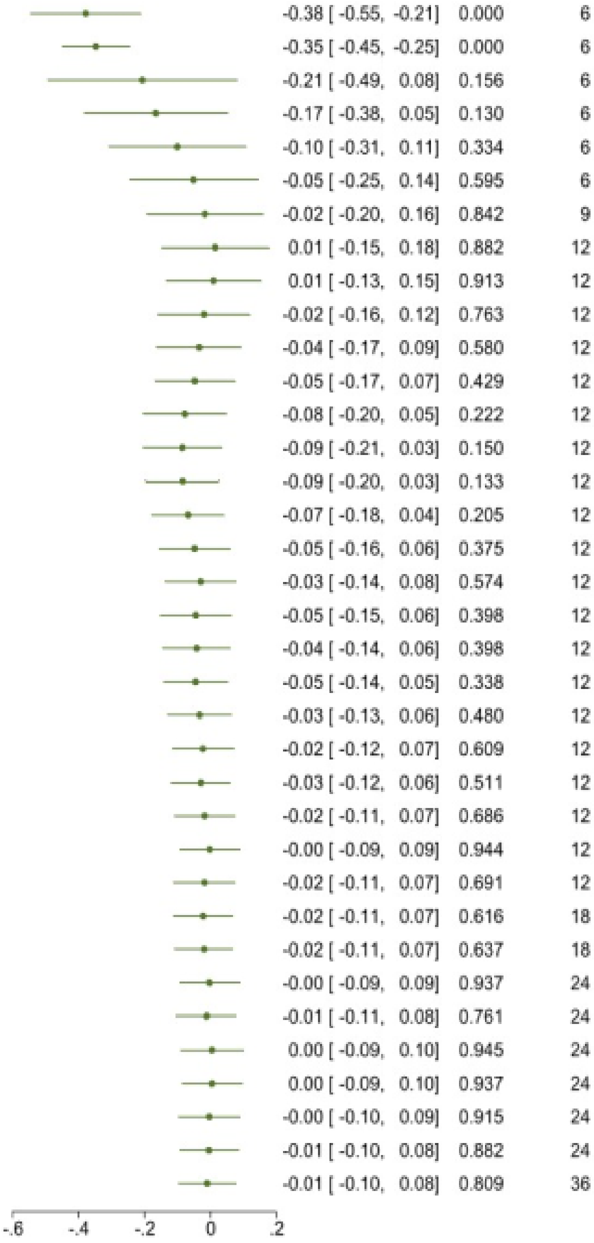

Random-effects REML model

3: the follow-up length less or equal to three months, 6:]3,6], 9:]6,9], 12:]9,12], 24:]12,24], 36:]24,36], 48:]36,48], and 120:]48,120] months

Figure S13. Cumulative meta-analysis of the association between Interactions at work and occupational burnout by increasing follow-up lengths

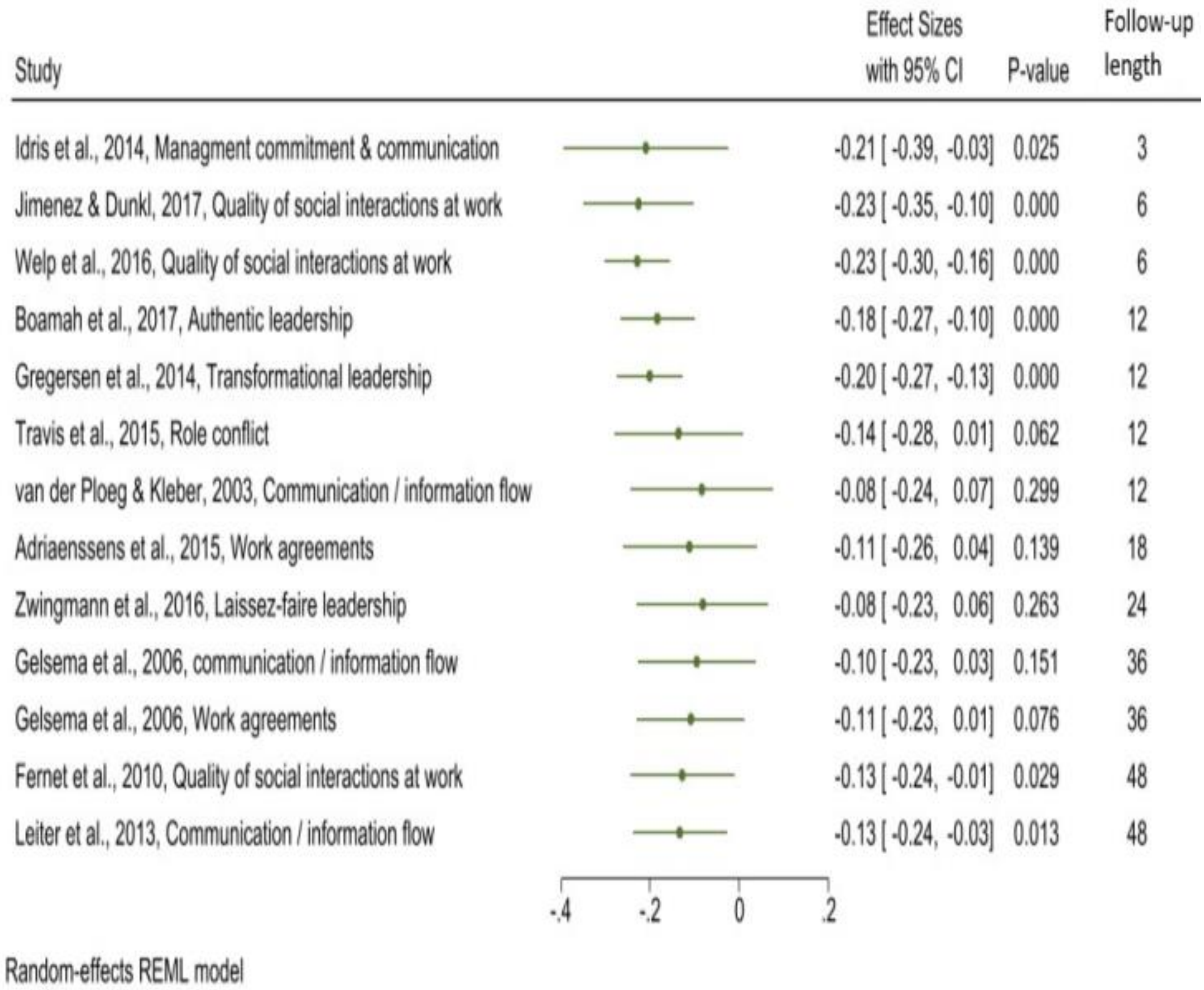

3: the follow-up length less or equal to three months, 6:]3,6], 9:]6,9], 12:]9,12], 24:]12,24], 36:]24,36], 48:]36,48], and 120:]48,120] months

Figure S14. Cumulative meta-analysis of the association between Communication and leadership and occupational burnout by increasing follow0up lengths

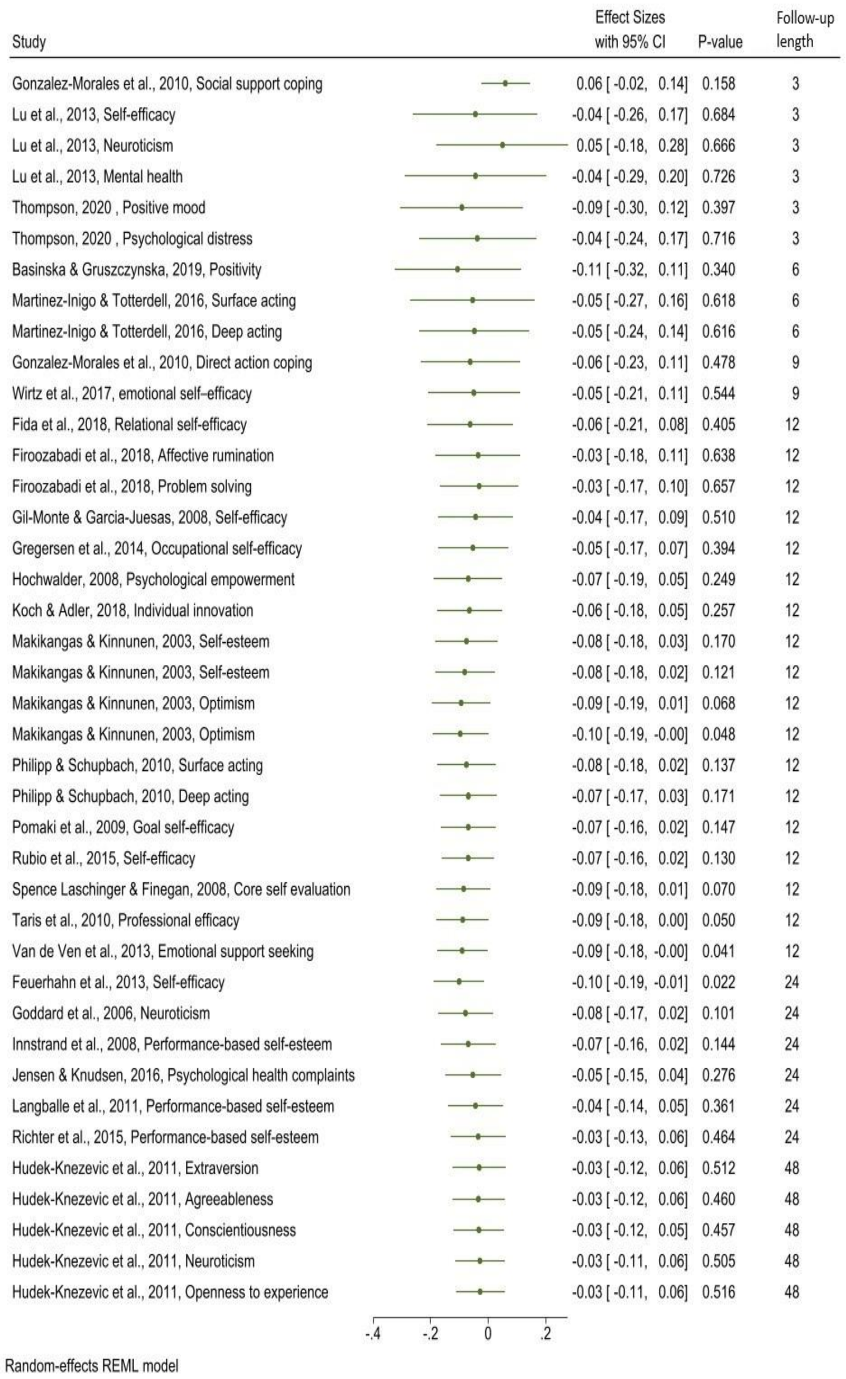

3: the follow-up length less or equal to three months, 6:]3,6], 9:]6,9], 12:]9,12], 24:]12,24], 36:]24,36], 48:]36,48], and 120:]48,120] months

Figure S15. Cumulative meta-analysis of the association between Personality characteristics and self-reported health status and occupational burnout by increasing follow0up lengths

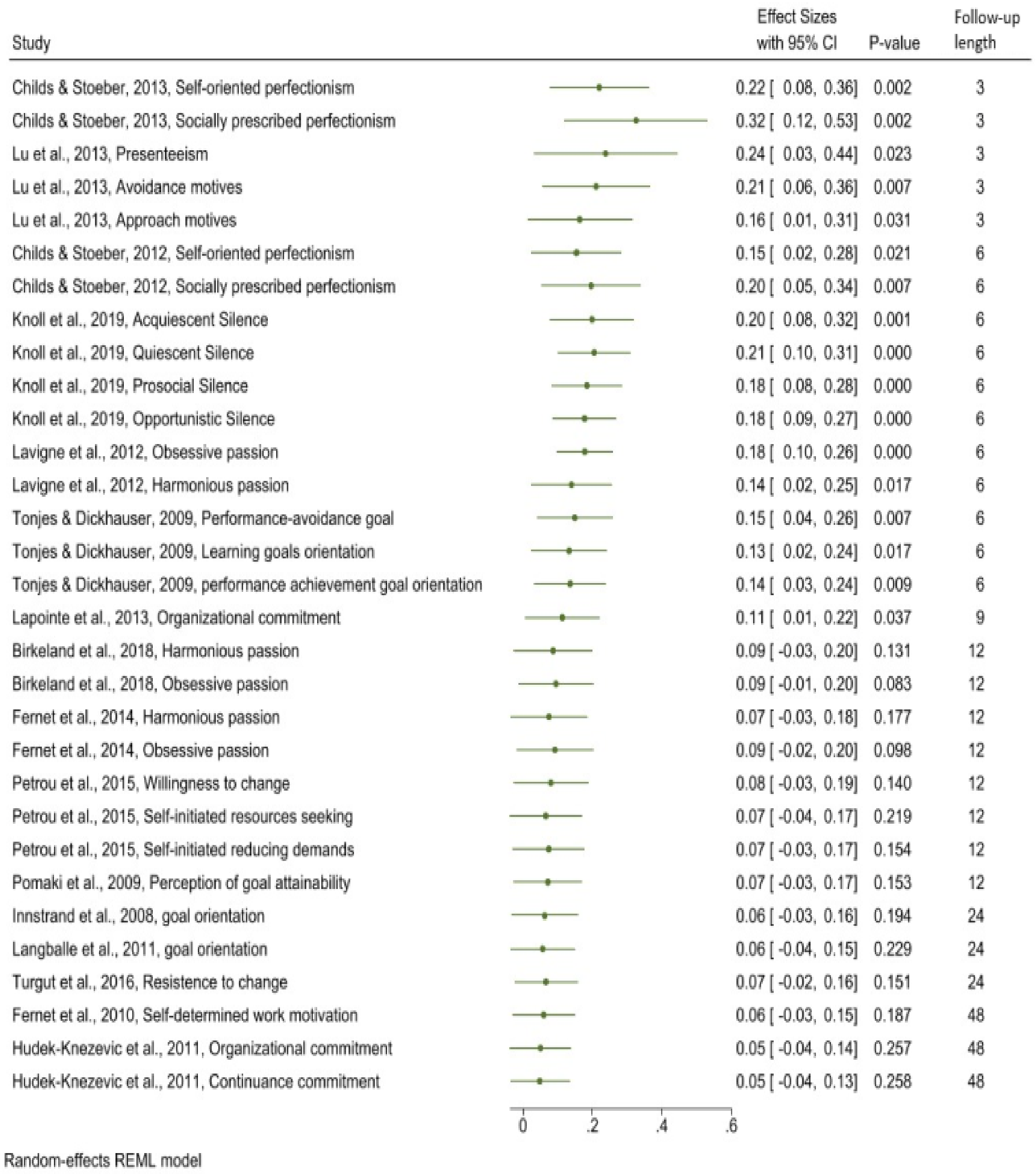

3: the follow-up length less or equal to three months, 6:]3,6], 9:]6,9], 12:]9,12], 24:]12,24], 36:]24,36], 48:]36,48], and 120:]48,120] months

Figure S16. Cumulative meta-analysis of the association between Job attitudes and occupational burnout by increasing follow0up lengths

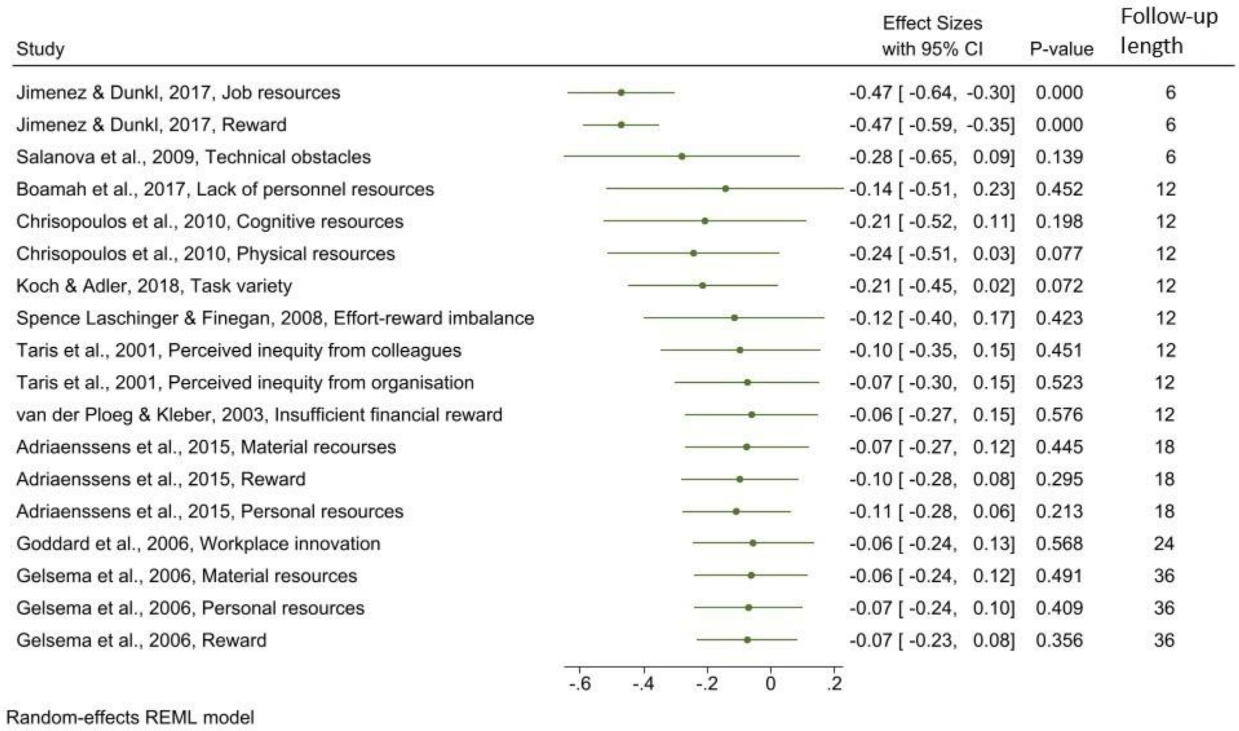

3: the follow-up length less or equal to three months, 6:]3,6], 9:]6,9], 12:]9,12], 24:]12,24], 36:]24,36], 48:]36,48], and 120:]48,120] months

Figure S17. Cumulative meta-analysis of the association between Work-life interface and occupational burnout by increasing follow-up lengths

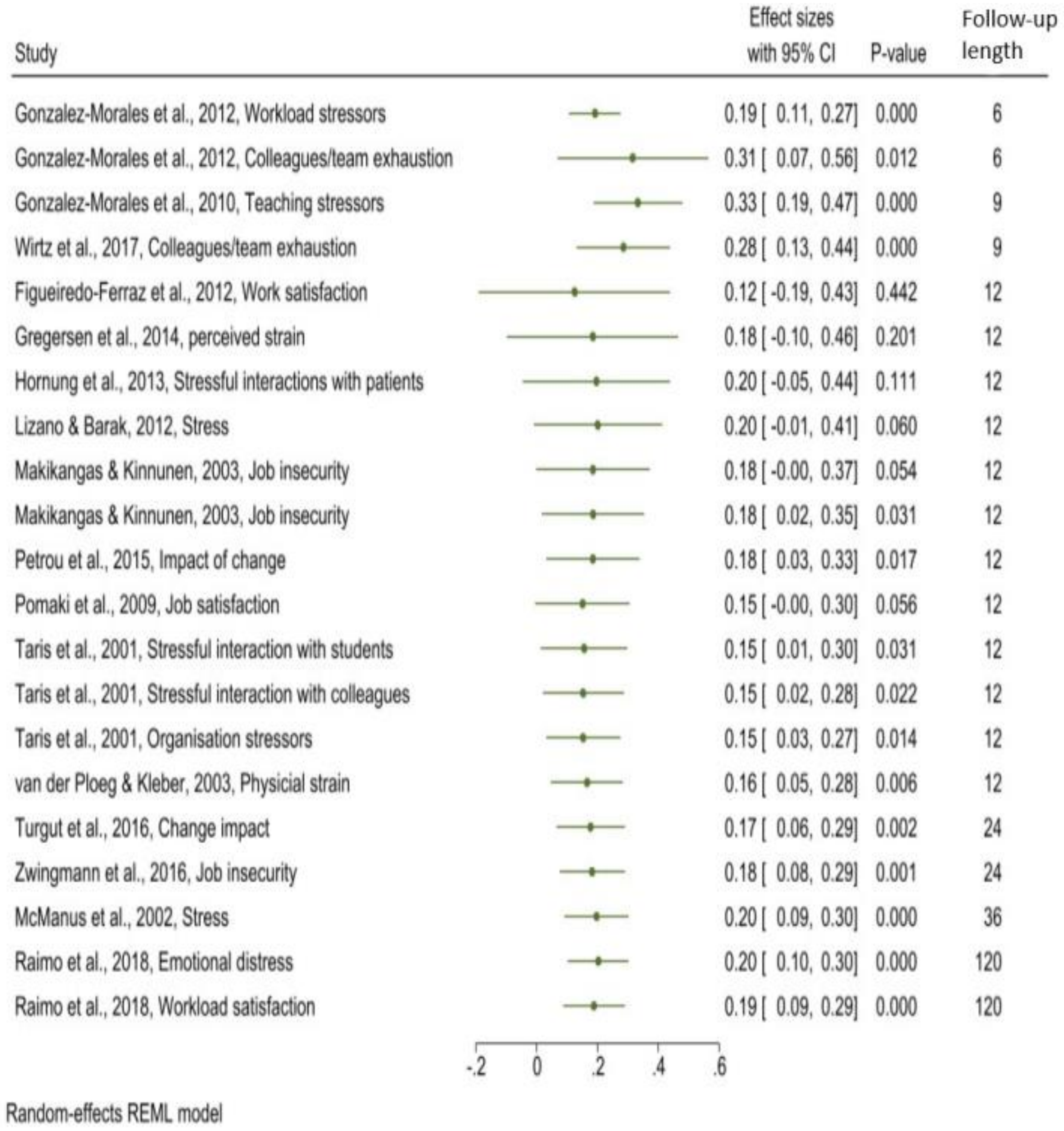

3: the follow-up length less or equal to three months, 6:]3,6], 9:]6,9], 12:]9,12], 24:]12,24], 36:]24,36], 48:]36,48], and 120:]48,120] months

Figure S18. Cumulative meta-analysis of the association between Perceived intermediate work consequences and occupational burnout by increasing follow-up lengths

Supplementary Table S2. Results of the sensitivity analysis if deleting one study changes the summary estimate of associations between subgroups of predictor-variables and exhaustion, and the heterogeneity of these summary estimates

**Subgroup name**	**Deleted study**	**Number of studies**	**Heterogeneity I² (before sensitivity analysis)**	**Summary estimate (before sensitivity analysis)**	**Heterogeneity I² (after sensitivity analysis)**	**Summary estimate (after sensitivity analysis)**	**95% confidence interval**
Cognitive demands	Chrisopoulos et al., 2010	2	89.74%	0.13	56.71%	0.05	-0.04, 0.13
Workload	Jimenez & Dunkl, 2017	5	18.69%	0.38	0.02%	0.36	0.33, 0.40
Emotional demands	Van de Ven et al., 2013	7	31.69%	0.34	17.97	0.34	0.30, 0.39
Emotional demands	Vegchel et al., 2004	7	31.69%	0.34	8.21%	0.32	0.27, 0.37
Autonomy	Martinez-Inigo & Totterdell, 2016	5	77.82%	-0.21	0.00%	-0.27	-0.31, -0.23
Job control	Taris et al., 2010	7	76.78%	-0.23	56.29%	-0.26	-0.32, -0.22
Lack of job resources	Boamah et al., 2017	3	73.40%	0.12	0.01%	0.06	0.01, 0.11
Material resources	Chrisopoulos et al., 2010	2	72.77%	-0.27	0.00%	-0.19	-0.28, -0.11
Reward	Gelsema et al., 2006	2	83.64%	-0.32	0.00%	-0.42	-0.53, 0.30
Material resources	Gelsema et al., 2006	2	72.77%	-0.27	59.78%	-0.34	-0.50, -0.17
Poor social climate	Adriaenssens et al., 2015	5	66.60%	0.24	0.01%	0.24	0.19, 0.29
Poor social climate	Feuerhahn et al., 2013	5	66.60%	0.24	30.38%	0.20	0.15, 0.26

Conflict & interpersonal problems	Feuerhahn et al., 2013	2	92.19%	0.30	0.00%	0.20	0.16, 0.25
Quality of social interactions at work	Fernet et al., 2010	2	20.20%	-0.27	0.00%	-0.23	-0.31, -0.15
Communication/ information flow	van der Ploeg & Kleber, 2003	3	94.05%	-0.09	0.01%	-0.18	-0.21, -0.15
Quality of social interactions at work	Welp et al., 2016	2	20.20%	-0.27	0.01%	-0.31	-0.40, -0.21
Unvalued trait/ characteristics	Goddard et al., 2006	2	90.29%	0.32	11.34%	0.21	0.10, 0.32
Adaptive coping	Gonzalez-Morales et al., 2010	3	73.55%	-0.02	0.00%	0.04	-0.06, 0.13
Self-efficacy	Hochwalder, 2008	9	70.20%	-0.19	0.00%	-0.17	-0.21, -0.13
Performance-based self-esteem	Innstrand et al., 2008	2	45.52%	0.24	0.01%	0.26	0.23, 0.29
Performance-based self-esteem	Richter et al., 2015	2	45.52%	0.24	0.00%	0.22	0.18, 0.25
Self-esteem	Spence Laschinger & Finegan, 2008	1	83.22%	-0.33	0.00%	-0.23	-0.32, -0.14
Intrinsically motivated behavior	Childs & Stoeber, 2013, the Second study	7	86.28%	-0.07	53.96%	-0.12	-0.18, -0.06
Negative job attitudes	Lu et al., 2013	5	79.93%	0.25	58.87%	0.27	0.21, 0.33

Work-family conflict	Langballe et al., 2011	9	49.36%	0.36	0.06%	0.36	0.34, 0.38
Work-family conflict	Lizano & Barak, 2012	9	49.36%	0.36	0.02%	0.37	0.35, 0.39
Work-family conflict	Rubio et al., 2015	9	49.36%	0.36	0.00%	0.37	0.35, 0.39
Work-family conflict	Travis et al., 2015	9	49.36%	0.36	0.04%	0.37	0.35, 0.39
Work-family Facilitation	Innstrand et al., 2011	2	71.24%	-0.11	0.00 %	-0.15	-0.22, -0.09
Family-work Facilitation	Innstrand et al., 2011	2	57.95%	-0.05	0.01%	-0.09	-0.16, -0.02
Family-work Facilitation	Langballe et al., 2011	2	57.95%	-0.05	24.09%	-0.01	-0.07, 0.04
Value congruency	Langballe et al., 2011	2	54.12%	-0.27	23.41%	-0.30	-0.37, -0.22
Satisfaction	Figueiredo-Ferraz et al., 2012	2	75.43%	-0.29	0.00%	-0.20	-0.33, -0.08
Work stressors	Gonzalez-Morales et al., 2010	3	80.55%	0.24	0.00%	0.17	0.12, 0.22

Supplementary Table S3. Comparison of Meta-analysis results per subgroups of studied predictor-variables between overall studies and studies conducted in Europe

**Studied predictor-variables grouped per (sub)family**	**Number of studies (overall)**	**Heterogeneity "I² estimate"**	**Summary estimate of the association with exhaustion**	**95% Confidence interval**	**Number of studies conducted in Europe**	**Heterogeneity "I² estimate"**	**Summary estimate of the association with exhaustion**	**95% Confidence interval**
Job demands	***27***	***89.25%***	***0.33***	***0.28-0.38***	***22***	***89.08%***	***0.31***	***0.26-0.37***

Work and time demands	8	91.40%	0.33	0.22-0.43	7	92.85%	0.32	0.20, 0.44
Cognitive demands	3	89.74%	0.13	-0.05, 0.31	2	56.71%	0.05	-0.04, 0.13
Physical demands	2	0.00%	0.25	0.17, 0.34	1	0.00%	0.24	0.14, 0.34
Workload	6	18.69%	0.38	0.34-0.43	6	18.69%	0.38	0.34-0.43
Time pressure	5	92.34%	0.35	0.17-0.53	5	63.90%	0.27	0.16-0.38
Job demands (overall)	2	13.55%	0.35	0.23-0.48	2	13.55%	0.35	0.23-0.48
Emotional demands	8	31.69%	0.34	0.30-0.39	6	48%	0.35	0.29-0.40
Job control	***20***	***94.14%***	***-0.15***	***-0.21, -0.09***	***18***	***94.10%***	***-0.13***	***-0.20, -0.06***
Job control	8	76.78%	-0.23	-0.30, -0.16	6	83.80%	-0.23	-0.23, -0.13
Skill discretion	3	0.00%	-0.05	-0.08, -0.02	2	0.00%	-0.05	-0.08, -0.02
Autonomy	6	77.82%	-0.21	-0.21, -0.11	4	86.22%	-0.18	-0.31, -0.04
Decision authority	5	81.59%	-0.06	-0.19, 0.06	4	76.17%	-0.10	-0.21, 0.01
Flow experiences	1	NA	-0.40	-0.51, -0.29	NA	NA	NA	NA
Lack of control	2	38.44%	0.17	0.07, 0.28	2	56.26%	0.15	-0.01, 0.32
Job resources	***11***	***97.22%***	***-0.07***	***-0.23, 0.08***	***7***	***92.55%***	***-0.15***	***-0.26, -0.04***
Job resources	6	97.75%	-0.12	-0.47, 0.22	4	85.12%	-0.23	-0.40, -0.07

Lack of job resources	4	73.40%	0.12	0.02, 0.23	3	0.01%	0.06	0.01, 0.11
Reward	3	83.64%	-0.32	-0.51, -0.12	3	83.64%	-0.32	-0.51, -0.12
Lack of reward/inequity	2	96.27%	0.35	-0.12, 0.82	1	NA	0.12	0.06, 0.18
Material resources	3	72.77%	-0.27	-0.42, -0.13	2	0.00%	-0.19	-0.28, -0.11
Interactions at work	***23***	***96.57%***	***-0.02***	***-0.10, 0.07***	***17***	***97.34%***	***-0.02***	***-0.13, 0.10***
Social support	12	89.24%	-0.18	-0.27, -0.08	8	90.47%	-0.14	-0.26, -0.01
Poor social climate	5	79.37%	0.24	0.12, 0.35	3	90.96%	0.29	-0.04, 0.62
Support from supervisor	3	91.71%	-0.16	-0.29, -0.03	3	95.30%	-0.20	-0.37, -0.03
Support from colleagues	3	0.01%	-0.16	-0.21, -0,12	2	0.02%	-0.17	-0.22, -0,12
Fairness/justice	2	0.00%	-0.35	-0.45, -0.25	2	0.00%	-0.35	-0.45, -0.25
Lack of support from supervisor	2	85.25%	0.27	0.01, 0.52	2	NA	0.41	0.23, 0.59
Lack of support from coworkers	2	0.01%	0.27	0.20, 0.35	1	NA	0.29	0.11, 0.47
Conflict & interpersonal problems	3	92.19%	0.30	0.05, 0.55	3	92.19%	0.30	0.05, 0.55

Communication & leadership	***12***	***93.09%***	***-0.13***	***-0.24, -0.03***	***8***	***93.09%***	***-0.13***	***-0.24, -0.03***
Work agreements	2	0.00%	-0.25	-0.33, -0.16	2	0.00%	-0.25	-0.33, -0.16
Communication/ information flow	4	94.05%	-0.09	-0.30, 0.12	3	96.43%	-0.05	-0.33, 0.23
Quality of social interactions at work	3	20.20%	-0.27	-0.34, -0.19	2	0.00%	-0.23	-0.31, -0.15
Leadership	3	90.31%	-0.07	-0.31, 0.17	2	90.31%	-0.07	-0.31, 0.17
Role conflict	1	NA	0.19	0.09, 0.29	NA	NA	NA	NA
Personality characteristics & self-reported health status	***26***	***96.60%***	***-0.02***	***-0.11, 0.07***	***18***	***96.56%***	***-0.02***	***-0.11, 0.07***
Unvalued trait/ characteristics	3	90.29%	0.32	0.07, 0.57	1	NA	0.13	-0.05, 0.31
Valued trait/ characteristics	5	88.15%	-0.24	-0.39, -0.09	4	91.79%	-0.25	-0.48, -0.01
Extraversion	1	NA	0.13	-0.05, 0.31	1	NA	0.13	-0.05, 0.31
Conscientiousness	1	NA	-0.01	-0.19, 0.17	1	NA	-0.01	-0.19, 0.17
Openness	1	NA	0.03	-0.15, 0.21	1	NA	0.03	-0.15, 0.21
Self-efficacy	10	70.20%	-0.19	-0.25, -0.12	8	80.59%	-0.18	-0.28, -0.09

Maladaptive coping	3	0.00%	0.33	0.24, 0.42	2	0.00%	0.34	0.23, 0.45
Adaptive coping	4	73.55%	-0.02	-0.16, 0.11	3	81.69%	-0.03	-0.21, 0.14
Emotion-focused coping	2	87.42%	-0.02	-0.18, 0.14	2	87.42%	-0.02	-0.18, 0.14
Self-esteem	2	83.22%	-0.33	-0.53, -0.13	2	NA	-0.27	-0.40, -0.14
Performance-based self-esteem	3	45.52%	0.24	0.20, 0.28	3	45.52%	0.24	0.20, 0.28
Self-reported health status (harmful)	2	92.14%	0.34	0.13, 0.55	1	NA	0.44	0.39, 0.49
Self-reported health status (protective)	1	NA	-0.33	-0.46, -0.20	NA	NA	NA	NA
Job attitudes	***18***	***95.73%***	***0.05***	***-0.04, 0.13***	***10***	***95.99%***	***0.08***	***-0.02, 0.18***
Positive job attitudes	7	79.71%	-0.24	-0.33, -0.15	4	87.22%	-0.19	-0.33, -0.04
Negative job attitudes	6	79.93%	0.25	0.17, 0.33	3	46.75%	0.26	0.21, 0.32
Intrinsically motivated behavior	8	86.28%	-0.07	-0.17, 0.03	6	90.82%	-0.06	-0.20, 0.07
Extrinsically motivated behavior	4	83.30%	0.28	0.05, 0.51	4	83.30%	0.28	0.05, 0.51

Avoidance motives	2	54.33%	0.20	0.03, 0.37	1	NA	0.31	0.11, 0.51
Acquiescent silence	1	NA	0.22	0.14, 0.30	1	NA	0.22	0.14, 0.30
Quiescent silence	1	NA	0.26	0.18, 0.34	1	NA	0.26	0.18, 0.34
Prosocial silence	1	NA	0.01	-0.07, 0.09	1	NA	0.01	-0.07, 0.09
Opportunistic silence	1	NA	0.13	0.05, 0.21	1	NA	0.13	0.05, 0.21
Work-family interface	***11***	***98.35%***	***0.13***	***0.02, 0.23***	***7***	***98.57%***	***0.10***	***-0.02, 0.21***
Work-family conflict	10	49.36%	0.36	0.33, 0.39	7	0.02%	0.37	0.35, 0.39
Family-work conflict	3	0.00%	0.20	0.17, 0.24	3	0.00%	0.20	0.17, 0.24
Work-family facilitation	3	71.24%	-0.11	-0.19, -0.02	3	71.24%	-0.11	-0.19, -0.02
Family-work facilitation	3	57.95%	-0.05	-0.11, 0.02	3	57.95%	-0.05	-0.11, 0.02
Value congruency	3	54.12%	-0.27	-0.34,-0.20	3	54.12%	-0.27	-0.34,- 0.20
Perceived intermediate work consequences	***16***	***95.04%***	***0.19***	***0.09, 0.29***	***15***	***96.07%***	***0.19***	***0.08, 0.31***
Work stressors	4	80.55%	0.24	0.13, 0.35	3	87.86%	0.23	0.11, 0.36

Stressful interactions with patients/students	2	0.00%	0.22	0.16, 0.28	2	0.00%	0.22	0.16, 0.28
Job insecurity	2	56.18%	0.16	0.03, 0.30	2	73.48%	0.16	-0.09, 0.41
Impact of change	2	90.29%	0.26	0.08, 0.44	2	90.29%	0.26	0.08, 0.44
Psychological/physical toll	2	33.39%	0.44	0.31, 0.56	2	33.39%	0.44	0.31, 0.56
Stress from work	3	93.06%	0.26	0.06, 0.46	2	96.75%	0.28	-0.06, 0.61
Satisfaction	3	75.43%	-0.29	-0.47, -0.11	2	81.24%	-0.35	-0.56, -0.13
Colleagues/team exhaustion	2	88.04%	0.27	-0.10, 0.64	2	88.04%	0.27	-0.10, 0.64

Supplementary Table S4. Comparison of Meta-analysis results per subgroups of studied predictor-variables between overall studies and studies conducted for medical and health workers

**Studied predictor-variables grouped per (sub)family**	**Number of studies (overall)**	**Heterogeneity "I² estimate"**	**Summary estimate of the association with exhaustion**	**95% Confidence interval**	**Number of studies conducted for medical and health workers**	**Heterogeneity "I² estimate"**	**Summary estimate of the association with exhaustion**	**95% Confidence interval**
Job demands	***27***	***89.25%***	***0.33***	***0.28-0.38***	***10***	***90.64%***	***0.28***	***0.20-0.35***

Work and time demands	8	91.40%	0.33	0.22-0.43	5	94.90%	0.33	0.16, 0.50
Cognitive demands	3	89.74%	0.13	-0.05, 0.31	2	56.71%	0.05	-0.04, 0.13
Physical demands	2	0.00%	0.25	0.17, 0.34	1	NA	0.24	0.14, 0.34
Workload	6	18.69%	0.38	0.34-0.43	2	0.00%	0.27	0.12-0.43
Time pressure	5	92.34%	0.35	0.17-0.53	2	47.15%	0.26	0.11-0.41
Job demands (overall)	2	13.55%	0.35	0.23-0.48	2	13.55%	0.35	0.23-0.48
Emotional demands	8	31.69%	0.34	0.30-0.39	3	40.87%	0.34	0.28-0.40
Job control	***20***	***94.14%***	***-0.15***	***-0.21, -0.09***	***11***	***92.97%***	***-0.12***	***-0.20, -0.04***
Job control	8	76.78%	-0.23	-0.30, -0.16	4	71.84%	-0.24	-0.33, -0.15
Skill discretion	3	0.00%	-0.05	-0.08, -0.02	2	0.01%	-0.05	-0.08, -0.02
Autonomy	6	77.82%	-0.21	-0.21, -0.11	3	54.70%	-0.11	-0.27, 0.05
Decision authority	5	81.59%	-0.06	-0.19, 0.06	4	79.11%	-0.10	-0.24, 0.05
Flow experiences	1	NA	-0.40	-0.51, -0.29	NA	NA	NA	NA
Lack of control	2	38.44%	0.17	0.07, 0.28	1	NA	0.25	0.07, 0.43
Job resources	***11***	***97.22%***	***-0.07***	***-0.23, 0.08***	***5***	***95.62%***	***-0.05***	***-0.25, 0.15***
Job resources	6	97.75%	-0.12	-0.47, 0.22	2	0.00%	-0.22	-0.30, -0.13

Lack of job resources	4	73.40%	0.12	0.02, 0.23	2	62.73%	0.19	0.03, 0.36
Reward	3	83.64%	-0.32	-0.51, -0.12	2	83.64%	-0.25	-0.47, -0.02
Lack of reward/inequity	2	96.27%	0.35	-0.12, 0.82	1	NA	0.60	0.43, 0.77
Material resources	3	72.77%	-0.27	-0.42, -0.13	2	0.00%	-0.19	-0.28, -0.11
Interactions at work	***23***	***96.57%***	***-0.02***	***-0.10, 0.07***	***13***	***94.14%***	***-0.02***	***-0.13, 0.08***
Social support	12	89.24%	-0.18	-0.27, -0.08	8	88.65%	-0.14	-0.26, -0.03
Poor social climate	5	79.37%	0.24	0.12, 0.35	2	85.54%	0.16	-0.06, 0.39
Support from supervisor	3	91.71%	-0.16	-0.29, -0.03	2	70.67%	-0.09	-0.30, 0.11
Support from colleagues	3	0.01%	-0.16	-0.21, -0,12	2	0.01%	-0.12	-0.21, -0,03
Fairness/justice	2	0.00%	-0.35	-0.45, -0.25	1	NA	-0.33	-0.46, -0.20
Lack of support from supervisor	2	85.25%	0.27	0.01, 0.52	2	85.25%	0.27	0.01, 0.52
Lack of support from coworkers	2	0.01%	0.27	0.20, 0.35	2	0.01%	0.27	0.20, 0.35
Conflict & interpersonal problems	3	92.19%	0.30	0.05, 0.55	NA	NA	NA	NA

Communication & leadership	***12***	***93.09%***	***-0.13***	***-0.24, -0.03***	***8***	***92.99%***	***-0.13***	***-0.25, -0.01***
Work agreements	2	0.00%	-0.25	-0.33, -0.16	2	0.00%	-0.25	-0.33, -0.16
Communication/ information flow	4	94.05%	-0.09	-0.30, 0.12	3	96.43%	-0.05	-0.33, 0.23
Quality of social interactions at work	3	20.20%	-0.27	-0.34, -0.19	1	NA	-0.23	-0.32, -0.14
Leadership	3	90.31%	-0.07	-0.31, 0.17	3	90.31%	-0.07	-0.31, 0.17
Role conflict	1	NA	0.19	0.09, 0.29	NA	NA	NA	NA
Personality characteristics & self-reported health status	***26***	***96.60%***	***-0.02***	***-0.11, 0.07***	***11***	***91.88%***	***-0.03***	***-0.14, 0.08***
Unvalued trait/ characteristics	3	90.29%	0.32	0.07, 0.57	1	NA	0.13	-0.05, 0.31
Valued trait/ characteristics	5	88.15%	-0.24	-0.39, -0.09	1	NA	-0.15	-0.33, 0.03
Extraversion	1	NA	0.13	-0.05, 0.31	1	NA	0.13	-0.05, 0.31
Conscientiousness	1	NA	-0.01	-0.19, 0.17	1	NA	-0.01	-0.19, 0.17
Openness	1	NA	0.03	-0.15, 0.21	1	NA	0.03	-0.15, 0.21
Self-efficacy	10	70.20%	-0.19	-0.25, -0.12	6	73.80%	-0.18	-0.27, -0.10

Maladaptive coping	3	0.00%	0.33	0.24, 0.42	2	0.00%	0.31	0.21, 0.41
Adaptive coping	4	73.55%	-0.02	-0.16, 0.11	2	0.00%	0.01	-0.09, 0.11
Emotion-focused coping	2	87.42%	-0.02	-0.18, 0.14	NA	NA	NA	NA
Self-esteem	2	83.22%	-0.33	-0.53, -0.13	1	NA	-0.54	-0.71, -0.37
Performance-based self-esteem	3	45.52%	0.24	0.20, 0.28	1	NA	0.25	0.16, 0.34
Self-reported health status (harmful)	2	92.14%	0.34	0.13, 0.55	NA	NA	NA	NA
Self-reported health status (protective)	1	NA	-0.33	-0.46, -0.20	NA	NA	NA	NA
Job attitudes	***18***	***95.73%***	***0.05***	***-0.04, 0.13***	***3***	***86.18%***	***0.07***	***-0.17, 0.30***
Positive job attitudes	7	79.71%	-0.24	-0.33, -0.15	2	74.76%	-0.10	-0.34, 0.13
Negative job attitudes	6	79.93%	0.25	0.17, 0.33	NA	NA	NA	NA
Intrinsically motivated behavior	8	86.28%	-0.07	-0.17, 0.03	1	NA	0.08	-0.16, 0.32
Extrinsically motivated behavior	4	83.30%	0.28	0.05, 0.51	2	91.26%	0.25	-0.26, 0.76

Avoidance motives	2	54.33%	0.20	0.03, 0.37	NA	NA	NA	NA
Acquiescent silence	1	NA	0.22	0.14, 0.30	NA	NA	NA	NA
Quiescent silence	1	NA	0.26	0.18, 0.34	NA	NA	NA	NA
Prosocial silence	1	NA	0.01	-0.07, 0.09	NA	NA	NA	NA
Opportunistic silence	1	NA	0.13	0.05, 0.21	NA	NA	NA	NA
Work-family interface	***11***	***98.35%***	***0.13***	***0.02, 0.23***	***4***	***96.22%***	***0.22***	***0.03, 0.41***
Work-family conflict	10	49.36%	0.36	0.33, 0.39	4	60.33%	0.39	0.30, 0.48
Family-work conflict	3	0.00%	0.20	0.17, 0.24	1	NA	0.19	0.10, 0.28
Work-family facilitation	3	71.24%	-0.11	-0.19, -0.02	1	NA	-0.15	-0.24, -0.06
Family-work facilitation	3	57.95%	-0.05	-0.11, 0.02	1	NA	-0.10	-0.19, -0.01
Value congruency	3	54.12%	-0.27	-0.34,-0.20	NA	NA	NA	NA
Perceived intermediate work consequences	***16***	***95.04%***	***0.19***	***0.09, 0.29***	***15***	***96.07%***	***0.19***	***0.08, 0.31***
Work stressors	4	80.55%	0.24	0.13, 0.35	1	NA	0.30	0.08, 0.54

Stressful interactions with patients/students	2	0.00%	0.22	0.16, 0.28	1	NA	0.27	0.07, 0.47
Job insecurity	2	56.18%	0.16	0.03, 0.30	1	NA	0.31	0.08, 0.54
Impact of change	2	90.29%	0.26	0.08, 0.44	2	90.29%	0.26	0.08, 0.44
Psychological/physical toll	2	33.39%	0.44	0.31, 0.56	2	33.39%	0.44	0.31, 0.56
Stress from work	3	93.06%	0.26	0.06, 0.46	2	88.09%	0.34	0.12, 0.56
Satisfaction	3	75.43%	-0.29	-0.47, -0.11	3	75.43%	-0.29	-0.47, -0.11
Colleagues/team exhaustion	2	88.04%	0.27	-0.10, 0.64	1	93.98%	0.15	-0.04, 0.33

Supplementary Table S5. Results of the Regression-based Egger test for small-study effects per subfamily

Subfamily name	Regression coefficient	Standard error	p-value
Job demands	0.16	0.90	0.86
Job control	0.44	1.16	0.71
Job resources	-5.44	4.41	0.22
Interactions at work	1.71	1.45	0.24
Communication and leadership	2.60	2.13	0.22
Personality and self-reported health status	-1.19	1.64	0.47
Job attitudes	1.66	1.61	0.30
Work-family-interface	0.05	2.65	0.98
Perceived intermediate work consequences	-1.16	1.78	0.51

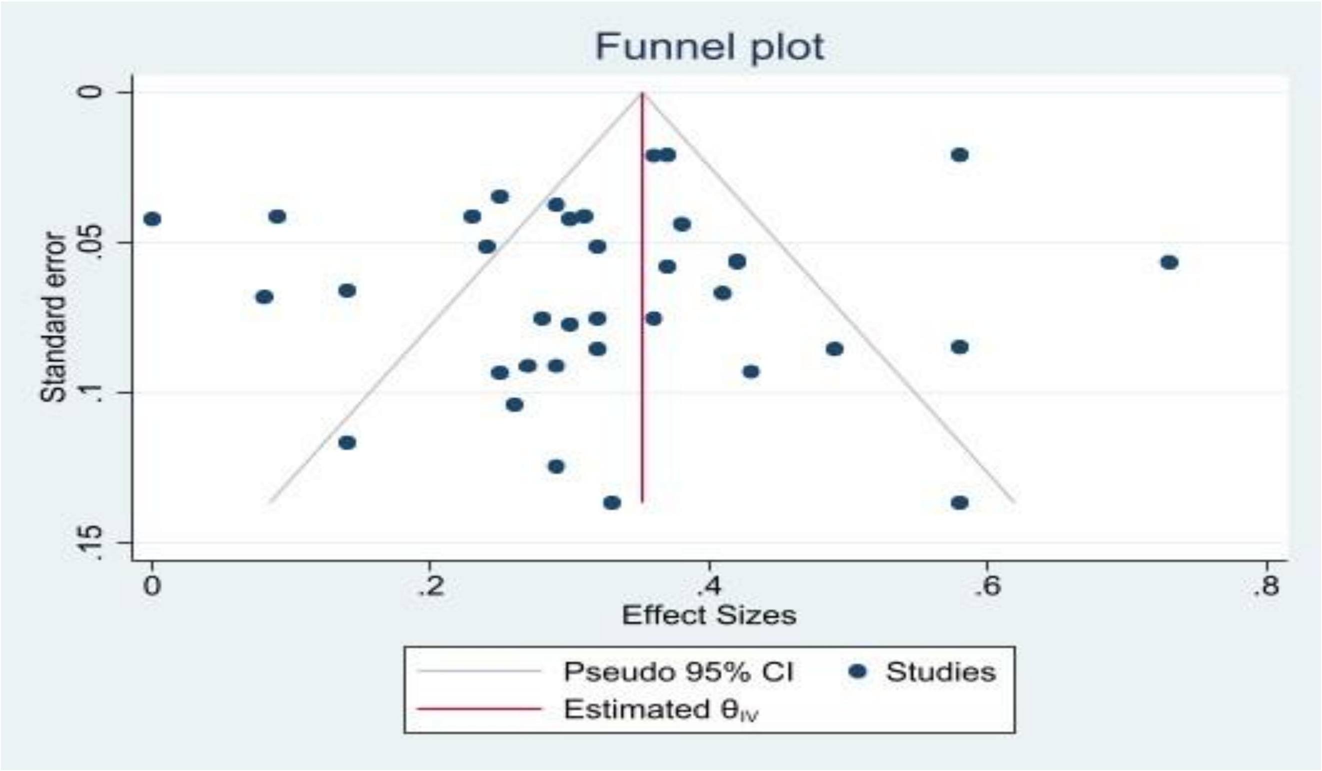

Supplementary Figure S19. Job demands funnel plot

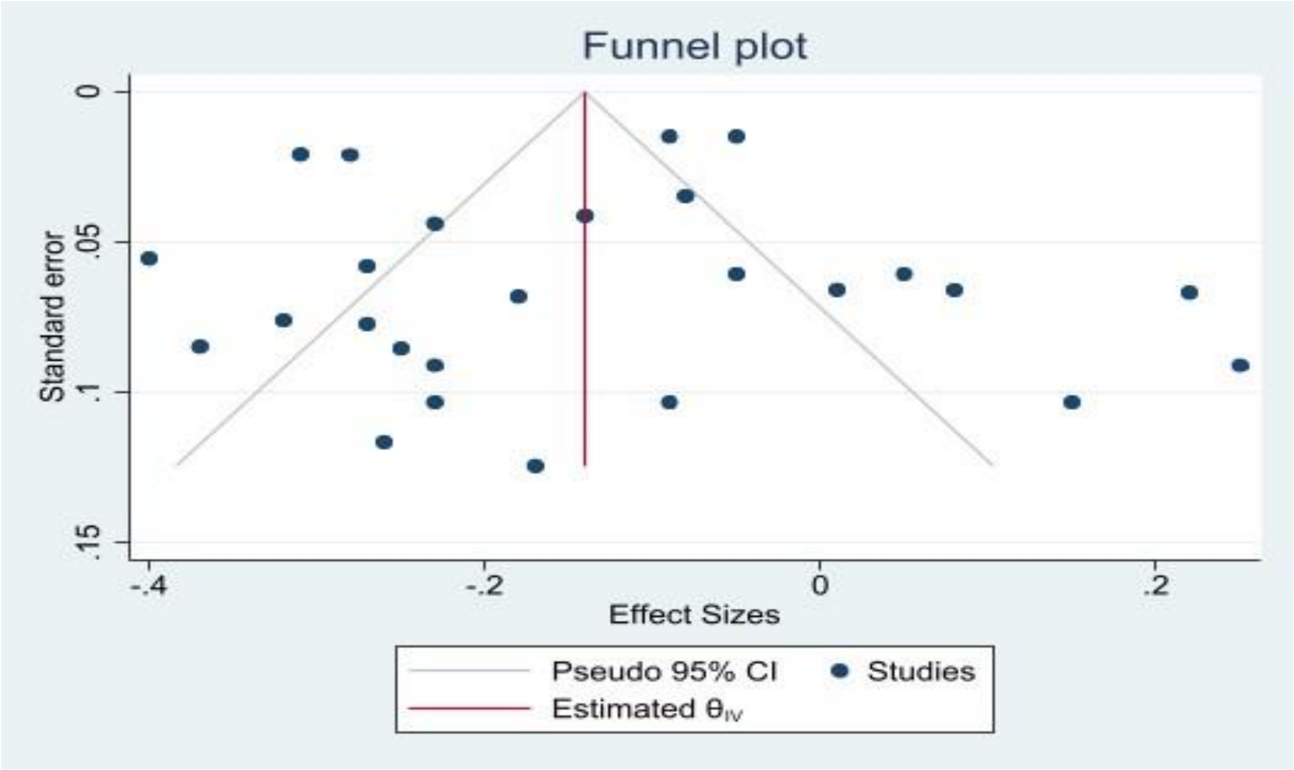

Supplementary Figure S20. Job control funnel plot

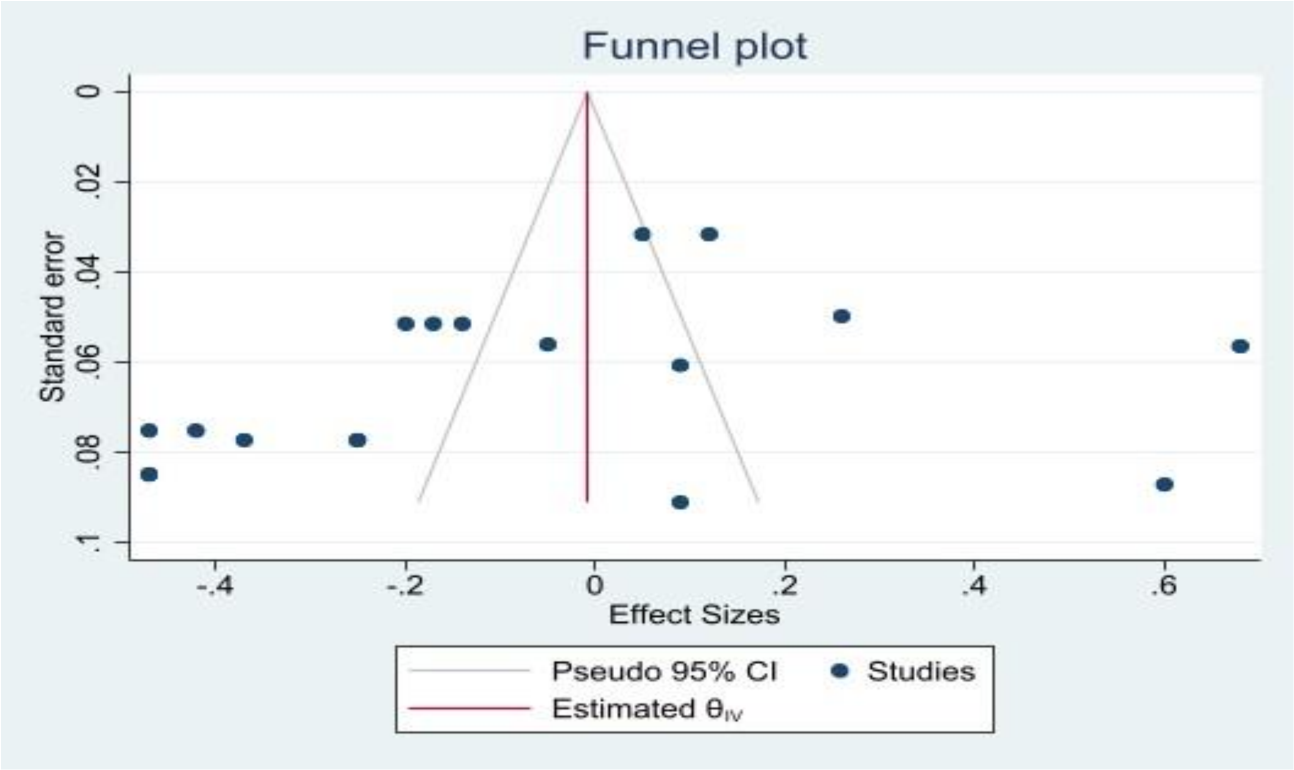

Supplementary Figure S21. Job resources funnel plot

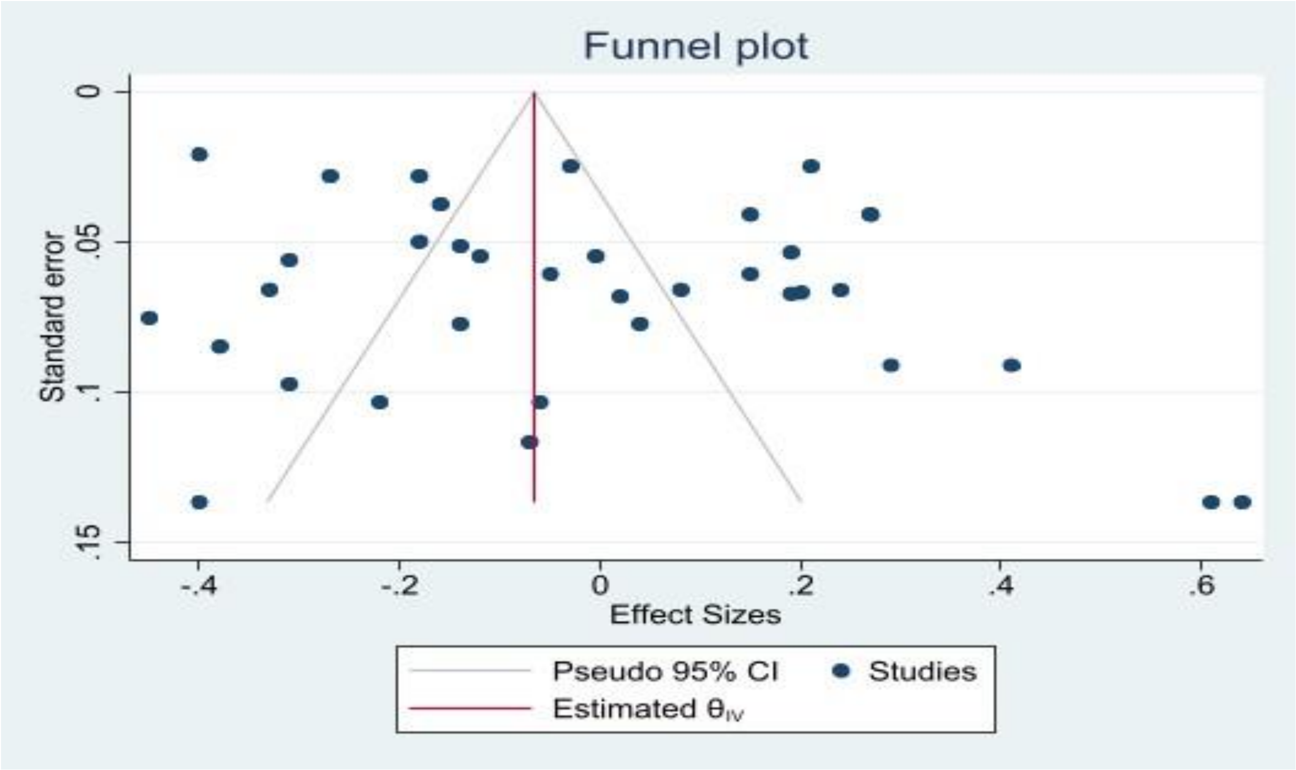

Supplementary Figure S22. Interactions at work funnel plot

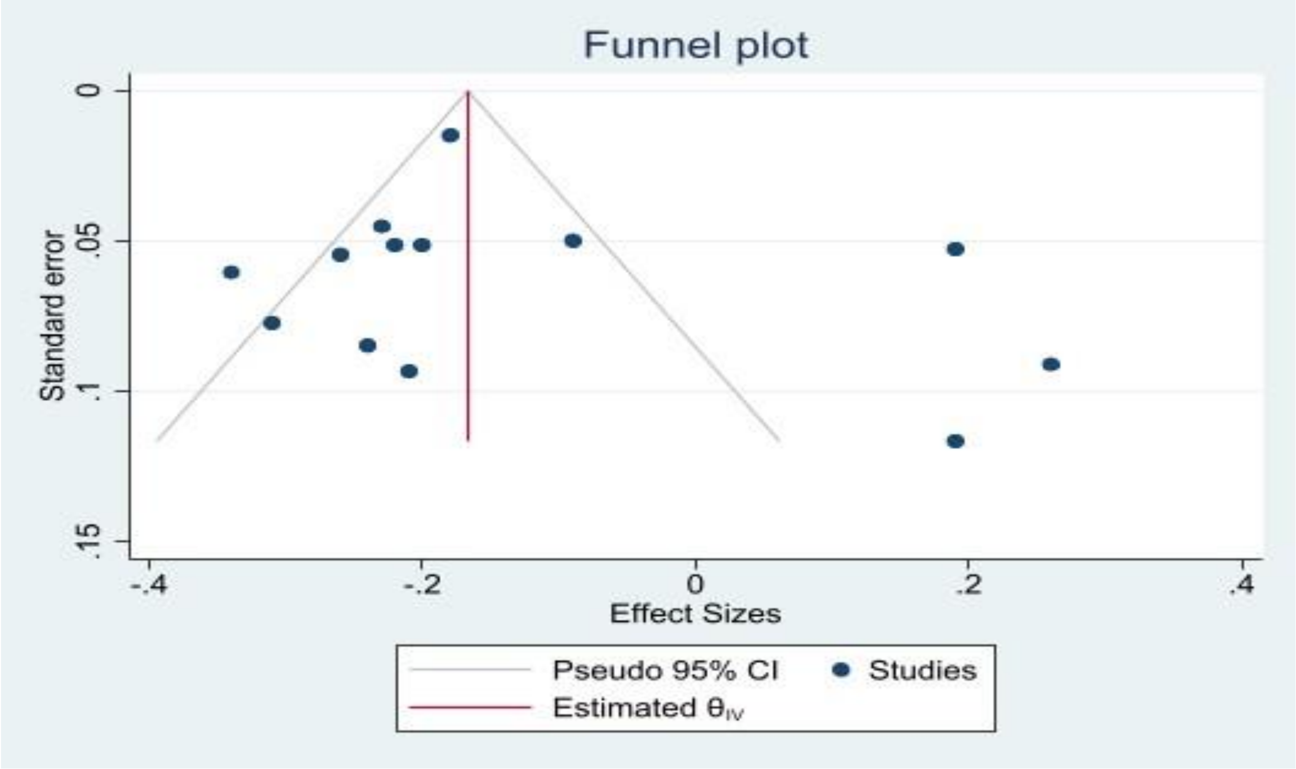

Supplementary Figure S23. Communication and leadership funnel plot

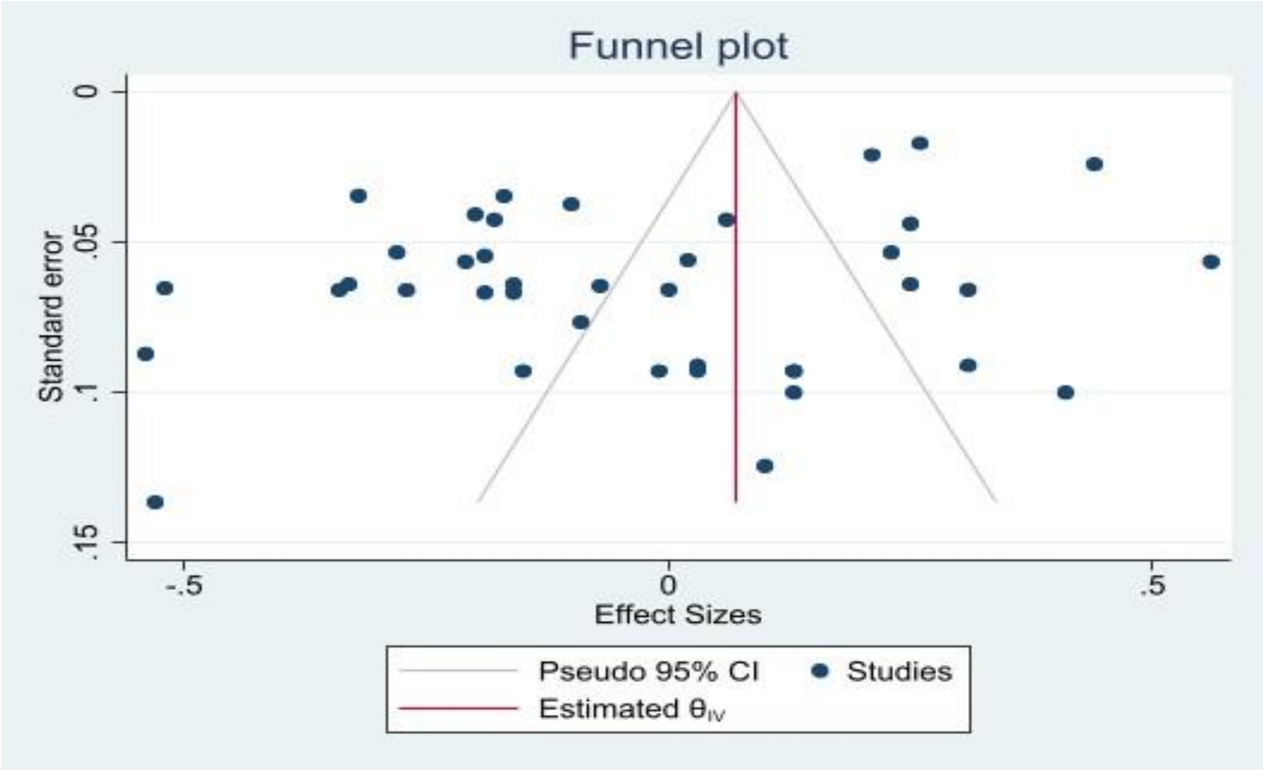

Supplementary Figure S24. Personality characteristics and self-reported health status funnel plot

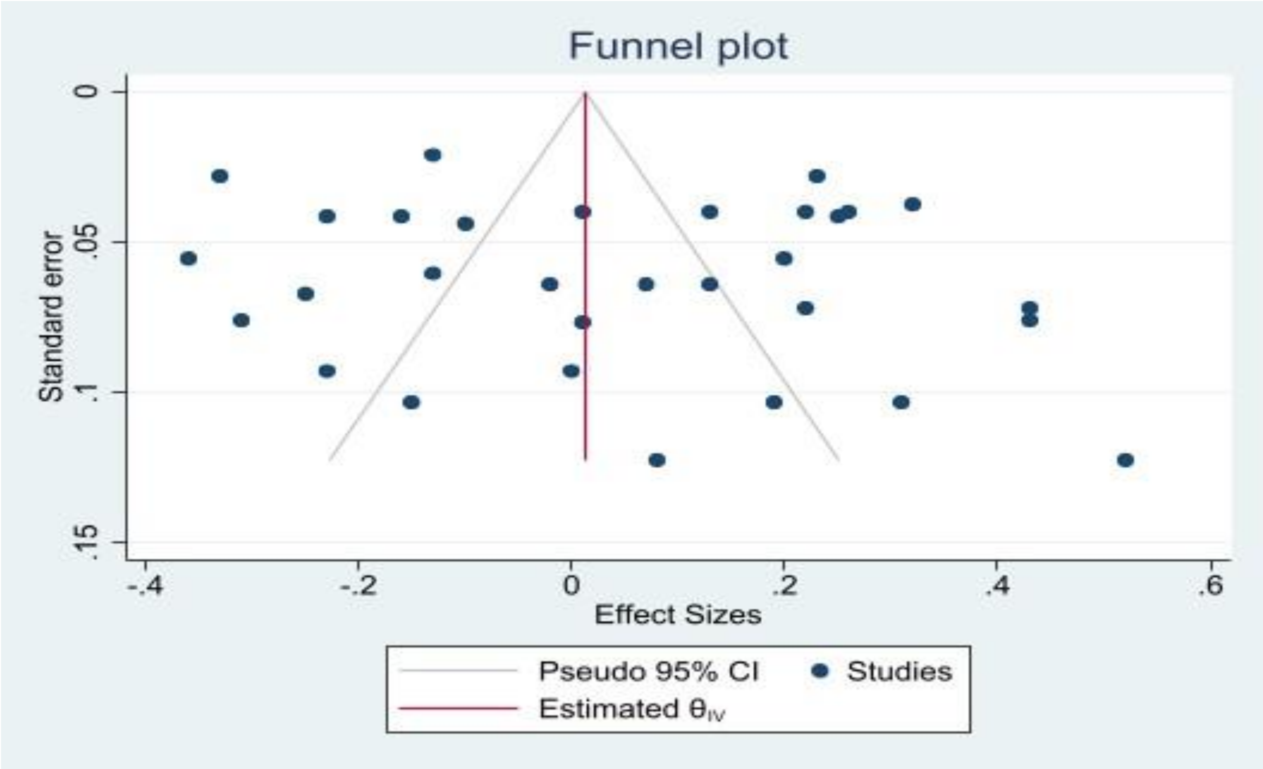

Supplementary Figure S25. Job attitudes funnel plot

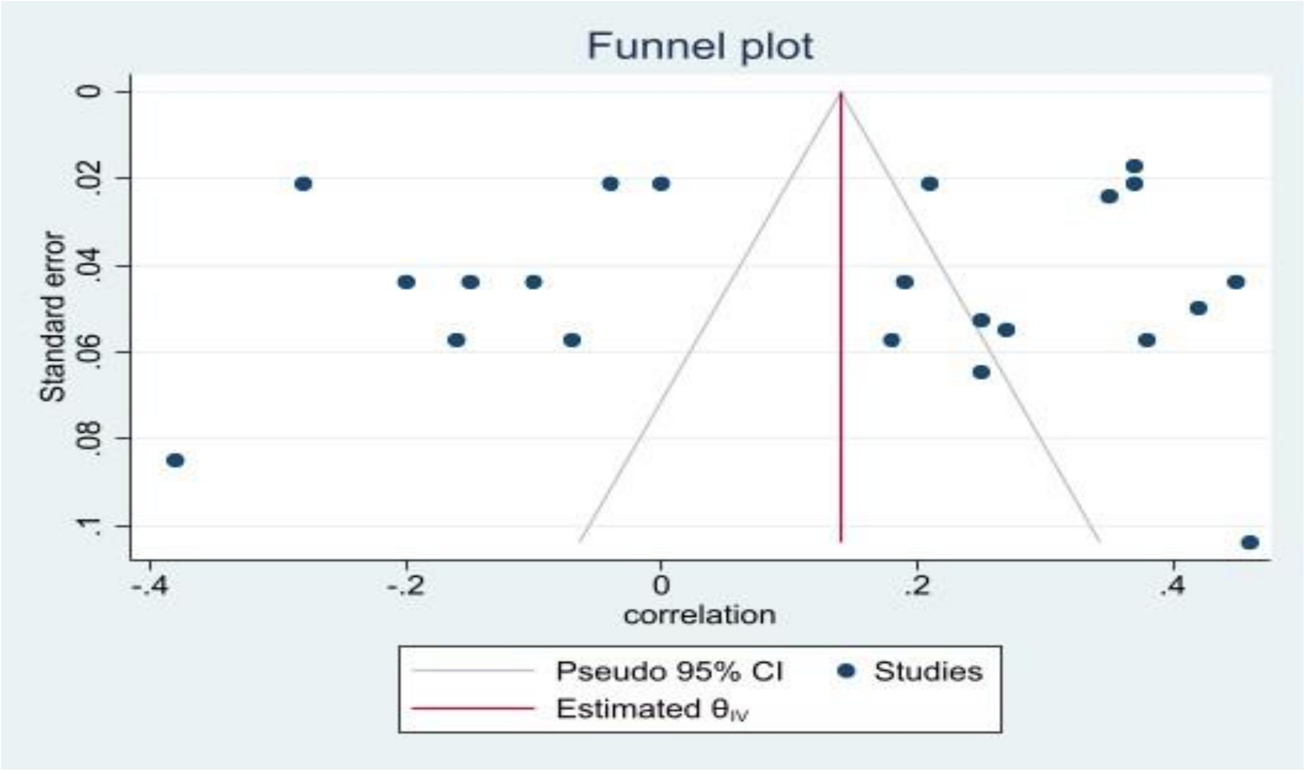

Supplementary Figure S26. Work-family interface funnel plot

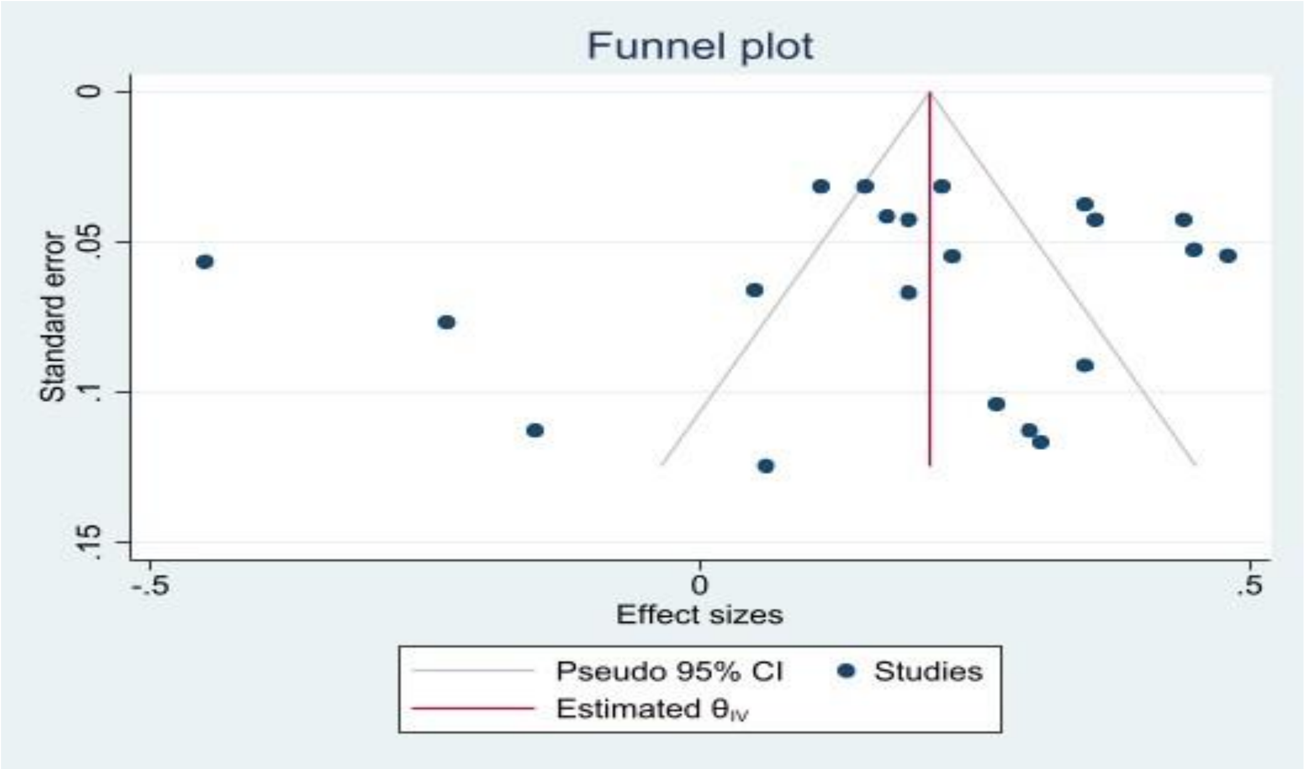

Supplementary Figure S27. Perceived intermediate work consequences funnel plot
